# Embryonic stem cell factors DPPA2/4 amplify active H3K4me3–H2AK119ub chromatin domains in non-small cell lung cancer

**DOI:** 10.1101/gad.353102.125

**Published:** 2026-05-01

**Authors:** Janith A. Seneviratne, Clare L. Crisp, Eleanor Glancy, Natalie Choy, Winnie Tan, Matthew Neve, Melanie Stammers, Tongtong Wang, Ruby Johnstone, Arshnoor Kaur, Katie A. Fennell, Marian L. Burr, Benjamin L. Parker, Shabih Shakeel, Melanie A. Eckersley-Maslin

**Affiliations:** 1 Peter MacCallum Cancer Centre, Melbourne, Victoria 3000, Australia;; 2 Sir Peter MacCallum Department of Oncology, The University of Melbourne, Melbourne, Victoria 3000, Australia;; 3 Walter and Eliza Hall Institute, Parkville, Victoria 3052, Australia;; 4 Department of Medical Biology, The University of Melbourne, Melbourne, Victoria 3052, Australia;; 5 Australian Research Council, Centre for Cryo-Electron Microscopy of Membrane Proteins, Bio21 Molecular Science and Biotechnology Institute, University of Melbourne, Parkville, Victoria 3052, Australia;; 6 Babraham Institute, Babraham, Cambridge CB22 3AT, United Kingdom;; 7 The John Curtin School of Medical Research, The Australian National University, Canberra, Australian Capital Territory 2601, Australia;; 8 Department of Anatomical Pathology, ACT Pathology, Canberra Health Services, Canberra, Australian Capital Territory 2605, Australia;; 9 Department of Anatomy and Physiology, The University of Melbourne, Melbourne, Victoria 3010, Australia;; 10 Department of Biochemistry and Pharmacology, The University of Melbourne, Melbourne, Victoria 3052, Australia

**Keywords:** DPPA2/4, H2AK119ub, NSCLC, chromatin, polycomb, priming factors

## Abstract

Embryonic regulators are often re-expressed in cancers; however, the functional and molecular significance of this is not always understood. The epigenetic priming factors developmental pluripotency-associated 2 and 4 (DPPA2/4) have crucial roles in early development and are implicated in cancer pathogenesis. We reveal that in non-small cell lung cancer (NSCLC), DPPA2/4 coexpression is associated with poorly differentiated tumors and impaired patient outcomes. Biochemically, human DPPA2/4 multimerize for their protein stability and enhanced nucleosome binding activity. In NSCLC cells, DPPA2/4 bind CG-rich sequences including promoters of developmental genes, Wnt signaling, and catabolic genes. Chromatin state modeling revealed that DPPA2/4 preferentially bind active H3K4me3 and H3K27ac domains that were intriguingly also enriched for PRC1 and its product, H2AK119ub, which was validated by H3K4me3–H2AK119ub sequential ChIP. Knockdown experiments revealed that DPPA2/4 were required to maintain RING1B and H2AK119ub at these domains. Surprisingly, despite the presence of PRC2.1, these regions lacked any detectable H3K27me3, suggesting an uncoupling between the recruitment of PRC2 to chromatin and its catalytic product. When exogenously overexpressed in NSCLC cells where they are not normally present, DPPA2/4 bind to and promote active chromatin states, resulting in an increase in in vivo xenograft tumor growth. Our results demonstrate how, in NSCLC cells, DPPA2/4 act as molecular amplifiers of active and poised chromatin. Together, this highlights how aberrant reactivation of embryonic factors in cancers may take on new functions, promoting tumorigenesis.

## Abstract (precis)

In this study, Seneviratne et al. report a role for embryonic epigenetic priming factors DPPA2 and DPPA4 in non-small cell lung cancer (NSCLC). DPPA2/4 multimerize for enhanced stability and nucleosome binding at active gene regulatory regions and amplify active and poised chromatin states, which, when aberrantly reactivated in NSCLC cells, promote tumorigenesis.

Cancers often undergo dedifferentiation and upregulate transcription and chromatin regulators associated with stem cells and embryonic states. This has been linked with increased plasticity and aggressiveness and, ultimately, impaired survival outcomes (Hölzel et al. 2013; Friedmann-Morvinski and Verma 2014; Aiello and Stanger 2016; Yuan et al. 2019; Boumahdi and de Sauvage 2020; Li and Stanger 2020; Hanahan 2022; Sharma et al. 2022). While we often have a strong understanding of the molecular roles and functions of these embryonic factors in developmental and healthy contexts, for many, it remains unknown whether they have similar roles in the context of cancer.

Developmental pluripotency-associated 2 (DPPA2) and DPPA4 are a pair of functionally intertwined proteins with essential roles during early embryonic development (Madan et al. 2009; Nakamura et al. 2011; Chen et al. 2021; Kubinyecz et al. 2021). These nonenzymatic nuclear proteins contain SAP and C-terminal domains predicted to bind nucleic acids and histones, respectively (Masaki et al. 2007). Their heterodimerization is thought to be important for their function, and depleting either DPPA2 or DPPA4 in mouse cell lines or embryos has the same phenotypic consequences as depleting both (Chen et al. 2021; Kubinyecz et al. 2021). DPPA2/4 are coexpressed predominantly in the germline and early embryo and are silenced by DNA methylation during gastrulation in mice (Maldonado-Saldivia et al. 2007; Monk et al. 2008; Eckersley-Maslin et al. 2019; Kubinyecz et al. 2021). Despite this tightly restricted expression, maternal and zygotic knockouts of DPPA2/4 survive the period of embryogenesis when they are normally expressed only to succumb shortly after birth (Madan et al. 2009; Nakamura et al. 2011; Chen et al. 2021; Kubinyecz et al. 2021). This paradoxical uncoupling between the time of expression and phenotype is a defining feature of epigenetic priming factors that facilitate poised chromatin states to enable future gene expression programs (Eckersley-Maslin 2020).

In mouse embryonic stem cells (mESCs), DPPA2/4 prime the epigenome to enable future gene expression programs and cellular transitions. Here, DPPA2/4 associate with bivalent promoters that contain both active H3K4me3 and repressive H3K27me3 histone modifications (Eckersley-Maslin et al. 2020; Gretarsson and Hackett 2020). Depleting DPPA2/4 leads to the loss of both bivalent chromatin marks and the accumulation of repressive DNA methylation, preventing these genes from timely activation upon differentiation (Eckersley-Maslin et al. 2020; Gretarsson and Hackett 2020). Overexpression of DPPA2/4 increases induced pluripotent stem cell reprogramming efficiency (Hernandez et al. 2018), suggesting that these proteins promote cellular plasticity more broadly. However, we have a limited understanding of the molecular functions of DPPA2/4 in nondevelopmental contexts.

In cancer, increased expression of DPPA2 or DPPA4 has been reported in multiple tumor types, including lung (John et al. 2008), ovarian (Tchabo et al. 2009; Deb et al. 2010), colorectal (Amini et al. 2014; Raeisossadati et al. 2014; Ghodsi et al. 2015; Zhang et al. 2015), bladder (Amini et al. 2014), prostate (Amini et al. 2014), and gastric (Shabestarian et al. 2016) cancers, and is associated with reduced overall survival. Human DPPA4 can act as an oncogene and transform fibroblasts (Tung et al. 2013), though the mechanisms by which this occurs remain unclear. Despite their coexpression in embryos (Maldonado-Saldivia et al. 2007; Eckersley-Maslin et al. 2019), DPPA2/4 have not been investigated together within a cancer context. It is also not known whether the developmental molecular roles for DPPA2/4 are conserved in human cancers or whether the proteins take on new functions in a cancer context.

Here, we investigate the functional and molecular roles of DPPA2/4 in human cancers using multiomic and biochemical assays to test the hypothesis that DPPA2/4 prime the cancer cell epigenome to facilitate cancer plasticity. Our results point toward a model whereby DPPA2/4 function together as molecular amplifiers of active and poised chromatin states, enabling more efficient expression of stem cell-related gene expression programs in cancer cells. Moreover, we uncovered an uncoupling between the PRC1-deposited H2AK119ub and PRC2-catalyzed H3K27me3 modifications. Together, our study reveals how aberrant reactivation of the embryonic priming factors DPPA2/4 facilitate active chromatin states in cancer cells to potentially fuel dedifferentiated aggressive phenotypes.

## Results

### Coexpression of the embryonic regulators DPPA2/4 in cancers correlates with dedifferentiated states and impaired survival

**Figure 1.**
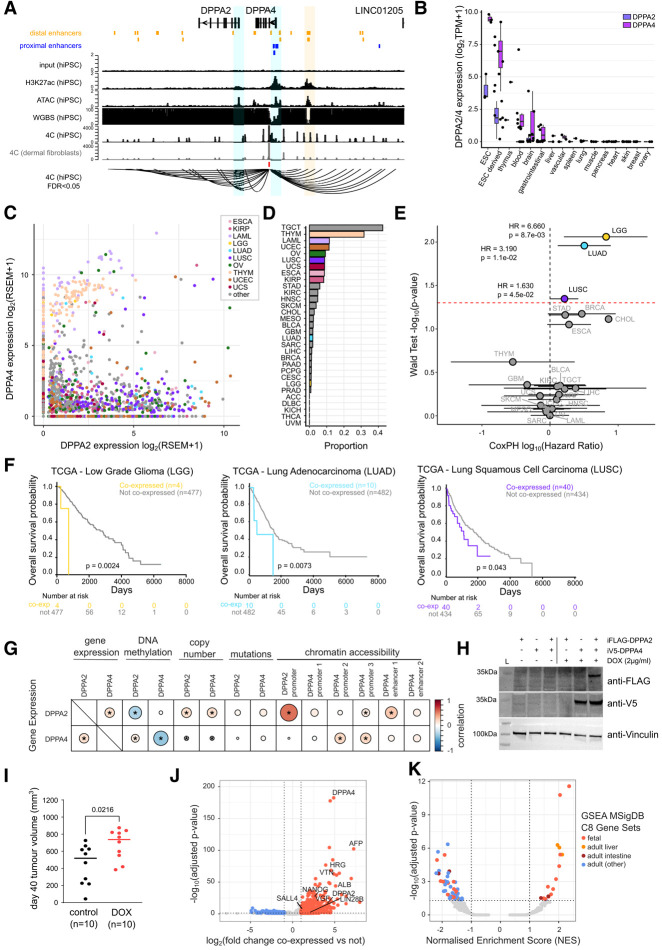
Coexpression of the embryonic regulators DPPA2/4 in cancers correlates with dedifferentiated states and impaired survival. (*A*) Overview of the DPPA2/4 genomic locus (hg38; chromosome 3:109,238,670–109,410,877) depicting gene structures and ENCODE annotated distal (orange) or proximal (blue) enhancers. Tracks visualize matched genomic input and ChIP-seq for H3K27ac (in counts per million per base pair), chromatin accessibility (ATAC-seq in counts per million per base pair), and DNA methylation (whole-genome bisulfite sequencing [WGBS], 0%–100% DNA methylation) in hiPSCs (reanalyzed from GSE145964 [Yan et al. 2020]). The *bottom* tracks visualize 4C-seq following DPPA4 capture in human iPSCs and matched dermal fibroblasts (bait region in red), where axes represent counts per million per base pair-normalized read density (averaged between two replicates) of chimeric reads spanning the capture region and nearby *cis*-interacting regions (reanalyzed from GSE90782 [Ikeda et al. 2017]). A Sashimi track summarizes significant *cis* interactions (FDR <0.05) in hiPSCs as determined by r3Cseq. A putative cluster of enhancers that interact with DPPA4 are highlighted in light orange. (*B*) Box plots of DPPA2/4 expression (transcripts per million [TPM]) in a range of human cell line models spanning human development from the Epigenetic Roadmap Consortium. Cell line types are ordered by decreasing average DPPA2/4 expression. (*C*) Scatter plot visualizing DPPA2 (*X*-axis) and DPPA4 (*Y*-axis) gene expression from RNA-seq across TCGA patients across all cohorts (excluding testicular germ cell tumor [TGCT] cases); selected cohorts are depicted in different colors. Data are represented as log_2_(RSEM+1). (*D*) Bar plot displaying the proportion of DPPA2/4 coexpressors per cancer cohort (except for the TGCT cohort). (*E*) Scatter plot of TCGA cancer cohort Cox proportional hazard (CoxPH) modeling of patient outcomes. Patients in each cohort are subdivided by the coexpression of DPPA2 and DPPA4 as determined by RNA-seq (median of all DPPA2 or DPPA4 expressors [RSEM >0]). The *Y*-axis indicates the statistical significance of CoxPH models (unadjusted *P*-value of the Wald test for each CoxPH model, −log_10_ transformed); the red line indicates the threshold for *P* < 0.05. The *X*-axis indicates the hazard ratio (HR) of CoxPH models (log_10_ transformed). Colored dots indicate those models with HR >1 and *P*-value of <0.05, and whiskers represent the lower and upper 95% confidence intervals of HRs for each model. (*F*) Kaplan–Meier curves demonstrating overall survival of patients stratified as DPPA2/4 coexpressed and lowly/not expressed in low-grade glioma (LGG; *n* = 481), lung adenocarcinoma (LUAD; *n* = 492), and lung squamous cell carcinoma (LUSC; *n* = 474) TCGA cohorts. (*G*) Dot plot visualizing the correlation coefficient of DPPA2/4 gene expression (RNA-seq) correlated with their gene expression (RNA-seq), promoter DNA methylation (methylation arrays), gene copy number (WES), gene somatic mutation (WES), and chromatin accessibility at promoters or distal enhancers (ATAC-seq) in all TCGA cancer cohorts (excluding the TGCT cohort). Biserial correlation is used for mutations, and Pearson correlation is used for all others. Significant correlations (BH-adjusted *P*-value of <0.05) are marked with an asterisk. (*H*) Western blot of whole-cell extracts from NCI-H1299 cells following stable integration of DOX-inducible FLAG-DPPA2 and/or V5-DPPA4 constructs and induction with a vehicle control (H_2_O) or 2 µg/mL doxycycline (DOX) for 72 h. Blots show anti-FLAG, anti-V5, or antivinculin (loading control). “L” indicates a molecular ladder; sizes are annotated at the *left*. (*I*) Tumor volumes (cubic millimeters) from BALB/c nude mice xenografted with NCI-H1299 DPPA2/4 double-overexpression lines (iFLAG-DPPA2 and iV5-DPPA4) at day 40 (last day when all mice within the study were alive). Lines were pretreated with a vehicle control (H_2_O) or 2 µg/mL doxycycline (DOX) for 72 h prior to engraftment, and xenografted mice were kept on control or doxycycline feed, respectively, to maintain overexpression. (*J*) Volcano plot comparing gene expression differences between DPPA2/4-coexpressing and other NSCLC (LUAD+LUSC) tumors. Differentially expressed genes are those with log_2_ fold change of less than −1 (down) or >1 (up) and adjusted *P*-value of <0.05 (edgeR accounted for tumor type differences [∼type+group]). (*K*) Volcano plot illustrating MSigDB C8 gene sets after a GSEA using all genes ranked by log_2_FC comparing DPPA2/4-coexpressing and other NSCLC (LUAD+LUSC) tumors. Depleted gene sets were determined as those with NES<−1 and adjusted *P*-value of <0.05, and enriched gene sets were determined as NES >1 and adjusted *P*-value of <0.05. Enriched gene sets falling into broad categories are annotated.

The embryonic epigenetic priming factors DPPA2/4 are uniquely located in tandem on human chromosome 3 and are highly coexpressed in early development (Maldonado-Saldivia et al. 2007; Eckersley-Maslin et al. 2019) (Fig. 1A). Upon cell fate establishment, DPPA2/4 expression becomes repressed in most postembryonic tissues (Fig. 1B), with the notable exception of the testes (Supplemental Fig. S1A). In contrast, analysis of The Cancer Genome Atlas (TCGA) revealed high expression of DPPA2 and/or DPPA4 in a range of different tumor types (Fig. 1C,D; Supplemental Fig. S1B,C). To assess clinical significance of DPPA2/4 coexpression, we performed univariate Cox proportional hazard (CoxPH) modeling. While testicular germ cell tumors (TGCTs) and thymomas (THYMs) had the highest proportion of coexpression (Fig. 1D), this was not prognostic (Fig. 1E), likely due to DPPA2/4 being expressed in the normal counterparts. In contrast, for low-grade glioma (LGG), lung adenocarcinoma (LUAD), and lung squamous cell carcinoma (LUSC), coexpression was associated with worse overall patient survival (hazard ratio [HR]: 1.63–6.66, *P* < 0.05) (Fig. 1E,F; Supplemental Fig. S1D). This was not strongly influenced by any clinical factors including sex, age, disease stage, smoking status, prior malignancy, tumor purity, or mutations upon multivariate testing (Supplemental Fig. S1E). We also performed CoxPH modeling when cohorts were subdivided by DPPA2 or DPPA4 expression alone using the same expression cutoffs and observed that only LGG retained prognostic significance, as opposed to LUSC and LUAD cohorts (Supplemental Fig. S1F,G), suggesting that expression of both genes is necessary to confer deleterious phenotypes in non-small cell lung cancer (NSCLC).

We next sought to understand how DPPA2/4 become re-expressed in tumors. In normal somatic tissues, the DPPA2/4 locus is methylated (Supplemental Fig. S1H), suggesting that DNA hypomethylation, frequently observed in cancer (Ehrlich 2009), may lead to DPPA2/4 reactivation. We analyzed DPPA2/4 promoter methylation along with copy number, somatic mutation, and chromatin accessibility (Supplemental Fig. S1C) and correlated these with gene expression across all tumor types. TGCTs were analyzed separately as they arise from a tissue where DPPA2/4 are already highly expressed (Supplemental Fig. S1A). As expected, expression of DPPA2 and expression of DPPA4 were positively correlated with one another (*r* = 0.17, adjusted *P* < 0.05), supporting their coregulation, while chromatin accessibility at either promoter or at the putative enhancer also weakly correlated with gene expression (Fig. 1G). Overall promoter methylation was highly anticorrelated with gene expression (DPPA2: *r* = −0.301, DPPA4: *r* = −0.39, adjusted *P* < 0.05) (Fig. 1G; Supplemental Fig. S1I). We also observed modest somatic mutation frequency (nine out of 31 cancer types, 0.6%–6.2%) and variable frequency of copy number gains (29 out of 31 cancer types, 0.4%–66%); however, these were only weakly correlated with gene expression (Fig. 1G; Supplemental Fig. S1I). These correlations were even more pronounced for TGCTs (Supplemental Fig. S1I), reflecting the strong expression of DPPA2/4 in healthy testes (Supplemental Fig. S1A) from which TGCTs arise. Taken together, these data suggest that promoter hypomethylation enables DPPA2/4 coexpression in cancer and is associated with worse overall survival outcomes in low-grade glioma and non-small cell lung cancer (NSCLC) patients.

To assess whether DPPA2/4 coexpression promotes tumor growth in lung cancer, we established a doxycycline-inducible system to coexpress both DPPA2 and DPPA4 in NCI-H1299 non-small cell lung cancer cells that normally do not express these proteins for xenograft experiments (Fig. 1H). Cells were treated with doxycycline for 3 days prior to subcutaneous engraftment in BALB/c nude mice, and DPPA2/4 coexpression was maintained during tumor formation and growth through DOX chow feed. Both control and DOX-treated tumors were poorly differentiated. At day 40 after engraftment, when all animals were still alive, there was a significant increase in tumor volume of DOX-treated tumors compared with controls (*P* = 0.0216) (Fig. 1I; Supplemental Fig. S1J,K). Similarly, shRNA depletion of DPPA4 alone or in combination with DPPA2 (see the description of the shRNA model below) in NCI-H661 cells after xenograft establishment modestly slowed tumor growth in vivo (Supplemental Fig. S1L,M). This supports a role for DPPA2/4 coexpression in promoting tumor growth in non-small cell lung cancer, ultimately impairing overall survival for these patients.

We next performed differential gene expression analysis to understand the potential consequences of DPPA2/4 coexpression in NSCLC patients. There were approximately five times more upregulated (*n* = 1049) than downregulated (*n* = 206) genes in DPPA2/4-coexpressing NSCLC tumors (|log_2_FC| >1, adjusted *P* < 0.05). (Fig. 1J; Supplemental Table S1). Among the upregulated genes were pluripotency markers such as NANOG and LIN28B, as well as several hepatic oncofetal genes such as AFP (Sharma et al. 2022). Gene set enrichment analyses (GSEAs) revealed an enrichment of cellular phenotypes associated with fetal development and regenerative adult tissues and a depletion of phenotypes associated with nonregenerative adult tissues (Fig. 1K; Supplemental Fig. S1N; Supplemental Table S2). This collectively suggests that tumors with DPPA2/4 coexpression recapitulate oncofetal transcriptional programs (Sharma et al. 2022).

### DPPA2/4 multimerize for stability and nucleosome binding

**Figure 2.**
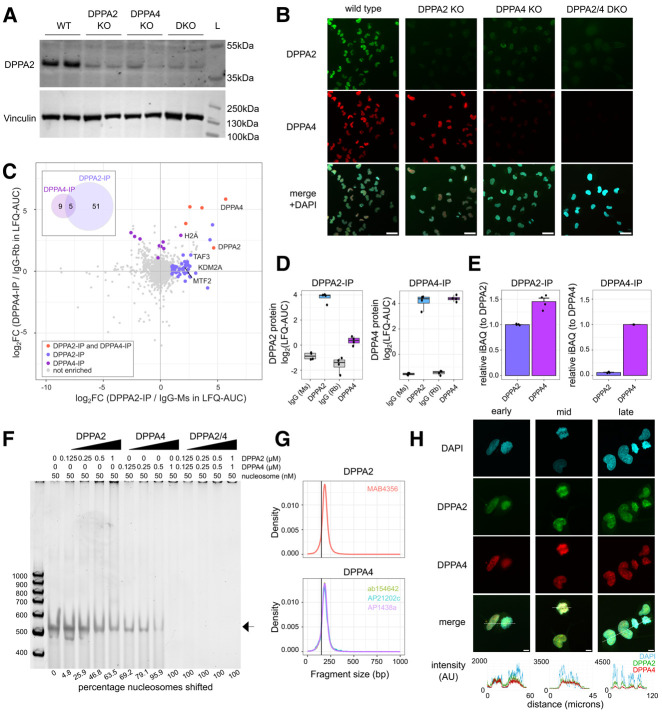
DPPA2/4 multimerize for stability and nucleosome binding. (*A*) Western blot of whole-cell extracts from NCI-H661 cells following CRISPR–Cas9-mediated knockout of DPPA2 and/or DPPA4 isogenic clones (WT) Wild type, (DPPA2 KO) DPPA2 single knockout, (DPPA4 KO) DPPA4 single knockout, (DKO) DPPA2+4 double knockout. Blots are anti-DPPA2 or antivinculin. “L” indicates a molecular ladder; sizes are annotated at the *right*. (*B*) Immunofluorescence images for DPPA2 (green) and DPPA4 (red) in NCI-H661 isogenic knockout clones. Single *z*-slice representative images are provided for each genotype. Scale bars, 50 µm. (*C*) Scatter plot of immunoprecipitation mass spectrometry (IP-MS) results following endogenous DPPA2 or DPPA4 IP from NCI-H661 whole-cell lysates (*n* = 4 per condition). Enriched proteins were determined relative to species-matched IgG IP control by comparing label-free quantitation (LFQ-AUC) for each protein (log_2_FC ≥1 and BH-adjusted *P*-value of <0.05). The *inset* Venn diagram highlights the overlap of enriched proteins for DPPA2 and DPPA4. Proteins are colored based on whether they were enriched in a single IP or both IPs. (*D*) Box plots of enriched DPPA2 and DPPA4 protein levels from IP-MS of DPPA2/4 and matched IgG controls from NCI-H661 whole-cell lysates (log_2_LFQ-AUC; *n* = 4 per condition). (*E*) Box plots of DPPA2 and DPPA4 absolute protein levels relative to the bait (DPPA2/4 stoichiometry) following IP-MS of DPPA2/4 from NCI-H661 whole-cell lysates (units are relative intensity-based absolute quantification [iBAQ]; *n* = 4 per condition). (*F*) Representative electrophoretic mobility shift assay (EMSA) of unmodified recombinant nucleosomes incubated in the absence/presence of increasing concentrations of recombinant DPPA2-Myc/FLAG and/or His-DPPA4 protein. Protein concentrations are denoted *above* the gel, and the percentage of nucleosomal DNA bound in each lane is annotated *below*. Percentage nucleosomes shifted represents the percentage loss of band compared with the input. The experiment was repeated three times. (*G*) Density histogram of enhanced chromatin occupancy (EChO) paired-end fragment size analysis from CUT&RUN experiments performed using DPPA2 (MAB4356) and DPPA4 (ab154642, AP21202c, and AP1438a) antibodies. The *X*-axis represents the mean CUT&RUN fragment size at peak foci (peaks called using MACS2 for each CUT&RUN relative to a matched IgG control). The vertical line at 150 bp represents the cutoff for nucleosomal DNA fragments. (*H*) Immunofluorescence imaging of DPPA2 (green) and DPPA4 immunostaining (red) in NCI-H661 parental cells. Representative images are projections (maximum five *z*-slices) for cells in different stages of mitosis. DAPI stain is shown in blue. Scale bars, 10 µm. The intensity profile for each channel in a single *z*-slice along the dotted line in the merged image is shown *below* the images.

To study the molecular and cellular functions of DPPA2/4 in a cancer context, we surveyed transcriptomic (Cancer Cell Line Encyclopedia) and proteomic (Gonçalves et al. 2022) data and identified NCI-H661 as one of the very few cancer cell lines that coexpressed DPPA2 and DPPA4 at both the mRNA and protein levels (Supplemental Fig. S2A,B). NCI-H661 was recently reclassified as a SMARCA4-deficient NSCLC cell line and forms undifferentiated tumors in vivo (Ng et al. 2024). Using this cell line, we generated a series of CRISPR single- and double-knockout clones (Fig. 2A,B; Supplemental Fig. S2C). Notably, the DPPA4 single-knockout clones had substantially reduced levels of DPPA2 protein (Fig. 2A,B; Supplemental Fig. S2D) despite similar transcript levels (Supplemental Fig. S2C). This suggests that DPPA2 protein requires DPPA4 for its stabilization. Consistently, overexpression of DPPA2 in NCI-H1299 cells did not result in detectable protein levels (Fig. 1H). In contrast, DPPA4 did not appear to require DPPA2 for its stability and was correctly localized to the nucleus in DPPA2 knockout cells (Fig. 2A,B). Thus, while DPPA2 requires DPPA4 for stability in cells, DPPA4 is stable on its own.

Next, we immunoprecipitated endogenous DPPA2 or DPPA4 in NCI-H661 cells and identified binding partners by mass spectrometry (Fig. 2C; Supplemental Fig. S2E–H; Supplemental Table S3). As expected, DPPA2 was pulled down by DPPA4 and vice versa, supporting multimerization (Fig. 2D). To gain insights into potential relative stoichiometry, we assessed intensity-based absolute quantification (iBAQ) values, where we observed that in the DPPA2 IP, the ratio between DPPA2 and DPPA4 was >1 (Fig. 2E). The converse in the DPPA4 IP was not true, with DPPA2 present at substoichiometric levels relative to DPPA4 (Fig. 2E). Thus, while DPPA2 is always found in complex with one or more DPPA4 molecules, DPPA4 may have DPPA2-independent roles. Consistently, mass photometry analysis showed that only DPPA4 can exist as a monomer in vitro, while DPPA2 and DPPA4 form multimers when on their own and higher-order complexes when together (Supplemental Fig. S2I,J). Together, our data support the existence of stable DPPA4 mono/multimers as well as the canonical DPPA2/4 heterocomplex in NCI-H661 NSCLC cells.

The proteomic analysis revealed 64 proteins that interact with DPPA2 and/or DPPA4 at substoichiometric levels (Fig. 2C), supporting previous findings in mouse ESCs (Hernandez et al. 2018; Eckersley-Maslin et al. 2020) that these proteins are not stable components of larger complexes. Similar to mouse ESCs (Supplemental Fig. S2K; Hernandez et al. 2018; Eckersley-Maslin et al. 2020), we observed association with MTF2 from the Polycomb repressive complex 2 as well as the lysine demethylase KDM2A, which removes H3K36me2 (Fig. 2C; Supplemental Fig. S2L–N; Tsukada et al. 2006). Of interest was a strong association specifically with histone H2A but not other histones (Fig. 2C; Supplemental Fig. S2O,P), suggesting that DPPA2/4 may be interacting with nucleosomes rather than naked DNA. To test this, we performed electrophoretic mobility shift assay (EMSA) using recombinant DPPA2 and/or DPPA4, which revealed that DPPA4 and, to a much lesser extent, DPPA2 were able to bind nucleosome–DNA complexes in vitro, with the heterodimer exhibiting very strong binding affinity (Fig. 2F; Supplemental Fig. S2Q). Supporting nucleosome binding ability, EChO analysis (Meers et al. 2019) of endogenous DPPA2/4 CUT&RUN (see below) revealed that DPPA2/4-bound fragments were approximately mononucleosomal in length (Fig. 2G). Furthermore, and consistent with previous observations in mice (Masaki et al. 2007; Kubinyecz et al. 2021), human DPPA2/4 were associated with mitotic chromatin in wild-type NCI-H661 cells (Fig. 2B,H). Together, these results suggest that DPPA4 and DPPA2/4 multimers bind nucleosomes, possibly to facilitate the recruitment and/or stabilization of chromatin-modifying complexes at these loci.

### DPPA2/4 colocate at active gene regulatory regions marked by H3K4Me3, H3K27ac, and H2AK119ub

**Figure 3.**
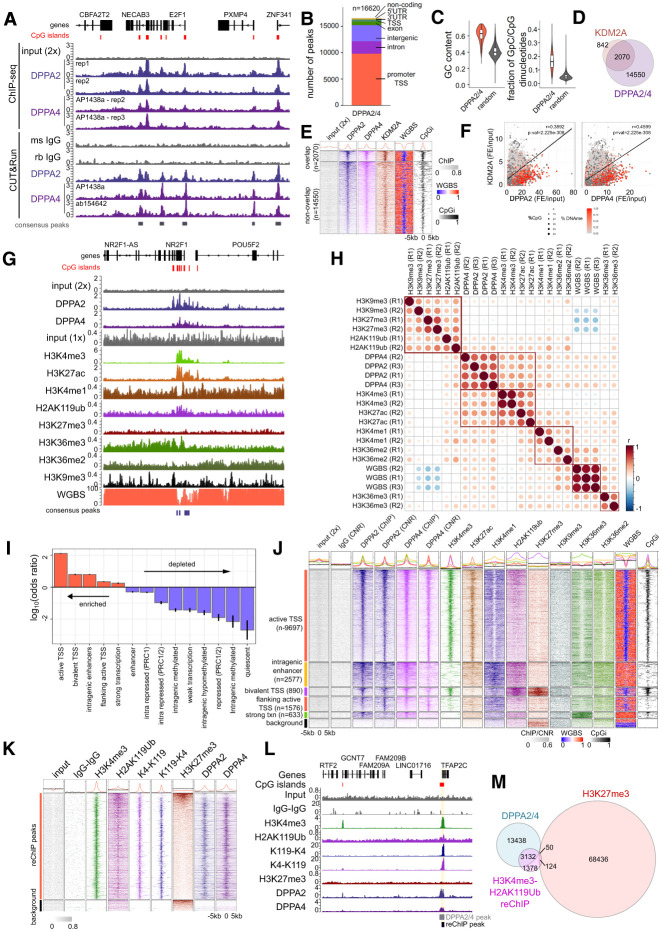
DPPA2/4 colocate at active gene regulatory regions marked by H3K4me3, H3K27ac, and H2AK119ub. (*A*) Genome view of the chromosome 20: 33,632,176–33,752,019 (hg38) locus. Gene annotation track and CpG islands (red) are shown *above*. Tracks depict individual replicate tracks of double-cross-linked input ChIPs (*top*) and CUT&RUN (*bottom*). ChIP tracks include control input (gray; *n* = 1), DPPA2 (blue; *n* = 2), and DPPA4 (purple; *n* = 2). CUT&RUN tracks include mouse and rabbit IgG (gray; *n* = 1 each), DPPA2 (blue; *n* = 1), and DPPA4 (two different antibodies; purple; *n* = 1). Consensus DPPA2+4 peaks are annotated at the *bottom*. Scales are in counts per million per base pair. (*B*) Genomic annotations of DPPA2+4 consensus peaks (*n* = 16,620). Promoter TSS is defined as the TSS ± 1500 bp. (*C*) G/C content of (*left*) and GC/CG dinucleotide fraction within (*right*) DPPA2+4 consensus peaks (*n* = 16,620) compared with size-matched random genomic regions (*n* = 16,620). (*D*) Venn diagram showing the overlap of DPPA2/4 consensus peaks and KDM2A peaks. (*E*) Heat maps of ChIP-seq enrichment at DPPA2+4 consensus peaks (±5 kb from the center of each peak, split into 100 equal windows) grouped by KDM2A peak overlap (*top*) versus nonoverlap (*bottom*) for input (gray; *n* = 1), DPPA2 (purple; *n* = 2), DPPA4 (pink; *n* = 2), and KDM2A (orange; *n* = 1). ChIP counts per million per base pair values were scaled per bin across all regions for visualization. DNA methylation (WGBS; *n* = 3) and CpG island density are shown for comparison. WGBS represents the fraction of methylated DNA. (*F*) Pearson correlation of KDM2A versus DPPA2 (*left*) or DPPA4 (*right*) enrichment at CpG islands in NCI-H661 cells. Scatter plots show fold enrichment over input and are colored by percentage DNA methylation (red). (*G*) Genome view of the chromosome 5: 93,429,332–93,810,092 (hg38) locus. Gene annotation track and CpG islands (red) are shown *above*. Shown from *top* to *bottom* are the double-cross-linked (2×) input ChIP control (*n* = 1); average DPPA2 (*n* = 2) and DPPA4 (*n* = 2) ChIP; single-cross-linked input ChIP control (*n* = 1); average H3K4me3 (*n* = 2), H3K27ac (*n* = 2), H3K4me1 (*n* = 2), H2AK119ub (*n* = 2), H3K27me3 (*n* = 2), H3K36me3 (*n* = 2), H3K36me2 (*n* = 2), and H3K9me3 (*n* = 2) ChIP; and average DNA methylation (WGBS; *n* = 3). Consensus DPPA2+4 peaks are annotated *below*. Scales are in counts per million per base pair for ChIPs and in percentage methylated DNA for WGBS (0%–100%). (*H*) Genome-wide pairwise correlations of ChIP-seq (DPPA2, DPPA4, H3K4me3, H3K27ac, H3K4me1, H3K27me3, H2AK119ub, H3K9me3, H3K36me2, and H3K36me3 fold enrichment over the input control) and WGBS (fraction of methylated DNA) partitioned into 200 bp bins genome-wide. Dots are colored and sized by Pearson correlation coefficients (*r*) and ordered by hierarchical clustering, with red squares denoting major clusters. (*I*) Odds ratio tests of DPPA2+4 consensus regions in association with ChromHMM chromatin states (17-state model; three null states [i.e., no overlap with DPPA2+4 consensus regions] excluded). Tests reflect the representation of DPPA2+4 regions in a given chromatin state compared with all other DPPA2+4 regions falling within all other chromatin state annotations. States are ordered by decreasing odds ratio, reflecting enriched chromatin states (log_10_odds ratio >0; red) compared with depleted states (log_10_odds ratio <0; blue). Error bars represent the 95% confidence intervals for hazard ratios, and all shown states have a BH-adjusted *P*-value of <0.05. (*J*) Heat maps of genomic regions centered on DPPA2+4 consensus peaks (resized to ±5 kb from the center of each peak, split into 100 equal windows) overlapping enriched chromatin states: active TSS (orange; *n* = 9676), intragenic enhancer (yellow; *n* = 2577), bivalent TSS (purple; *n* = 890), flanking active TSS (orange; *n* = 1576e), strong transcription (green; *n* = 633), and background regions not bound by DPPA2/4 for comparison (selection; black; *n* = 1000). Columns represent the double-cross-linked input ChIP control (*n* = 1); DPPA2 ChIP (*n* = 2); DPPA2 CUT&RUN (*n* = 1); DPPA4 ChIP (*n* = 2); DPPA4 CUT&RUN (*n* = 1); H3K4me3 (*n* = 2), H3K27ac (*n* = 2), H3K4me1 (*n* = 2), H2AK119ub (*n* = 2), H3K27me3 (*n* = 2), H3K9me3 (*n* = 2), H3K36me3 (*n* = 2), and H3K36me2 (*n* = 2) ChIPs; and DNA methylation (WGBS; *n* = 3). The last column depicts CpG island density. ChIP and CUT&RUN counts per million per base pair values were scaled per bin across all regions for visualization. WGBS represents the fraction of methylated DNA. (*K*) Heat maps of genomic regions centered on H3K4me3–H2AK119ub reChIP consensus peaks (resized to ±5 kb from the center of each peak, split into 100 equal windows; *n* = 4663) compared with a set of background regions (*n* = 500). Columns represent the double-cross-linked input ChIP control (*n* = 1); IgG–IgG reChIP control (*n* = 2); H3K4me3 (*n* = 2); H2AK119ub (*n* = 2); H3K4me3–H2AK119ub reChIP (K4–K119; *n* = 2); H2AK119ub–H3K4me3 reChIP (K119–K4; *n* = 2); and H3K27me3 (*n* = 2), DPPA2 (*n* = 2), and DPPA4 (*n* = 2) ChIPs. (*L*) Genome view of the chromosome 20: 56,435,664–56,677,328 (hg38) locus. Gene annotation track and CpG islands (red) are shown *above*. Tracks show input ChIP control (*n* = 1); average reChIP IgG–IgG control (*n* = 2); H3K4me3 (*n* = 2) and H2AK119ub (*n* = 2) ChIPs; H3K4me3–H2AK119ub (K4–K119; *n* = 2) and H2AK119ub–H3K4me3 (K119–K4; *n* = 2) reChIPs; and H3K27me3 (*n* = 2), DPPA2 (*n* = 2), and DPPA4 (*n* = 2) ChIPs. Consensus DPPA2/4 (gray) and reChIP peaks (black) are depicted *below*. Scales are in counts per million per base pair. (*M*) Venn diagram showing the overlap between DPPA2/4 consensus peaks, H3K4me3–H2AK119ub reChIP peaks (consensus of both K4–K119 and K119–K4 peaks), and H3K27me3 ChIP peaks.

Next, we sought to understand where DPPA2/4 bind genome-wide in a cancer context. We performed dual-cross-linked chromatin immunoprecipitation of endogenous DPPA2 or DPPA4 followed by high-throughput sequencing (ChIP-seq) in NCI-H661 cells, complementing it with native CUT&RUN (Fig. 3A; Supplemental Fig. S3A; Supplemental Table S4). This enabled us to determine DPPA2/4 binding sites with high confidence and eliminate any potential transient and/or secondary chromatin binding events that may arise with the longer fixation times required for dual-cross-linking strategies (Baranello et al. 2016). There was high concordance between DPPA2 and DPPA4 peaks called using either method, supporting their co-occupancy in the genome (Supplemental Fig. S3B,C). We defined 16,220 peaks that were bound by DPPA2 and DPPA4 in all replicates (termed DPPA2/4 peaks) with either ChIP-seq and/or CUT&RUN methodologies (Supplemental Fig. S3B) for subsequent analysis.

DPPA2/ peaks occurred predominantly at promoters (Fig. 3B; Supplemental Fig. S3D) of developmental genes, Wnt signaling, and catabolic genes (Supplemental Fig. S3E; Supplemental Table S5). Similar to those of mice, human DPPA2/4 bind CG-rich sequences (Fig. 3C) but do not have a strong DNA motif (Supplemental Fig. S3F). Consistently, 56.7% (*n* = 11,925 of 21,025) of CpG island-containing TSSs were bound by DPPA2/4 compared with just 0.04% (*n* = 662 of 15,486) of non-CpG island TSSs. This suggests that the recruitment of DPPA2/4 to its binding sites may be mediated by interacting proteins that contain CpG reader domains. One candidate was the CxxC domain-containing protein KDM2A, which was identified as an interactor of DPPA2 by our mass spectrometry in NCI-H661 cells (Fig. 2C) and mouse ESCs (Hernandez et al. 2018; Eckersley-Maslin et al. 2020). Consistently, KDM2A binding is enriched at DPPA2/4 peaks and correlates with DPPA2/4 enrichment at unmethylated CpG islands (Fig. 3D–F; Supplemental Fig. S3G). This colocalization is conserved in mouse ESCs (Supplemental Fig. S3H,I), suggesting that KDM2A may target DPPA2/4 to unmethylated CG-rich sequences, though further experiments are required to directly test this hypothesis.

To assess the chromatin context for DPPA2/4 binding, we profiled active (H3K4me3, H3K27ac, and H3K4me1), polycomb (H3K27me3 and H2AK119ub), repressive (H3K9me3), and transcribing (H3K36me2 and H3K36me3) histone modifications plus whole-genome bisulfite sequencing (WGBS) for genome-wide DNA methylation (Fig. 3G; Supplemental Fig. S3J,K; Supplemental Table S4) in the otherwise uncharacterized NCI-H661 cells. We first partitioned the genome into 200 bp bins and hierarchically clustered the signal for each mark against each other (Fig. 3H). DPPA2/4 were most highly correlated with active H3K4me3 and H3K27ac marks and, curiously, with PRC1-mediated H2AK119ub but not with PRC2-mediated H3K27me3 genome-wide (Fig. 3H) and at CpG islands (Supplemental Fig. S3L). To explore the chromatin states occupied by DPPA2/4 further, we computed a 17-state ChromHMM hidden Markov model (Ernst and Kellis 2012, 2017) that was able to capture informative combinations of epigenetic modifications (Supplemental Fig. S3M; Supplemental Table S6). Odds ratio testing of DPPA2/4 peak-overlapping ChromHMM states identified a preference for binding at active (H3K4me3, H3K27ac, and H2AK119ub) and bivalent (H3K4me3, H2AK119ub, and H3K27me3) TSS states as well as intragenic enhancers (H3K4me1 and H3K27ac) and transcriptional body (H3K36me3) chromatin states (Fig. 3I,J). Thus, DPPA2/4 co-occupancy across the genome occurs predominantly at active and poised CG-rich regulatory regions, similar to what is observed for murine DPPA2/4 in mouse embryonic stem cells (Hernandez et al. 2018; Klein et al. 2018; Eckersley-Maslin et al. 2020; Gretarsson and Hackett 2020).

While performing these analyses, we were particularly intrigued by the unusual distribution of H2AK119ub in NCI-H661 cells. H2AK119ub, which is deposited by the PRC1 complex, is generally considered to be found together with PRC2-deposited H3K27me3 at repressed gene loci (Laugesen et al. 2019; Uckelmann and Davidovich 2021), though there are several reports of H2AK119ub (H2AK118ub in *Drosophila*) occurring outside of H3K27me3 regions (Lee et al. 2015; Loubiere et al. 2020; Parmar et al. 2025). To eliminate any confounding effects of cellular or allelic heterogeneity in our bulk analyses and confirm true co-occurrence at these sites, we adapted our reChIP protocol (Seneviratne et al. 2024) to sequentially immunoprecipitate chromatin fragments containing both H3K4me3 and H2AK119ub. This confirmed that the two modifications did indeed co-occur on the same chromatin fragment (Fig. 3K,L; Supplemental Fig. S3N; Supplemental Table S4), including at regions devoid of H3K27me3 (Fig. 3m). Notably, DPPA2/4 were bound at 66.9% (*n* = 3132 out of 4684) of these H3K4me3–H2AK119ub regions (Fig. 3m), suggesting that they may have a role in facilitating this H2AK119ub–H3K4me3 chromatin structure.

### Loss of DPPA2/4 leads to H2AK119ub depletion at H2AK119ub–H3K4me3 promoters

**Figure 4.**
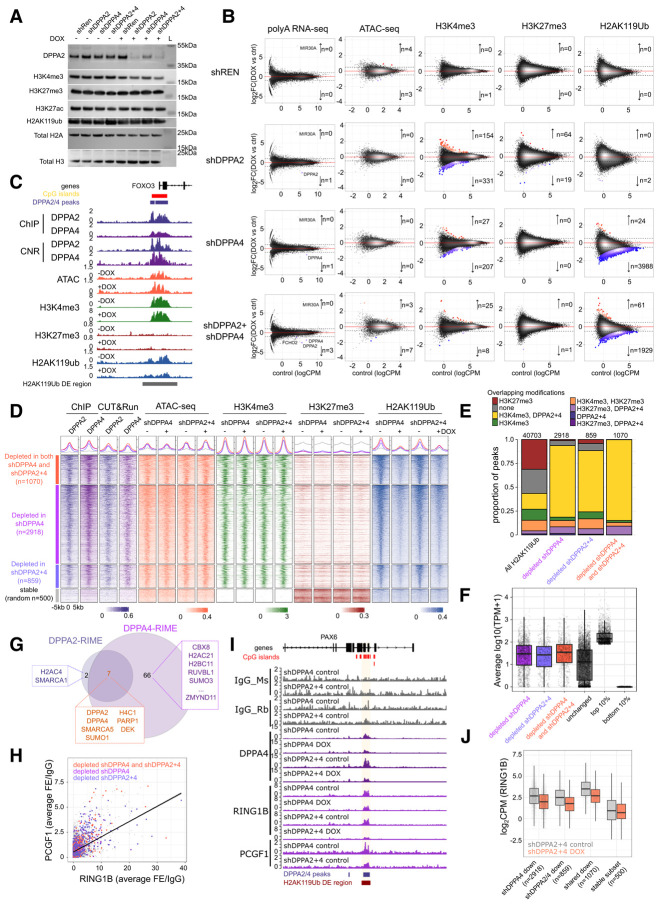
Loss of DPPA2/4 leads to H2AK119ub depletion at H2AK119ub–H3K4me3 promoters. (*A*) Representative Western blot of chromatin extracts from stable inducible NCI-H661 cell lines following 7 day shRNA-mediated knockdown of DPPA2 and/or DPPA4 (induction with a vehicle control [H_2_O] or 2 µg/mL doxycycline [DOX]). Uninduced and induced conditions are provided per cell line. (shREN) Nontargeting control, (shDPPA2) DPPA2 single knockdown, (shDPPA4) DPPA4 single knockdown, (shDPPA2+4) DPPA2+4 double knockdown. Blots show anti-DPPA2, anti-H3K4me3, anti-H3K27me3, anti-H3K27ac, anti-H2AK119ub, anti-H2A, or anti-H3. “L” indicates a molecular ladder; sizes are annotated at the *right*. (*B*) Scatter plots of differential RNA-seq and consensus (union of overlapping peaks in two replicates per condition) ATAC-seq, H3K4me3 ChIP-seq, H3K27me3 ChIP-seq, and H2AK119ub ChIP-seq peaks (columns) for the four stable inducible cell lines (shREN control [*top* row], shDPPA2 [second row], shDPPA4 [third row], and shDPPA2+shDPPA4 [*bottom* row]) comparing 7 day shRNA induction (doxycycline [DOX]) with vehicle control (H_2_O). Differentially expressed genes (RNA-seq, FDR <0.05, absolute log_2_FC ≥1, edgeR-TMM) or peaks (ATAC-seq, ChIP-seq FDR <0.05, absolute log_2_FC ≥0.5, edgeR-TMM) are shown in red for enriched/upregulated genes and in blue for depleted/downregulated genes. ATAC-seq and ChIP-seq data were normalized using edgeR-TMM against large 10 kb genomic bins genome-wide. The *Y*-axes indicate log_2_FC for DOX versus H_2_O, and the *X*-axes indicate log counts per million (logCPM) in the H_2_O controls. All differential tests represent testing between three (RNA-seq) or two (ATAC-seq and ChIP-seq) biological replicates per condition. RNA-seq plots also denote miR30A, which is part of the shRNA hairpin. (*C*) Genome view of the chromosome 6: 108,541,111–108,580,815 (hg38) locus. Gene annotation track, CpG islands (red), and DPPA2/4 peaks (purple) are shown *above*. Tracks show averaged DPPA2 (*n* = 2) and DPPA4 (*n* = 2) ChIPs and DPPA2 (*n* = 1) and DPPA4 (*n* = 1) CUT&RUN. The *bottom* eight tracks show averaged ATAC-seq, H3K4me3 ChIP-seq, H3K27me3 ChIP-seq, and H2AK119ub ChIP-seq for control versus DOX-induced shDPPA2+shRPPA4 cells (*n* = 2 each for H_2_O and DOX). Differential H2AK119ub peaks are depicted *below*. All scales are in counts per million per base pair. (*D*) Heat maps of genomic regions centered on depleted H2AK119ub peaks (*top* three row groups;depleted in both shDPPA4 and shDPPA2+4 [peach; *n* = 1070], depleted in shDPPA4 [purple; *n* = 2918], and depleted in shDPPA2+4 [blue; *n* = 859]) versus stable random regions (*bottom* group of rows; black; *n* = 500). Regions have been resized to ±5 kb from the center of each peak and split into 100 equal windows. Columns represent average DPPA2 (*n* = 2) and DPPA4 (*n* = 2) ChIPs and DPPA2 (*n* = 1) and DPPA4 (*n* = 1) CUT&RUN in parental cells (scaled counts per million per base pair values across all regions for visualization) and averaged ATAC-seq, H3K4me3, H3K27me3, and H2AK119ub ChIP-seq for shDPPA4 and shDPPA2+4 (*n* = 2 each for H_2_O and DOX; unscaled counts per million per base pair). (*E*) Proportional bar plot of overlapping histone modifications (H3K4me3 and H3K27me3) and/or DPPA2+4 binding in all (*left* column) or subsets of H2AK119ub regions depleted in shDPPA4 (second column), shDPPA2+4 (third column), or both shDPPA4 and shDPPA2+4 (*right* column). Total number of peaks per category are annotated *above* each bar. (*F*) Box plots of averaged RNA-seq data from parental NCI-H661 cells (*n* = 3) across different categories of H2AK119ub-marked promoters as well as the top 10% and bottom 10% of all expressed genes as controls. Units are in transcripts per million (log_10_TPM+1). (*G*) Venn diagram showing the overlap of DPPA2 RIME and DPPA4 RIME hits. (*H*) Scatter plot showing average fold enrichment over IgG for RING1B (*X*-axis) and PCGF1 (*Y*-axis) over 200 bp bins genome-wide. Highlighted are regions overlapping H2AK119ub peaks that are depleted in shDPPA4 (purple), shDPPA2+4 (blue), or both shDPPA4 and shDPPA2+4 (orange). (*I*) Genome view of the chromosome 11: 31,686,162–31,937,375 (hg38) locus. Gene annotation track and CpG islands (red) are shown *above*, and DPPA2/4 peaks (purple) and H2AK119ub-depleted peaks (red) are shown *below*. Tracks show individual mouse IgG (*n* = 1), rabbit IgG (*n* = 1), DPPA4 (*n* = 1), RING1B (*n* = 1), and PCGF1 (*n* = 1) CUT&RUN for control versus DOX-induced shDPPA4 or shDPPA2+shDPPA4 cells. All scales are in counts per million per base pair. (*J*) Box plots of RING1B enrichment in counts per million (log_2_CPM) between control (gray) and DOX-treated (orange) shDPPA2+4 cells at H2AK119ub peaks downregulated in shDPPA4 and/or DPPA2+4 compared with a subset of stable H2AK119ub peaks.

To test the impact of depleting DPPA2/4 on chromatin experimentally, we established both siRNA and inducible shRNA knockdown models for either DPPA2, DPPA4, or both DPPA2/4 in the NCI-H661 cell line (Supplemental Fig. S4A–C). This enables the more immediate effects of DPPA2/4 loss to be assessed while avoiding the potential confounding clonal effects observed with CRISPR knockout lines (Bernhard et al. 2022; Westermann et al. 2022). Similar to above, DPPA4 depletion by shRNA resulted in reduced DPPA2 protein levels (Fig. 4A), consistent with the requirement for DPPA4 for a stable functional complex.

We next profiled how DPPA2/4 loss affects transcription and chromatin states in NCI-H661 cells. There were essentially no transcriptional changes observed following DPPA2/4 depletion by shRNA (Fig. 4B) or siRNA (Supplemental Fig. S4D–F; Supplemental Table S7). Consistently, siRNA treatment in other cancer cell lines expressing just DPPA2 (A427 non-small cell lung cancer cells, DMS114 small cell lung cancer cells, and HuTu80 duodenal adenocarcinoma cells) or DPPA4 (COV318 ovarian cancer cells) had minor effects on transcription (Supplemental Fig. S4G–J). Epigenetic priming factors, such as DPPA2/4, often do not directly regulate gene transcription but rather facilitate poised chromatin states (Eckersley-Maslin 2020). We therefore assessed epigenomic marks that DPPA2/4 are known to regulate in mESCs (Eckersley-Maslin et al. 2020; Gretarsson and Hackett 2020). Surprisingly, there were also no changes in chromatin accessibility (ATAC-seq) or canonical bivalent histone modifications H3K4me3 and H3K27me3 following DPPA2/4 shRNA depletion (Fig. 4B; Supplemental Table S7). Furthermore, DNA methylation landscapes remained largely unchanged when DPPA2/4 were depleted individually or concurrently by siRNAs in NCI-H661 cells (Supplemental Fig. S4K–M) or following DPPA4 siRNA treatment in COV318 cells (Supplemental Fig. S4N).

Notably, we observed a substantial loss of the PRC1-deposited H2AK119ub modification in the DPPA4 single-knockdown and DPPA2/4 double-knockdown cells (Fig. 4B,C; Supplemental Table S7). Of the 3182 DPPA2/4-bound H3K4me3–H2AK119ub reChIP peaks, 47.5% (*n* = 1511) and 21.2% (*n* = 676) were depleted in shDPPA4- and shDPPA2+4-treated cells, respectively, likely due to technical differences in statistical thresholding between conditions. This was not associated with any changes in overall H2AK119ub levels (Fig. 4A), arguing for locus-specific loss rather than global depletion of the modification. Closer examination of the H2AK119ub-depleted regions revealed that these were co-occupied by DPPA2/4 and H3K4me3 but lacked H3K27me3 (Fig. 4D,E). The H2AK119ub-depleted regions were largely associated with gene promoters associated with developmental processes, Wnt signaling, and morphogenesis (Supplemental Fig. S4O–Q; Supplemental Table S8) that were robustly expressed (Fig. 4F).

We next sought to further understand how DPPA2/4 may be regulating H2AK119ub at these active domains. As our endogenous mass spectrometry did not detect any direct interactions between DPPA2/4 and PRC1 complex members (Fig. 2C; Supplemental Fig. S2L,M), we performed RIME (Mohammed et al. 2016) to determine whether more transient interactions may be taking place. This involved double-cross-linking chromatin prior to immunoprecipitation with either DPPA2/4 antibodies or isotype-matched IgG controls and mass spectrometry. We performed RIME in wild-type cells (*n* = 2 clones) and included CRISPR double-knockout cells (*n* = 2 clones) as negative controls. Following stringent filtering, we identified 75 proteins associated with DPPA2 and/or DPPA4 across both wild-type clones (Fig. 4G; Supplemental Fig. S4R–U; Supplemental Table S9). Importantly, these included DPPA2 and DPPA4 themselves, alongside the chromatin remodeler SMARCA5, which had been shown previously to biochemically and functionally interact with DPPA2/4 in mESCs (Alda-Catalinas et al. 2020; Eckersley-Maslin et al. 2020); the nucleosome binding protein DEK, which has been shown recently to stimulate PRC2 activity (Kujirai et al. 2025); and SUMO1, which has been shown previously to interact with and regulate DPPA2/4 levels in mESCs (Theurillat et al. 2020). Many other proteins that interact with DPPA2 and/or DPPA4 were histone proteins, chromatin remodelers, DNA replication and repair components, and proteins involved in transcription and RNA processing (Supplemental Table S9). Of note, the DPPA4 RIME detected CBX8 (a component of the canonical PRC1 complex that can recognize H3K27me3 via its chromodomain) (Connelly et al. 2019) but no other core or accessory subunits of PRC1 complexes. Interestingly, in addition to silencing roles (Suh et al. 2022), CBX8 has been linked with facilitating the activation of Polycomb-repressed genes during mESC differentiation (Creppe et al. 2014). It remains to be determined whether CBX8 has similar roles in NSCLC cells.

To further interrogate PRC1 binding and activity at DPPA2/4 domains, we profiled by CUT&RUN RING1B, the core catalytic subunit present in all PRC1 complexes, alongside PCGF1, a component of variant PRC1, which was shown previously to interact with DPPA4 in human NT2 teratocarcinoma cells (Oliviero et al. 2016). As expected, both RING1B and PCGF1 were enriched at the DPPA2/4-dependent H2AK119ub regions (Fig. 4G,H), confirming the presence of PRC1 at these domains in WT cells. Moreover, following DPPA2/4 depletion, RING1B was reduced at the DPPA2/4-dependent H2AK119ub domains (Fig. 4H,I), though this was less pronounced in DPPA4-depleted cells (Supplemental Fig. S4U), potentially due to residual levels of DPPA2 protein (Fig. 4A). Together, these results suggest that DPPA2/4 facilitate RING1B binding and H2AK119ub deposition at active H3K4me3 regions devoid of H3K27me3 in NSCLC cells.

### H3K27ac at H2AK119ub–H3K4me3 promoters facilitates high expression

**Figure 5.**
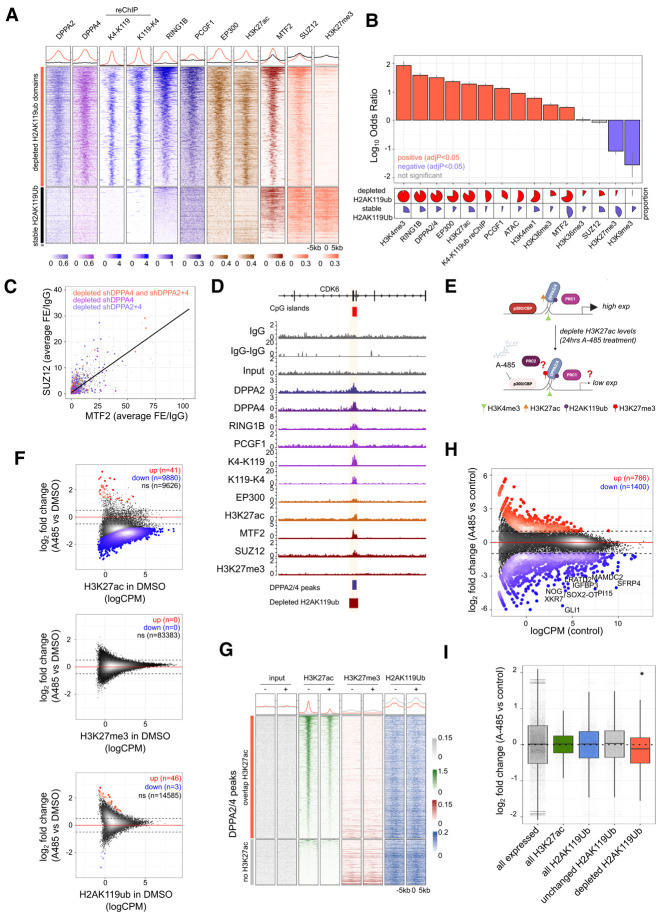
H3K27ac at H3K4me3–H2AK119ub promoters facilitate high expression. (*A*) Heat maps of genomic regions centered on H2AK119ub-depleted (*top*) versus stable (*bottom*) peaks (resized to ±5 kb from the center of each peak, split into 100 equal windows). Columns depict average DPPA2 (*n* = 2) and DPPA4 (*n* = 2) ChIPs; H3K4me3–H2AK119ub (*n* = 2) and H2AK119ub–H3K4me3 (*n* = 2) reChIPs; and average RING1B (*n* = 2), PCGF1 (*n* = 2), EP300 (*n* = 2), H3K27ac (*n* = 2), MTF2 (*n* = 2), SUZ12 (*n* = 1), and H3K27me3 (*n* = 2) ChIPs in parental cells. Values were scaled per bin across all regions for visualization. (*B*) Odds ratio tests of all H2AK119ub-depleted regions compared with unchanged H2AK119ub regions. States are ordered by decreasing odds ratio, reflecting enriched overlaps (log_10_ odds ratio >0) compared with depleted overlaps (log_10_ odds ratio <0). Error bars represent the upper and lower 95% confidence intervals for hazard ratios, and colored overlaps have a BH-adjusted -value of <0.05. Pie charts represent the proportion of peaks overlapping each mark. (*C*) Scatter plot showing average fold enrichment over IgG for MTF2 (*X*-axis) and SUZ12 (*Y*-axis) over 200 bp bins genome-wide. Highlighted are regions overlapping H2AK119ub peaks that are depleted in shDPPA4 (purple), shDPPA2+4 (blue), or both shDPPA4 and shDPPA2+4 (orange). (*D*) Genome view of the chromosome 7: 92,758,743–92,906,906(hg38) locus. Gene annotation track and CpG islands (red) are shown *above*. Tracks (*top* to *bottom*) show IgG (*n* = 1); averaged IgG–IgG reChIP (*n* = 2); input ChIP control (*n* = 1); and average DPPA2 (*n* = 2), DPPA4 (*n* = 2), RING1B (*n* = 2), PCGF1 (*n* = 2), H2AK119ub–H3K4me3 (*n* = 2), H3K4me3–H2AK119ub (*n* = 2), EP300 (*n* = 2), H3K27ac (*n* = 2), MTF2 (*n* = 2), SUZ12 (*n* = 1), and H3K27me3 (*n* = 2) ChIPs/reChIPs. Scales are in counts per million per base pair. DPPA2/4 peaks (blue) and depleted H2AK119ub peaks (red) are denoted *below*. (*E*) Schematic of A-485 experiment. (*F*) Scatter plots of H3K27ac (*top*), H3K27me3 (*middle*), and H2AK119ub (*bottom*) ChIPs showing peak enrichment in DMSO control (*X*-axis) compared with log_2_ fold change (A485 vs. DMSO; *Y*-axis). Statistically enriched (red) and depleted (blue) peaks are highlighted (FDR <0.05, absolute log_2_FC >0.5, edgeR-TMM). (*G*) Heat maps of genomic regions centered on DPPA2/4 peaks (resized to ±5 kb from peak center, split into 100 equal windows) overlapping H2AK119ub-depleted regions (*top*) compared with those that had unchanged H2AK119ub (*bottom*) comparing DMSO control (−) versus A-485-treated (+) cells. Columns show input (gray), H3K27ac (green), H3K27me3 (red), and H2AK119ub (blue). Values were scaled per bin across all regions for visualization. (*H*) Scatter plot of RNA-seq data in NCI-H661 cells following treatment with either a vehicle control (DMSO) or 10 µM A-485 for 24 h (*n* = 3 biological replicates). Differentially expressed genes are highlighted (absolute log_2_FC ≥1 and FDR <0.05, edgeR-TMM). The *Y*-axis represents the log_2_FC between A-485 and DMSO control, and the *X*-axis indicates the log counts per million (logCPM) in the DMSO control. (*I*) Box plots of gene expression changes (log_2_FC) upon A-485 treatment relative to DMSO control across different gene categories: all expressed genes (black; *n* = 29,244), all H3K27ac-only gene promoters (green; *n* = 3795), all H2AK119ub gene promoters (gray; *n* = 6540), unchanged H2AK119ub gene promoters (gray; *n* = 5877), and commonly depleted H2AK119ub gene promoters in shDPPA4 and shDPPA2+4 (red; *n* = 699). Boxes indicate the interquartile ranges, and error bars indicate the upper and lower 95% confidence intervals of all values.

The absence of H3K27me3 at these H2AK119ub–H3K4me3 promoters was of particular interest given the positive feedback loops between PRC1 and PRC2 (Laugesen et al. 2019; Uckelmann and Davidovich 2021). Several mechanisms could explain this discordance: PRC2 may not be present at the H3K4me3–H2AK119ub domains or may be catalytically inactive and/or unable to methylate its substrate. Alternatively, any H3K27me3 may be rapidly removed by demethylases. To test whether PRC2 is effectively recruited, we generated ChIP-seq data for SUZ12 (Fig. 5A; Supplemental Fig. S5A–C), a core component of PRC2 that helps stabilize the complex (Glancy et al. 2021), alongside CUT&RUN data for MTF2, which recruits PRC2.1 to unmethylated CpG islands (Perino et al. 2018) and was detected as an interactor of DPPA2 in our mass spectrometry experiments (Fig. 2C). Notably, the PRC2.1 complex member MTF2 was robustly bound across all stable and depleted H2AK119ub domains (Fig. 5A,B; Supplemental Table S10). Supporting this, there were low but detectable levels of SUZ12 at the H2AK119ub-depleted domains (Fig. 5A; Supplemental Fig. S5B). While only a minor fraction of regions overlapped SUZ12 peaks (Fig. 5B), this could be due to the lower enrichment at most regions that did not pass peak calling thresholds (Fig. 5C), as MTF2 and SUZ12 typically colocalize at target genes (Supplemental Fig. S5B; Healy et al. 2019; Højfeldt et al. 2019). This suggests that PRC2.1 is present at these active domains but does not result in detectable levels of H3K27me3.

One potential explanation for this discrepancy was the presence of H3K27ac at these H2AK119ub–H3K4me3 domains (Fig. 3J). We therefore profiled binding of the H3K27ac histone acetyltransferase EP300 and integrated this with our previous H3K27ac ChIP-seq. This revealed high levels of both EP300 and H3K27ac at these DPPA2/4-dependent H2AK119ub domains (Fig. 5A,B,D). We hypothesized that the presence of H3K27ac at these DPPA2/4-dependent H2AK119ub domains may be hampering PRC2 activity at these sites. To test this, we treated NCI-H661 cells with A-485, a potent inhibitor of p300/CBP activity (Fig. 5E; Lasko et al. 2017). As expected, 24 h of A-485 treatment resulted in reduced H3K27ac levels at thousands of regions across the genome, while H2AK119ub levels remained largely unchanged (Fig. 5F,G; Supplemental Fig. S5C; Supplemental Table S11). Notably, we did not see any changes in H3K27me3 with A-485 treatment across the genome (Fig. 5F). Moreover, at DPPA2/4-bound regions, there was no detectable H3K27me3, independent of H3K27ac status (Fig. 5G; Supplemental Fig. S5C). These results suggest that H3K27ac is not actively preventing PRC2 activity at these H2AK119ub–H3K4me3 domains.

To understand the transcriptional consequences of depleting H3K27ac at these regions, we performed RNA sequencing analysis on the A-485-treated cells. Consistent with the broad effects of p300/CBP inhibitors, we observed widespread transcriptional deregulation with 786 upregulated and 1400 downregulated genes (Fig. 5H; Supplemental Table S11). Importantly, those H2AK119ub-enriched promoters that were sensitive to DPPA2/4 loss were repressed upon A-485 treatment (Fig. 5I; Supplemental Fig. S5D), with 318 downregulated genes (22.7% of all downregulated genes) also linked to H2AK119ub-depleted domains. This suggests that H2AK119ub at these regions can repress genes in the absence of H3K27ac through H3K27me3-independent mechanisms.

### DPPA2/4 amplify active chromatin landscapes in non-small cell lung cancer cells

**Figure 6.**
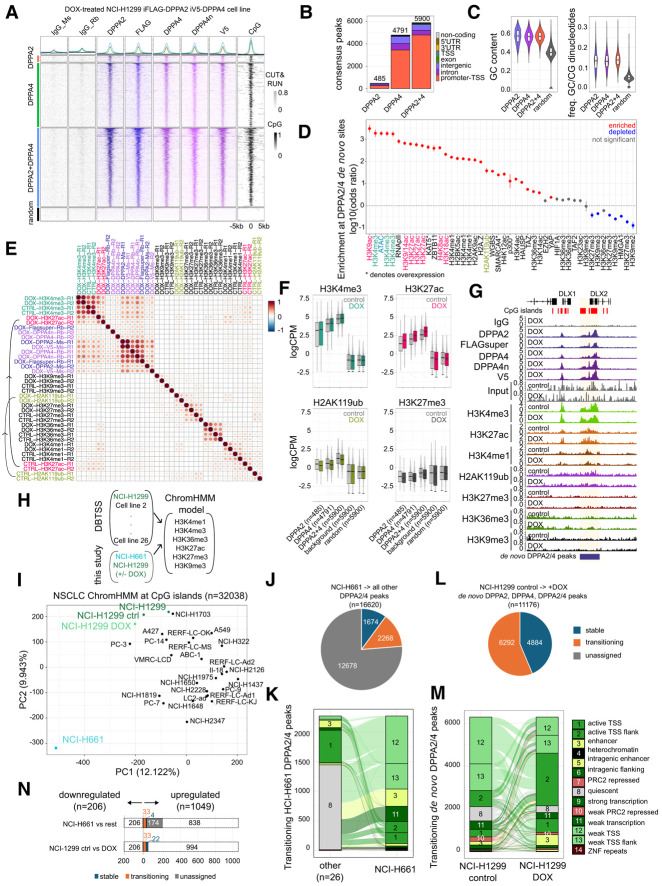
DPPA2/4 amplify active chromatin landscapes in non-small cell lung cancer cells. (*A*) Heat maps of genomic regions centered on de novo DPPA2/4 peaks in HCI-H1299 cells (resized to ±5 kb from peak center, split into 100 equal windows) comparing DPPA2 (*top*), DPPA4 (second row), and DPPA2+DPPA4 (third row) peaks with a random set of genomic regions (*bottom*). Columns show IgG controls (gray), DPPA2 (purple; endogenous or FLAG antibodies; *n* = 2 each), and DPPA4 (pink; two separate endogenous antibodies or V5 antibody; *n* = 2 each). CpG density is shown in black. Values were scaled per bin across all regions for visualization. (*B*) Genomic annotations of de novo DPPA2+4 consensus peaks. The promoter TSS is defined as the TSS ± 1500 bp. (*C*) G/C content of (*left*) and GC/CG dinucleotide fraction within (*right*) de novo DPPA2+4 consensus peaks compared with size-matched random genomic regions. (*D*) Enrichment of de novo peaks in NCI-H1299 cells with different histone modifications, transcription factors, and epigenetic marks. Enriched factors are shown in red, and depleted factors are shown in blue. Whole-genome bisulfite sequencing (WGBS) represents enrichment of hypomethylated regions. Factors are ranked by log_10_ odds ratio. An asterisk denotes that the data set was derived from an overexpression model. (*E*) Genome-wide pairwise correlations of ChIP-seq (DPPA2, DPPA4, H3K4me3, H3K27ac, H3K4me1, H3K27me3, H2AK119ub, H3K9me3, H3K36me2, and H3K36me3 fold enrichment over the input control) between control (CTRL) and DOX-treated conditions in NCI-H1299 overexpression models, partitioned into 200 bp bins genome-wide. Dots are colored and sized by Pearson correlation coefficients (*r*) and ordered by hierarchical clustering. (*F*) Box plots showing H3K4me3 (*top left*), H3K27ac (*top right*), H2AK119ub (*bottom left*), or H3K27me3 (*bottom right*) enrichment (logCPM) between control (gray) and DOX-treated (colored) HCI-1299 cells at DPPA2 and/or DPPA4 peaks compared with background or random set of regions. (*G*) Genome view of the chromosome 2: 172,067,177–172,123,476 (hg38) locus. Gene annotation track and CpG islands (red) are shown *above*. Tracks (*top* to *bottom*) show IgG (*n* = 1), DPPA2, FLAGSuper, DPPA4, DPPA4n, and V5 CUT&RUN samples in DOX-treated NCI-H1299 cells, followed by input, H3K4me3, H3K27ac, H3K4me1, H2AK119ub, H3K27me3, H3K36me3, and H3K9me3 ChIP-seq comparing control (*n* = 2 each) and DOX-treated (*n* = 2 each) cells. De novo peaks are shown *below* in blue. Scales are in counts per million per base pair. (*H*) Schematic of data integration between NCI-H661 (this study), control and DOX-treated NCI-H1299 (this study), and DBTSS (*n* = 26 NSCLC cell lines [Suzuki et al. 2015]) cells to generate a joint 14-state ChromHMM model of all cell lines. (*I*) Principal component analysis (PCA) of factorized chromatin states (ChromHMM) at CpG islands (*n* = 32,038) across all NSCLC cell lines (*n* = 27). (*J*) The proportion of DPPA2/4 peaks defined in NCI-H661 cells that overlap stable (blue) and transitioning (orange) states between NCI-H661 cells and all other cell lines (Suzuki et al. 2015). Those in which peaks could not be assigned a consistent state in >75% of other lines are denoted as unassigned. (*K*) Alluvial plot of transitioning chromatin states (DPPA2/4 peaks that differ in chromatin state between NCI-H661 cells and all other NSCLC cell lines and are present in at least 75% of other NSCLC lines). The ribbons between the bars indicate how chromatin states transition between NCI-H661 cells and the majority of other NSCLC cell lines. (*L*) The proportion of de novo DPPA2/4 peaks defined in NCI-H1299 cells that overlap stable (blue) and transitioning (orange) states between control and DOX-treated NCI-H1299 cells. (*M*) Alluvial plot of transitioning chromatin states at de novo DPPA2/4 peaks between control and DOX-treated NCI-H1299 cells. The ribbons between the bars indicate how chromatin states transition between control and DOX conditions. (*N*) Bar plots showing the total number of differentially expressed genes in TCGA (white bars), highlighting those that overlap DPPA2/4 peaks that are stable (blue) or transitioning (orange) in either NCI-H661 versus rest (*top*) or NCI-H1299 control versus DOX (*bottom*) comparisons.

So far, our results support a model in which DPPA2/4 binding at active regions acts as an amplifier, enabling chromatin complexes, remodelers, and/or transcription factors to more readily access these domains. To determine whether DPPA2/4 similarly facilitate a more active chromatin state in other non-small cell lung cancer cell lines, we turned to the NCI-H1299 overexpression model we had initially used in our xenograft models (Fig. 1H,I). These cells contain exogenous DOX-inducible FLAG-DPPA2 and V5-DPPA4 transgenes, enabling robust de novo expression of both proteins in an otherwise DPPA2/4-naive context. We performed CUT&RUN using DPPA2, DPPA4 (two separate antibodies), FLAG, and V5 antibodies to profile where DPPA2/4 bind when introduced into cells that do not normally express these proteins. As expected, the DPPA2 and DPPA4 binding profiles largely overlapped (Fig. 6A; Supplemental Fig. S6A–C), with 5900 de novo shared peaks occurring predominantly at CpG-rich promoter regions (Fig. 6B,C; Supplemental Fig. S6D; Supplemental Table S12). To understand the chromatin context of DPPA2/4 binding, we reprocessed previously published ATAC-seq, WGBS, and ChIP-seq data sets performed in NCI-H1299 cells (Suzuki et al. 2015; García-Carpizo et al. 2016; Wu et al. 2016; Brocks et al. 2017; Hsu et al. 2018; Zhang et al. 2018, 2024; Jang et al. 2019; Shreberk-Shaked et al. 2020; Romero et al. 2021; Karlow et al. 2022; Cheng et al. 2024; Olan et al. 2024; Xu et al. 2024; Xuan et al. 2024) and analyzed which epigenomic features were enriched at the de novo DPPA2/4 sites. This revealed a strong and statistically significant preference for DPPA2/4 de novo binding at open chromatin marked by H3K4me3, RNA polymerase II, histone acetylation (H3K9/14ac, H3K27ac, H4K8ac, and H3K18ac), and, to a lesser extent, H2AK119ub (Fig. 6D). These results are consistent with our observations in NCI-H661 cells where endogenously expressed DPPA2/4 bind active open chromatin regions (Fig. 3I,J).

To understand the impact that DPPA2/4 recruitment to these regions may have on their chromatin states, we performed ChIP-seq for H3K4me1, H3K4me3, H3K9me3, H3K27me3, H3K36me3, and H3K27ac in control versus DOX-treated NCI-H1299 cells (Supplemental Fig. S6E). We first partitioned the genome into 200 bp bins and formed pairwise Pearson correlations between each of the different histone modifications together with the DPPA2/4 CUT&RUN data (Fig. 6E). As above, de novo DPPA2/4 binding correlated with active H3K4me3 but not the more repressive H3K27me3 or H3K9me3. Interestingly, overexpression of DPPA2/4 resulted in an increased correlation between H3K27ac and H3K4me3 genome-wide (Fig. 6E). Furthermore, H3K27ac levels were significantly increased at DPPA2/4 de novo sites (Fig. 6F), while other histone modifications were largely unchanged (Fig. 6F,G; Supplemental Fig. S6E–H). In summary, when DPPA2/4 are exogenously expressed, they predominantly cobind active CpG-rich promoters to enhance levels of H3K27ac at these sites.

Last, we sought to understand more broadly the molecular consequences of DPPA2/4 expression across NSCLC cells. We combined our chromatin maps in NCI-H661 and NCI-H1299 cells together with a comprehensive data set profiling the same six chromatin marks across 26 NSCLC cell lines (Suzuki et al. 2015) that do not normally express DPPA2/4 (Supplemental Fig. S6I) and generated an integrated 14-state model (Supplemental Fig. S6J). Importantly, NCI-H1299 cells were also profiled in this external study, enabling us to control for technical differences between studies. When focusing on CpG islands (Fig. 6I) or DPPA2/4 binding sites in NCI-H661 cells (Supplemental Fig. S6K), we noted that NCI-H661 cells clustered away from all other cell lines. Moreover, there was a modest shift in NCI-H1299 cells that had been treated with DOX toward the DPPA2/4-expressing NCI-H661 cells (Supplemental Fig. S6L). This was not observed when a size-matched control set of random genomic regions was analyzed, arguing against technical or batch differences confounding our analysis (Supplemental Fig. S6M). We then labeled each of the DPPA2/4-bound sites by the most prevalent ChromHMM state in each cell line. For further analysis, we focused on those that were the same in at least 75% of all non-NCI-H661 cell lines (*n* = 3942; all other regions were denoted as unassigned). “Stable” states (*n* = 1674) were defined as those prevalent states that remained the same in NCI-H661 cells, while “transitioning” states (*n* = 2268) changed their chromatin state in NCI-H661 cells compared with other NSCLC lines (Supplemental Table S13). Notably, the largest set of transitioning domains shifted from quiescent to active TSSs and enhancer states in NCI-H661 cells (Fig. 6K). Similarly, in the NCI-H1299 overexpression model, DPPA2/4 rewired the chromatin state at 56% (*n* = 6292) of its de novo binding sites predominantly toward more active states (Fig. 6L,M; Supplemental Table S13). This supports the role of DPPA2/4 in amplifying active chromatin states.

Last, we sought to understand the potential consequences of DPPA2/4 binding and amplification of chromatin states on tumors. We assigned each stable or transitioning domain to the closest gene and compared the overlap with the differentially expressed genes in NSCLC tumors with DPPA2/4 coexpression (Fig. 1J). Notably, none of the 206 downregulated genes in DPPA2/4-coexpressing tumors were associated with DPPA2/4 binding. Instead, DPPA2/4-bound regions were only associated with upregulated genes in either endogenous or exogenous contexts (Fig. 6n). In particular, among those genes linked to DPPA2/4-bound and transitioning domains were drivers of lung cancer pathogenesis and/or metastasis, including VGF (Hwang et al. 2017; Marwitz et al. 2017; Yang et al. 2022) and SALL4 (Du et al. 2018; Jiang and Liu 2018; Li et al. 2020). Together, our findings suggest that DPPA2/4 binding at these regions may be enhancing chromatin states to promote expression of linked genes to facilitate the detrimental outcomes seen in patients with DPPA2/4-expressing cancers.

## Discussion

In this study, we uncovered the molecular impact of coexpressing the embryonic priming factors DPPA2/4 in cancer cells. Our comprehensive pan-cancer analysis revealed how upregulation of DPPA2/4, likely through promoter DNA demethylation, is associated with poorly differentiated phenotypes and worse outcomes, particularly for NSCLC patients. DPPA2/4 dimerize for their stability and nucleosome binding at CpG-rich promoters and enhancers and are required to maintain PRC1 and H2AK119ub at these regulatory regions that are largely devoid of PRC2-mediated H3K27me3. Instead, high levels of H3K27ac at these domains promote transcription. Overexpressing DPPA2/4 in NSCLC cells that do not normally contain these proteins revealed that DPPA2/4 bind to and promote active chromatin states. Our results support a revised model for DPPA2/4 as amplifiers of active/poised chromatin landscapes, potentially by facilitating the stabilization and/or residence time of chromatin complexes at these domains. In this model, the precise molecular impact of DPPA2/4 expression depends on the cellular context: While DPPA2/4 function in mESCs to amplify the canonical H3K4me3–H3K27me3 bivalent state (Eckersley-Maslin et al. 2020; Gretarsson and Hackett 2020), in our NSCLC models, DPPA2/4 instead amplify a H2AK119ub–H3K4me3–H3K27ac state. However, as the majority of our experimental profiling was performed in just two NSCLC cell lines, it will be important in future studies to determine how generalizable the role of DPPA2/4 in amplifying chromatin states is in other contexts.

DPPA2/4 are not robustly expressed outside of early development, prompting our investigation of their functions when upregulated in poorly differentiated cancers. There have been numerous reports of DPPA2 or DPPA4 expression in a range of tumor types (John et al. 2008; Tchabo et al. 2009; Deb et al. 2010; Amini et al. 2014; Raeisossadati et al. 2014; Ghodsi et al. 2015; Zhang et al. 2015; Shabestarian et al. 2016); however, the majority of these studies assessed the proteins in isolation and/or had limited mechanistic follow-up investigations. Our systematic pan-cancer analysis revealed that coexpression of DPPA2/4 is associated with impaired outcomes and dedifferentiated phenotypes in NSCLC. Consistently, coexpression of DPPA2/4 in NSCLC xenograft models results in a modest increase in tumor growth in vivo. Similarly, DPPA2 depletion in testicular germ cell tumors impairs tumor growth (Wang et al. 2021), and DPPA4 has been shown to transform fibroblasts (Tung et al. 2013).

Our biochemical investigation uncovered a stability axis between DPPA2 and DPPA4. On its own, DPPA2 protein is unstable in cells and requires DPPA4 for its stability. Our RIME proteomics uncovered an interaction between DPPA2/4 and SUMO1, which in mESCs has been shown to regulate DPPA2/4 proteins (Theurillat et al. 2020). Additionally, DPPA2 contains a SPOP-binding consensus degron that can be degraded by the SPOP–CUL3–RBX1 E3 ubiquitin–ligase complex (Wang et al. 2021), which is expressed in our NSCLC models. It is possible that degradation via SUMO and/or SPOP is prevented when DPPA4 is present in the cells as the two proteins multimerize. Consistently, despite a direct interaction between SPOP and DPPA2 in mESCs, depleting SPOP has no effect on DPPA2 protein levels (Gupta et al. 2023), potentially due to a stabilizing effect from DPPA4. In contrast, DPPA4 can multimerize and bind nucleosomes independently of DPPA2, suggesting potential divergence of function for human DPPA4 compared with mouse DPPA4.

DPPA2/4 have a genome-wide binding distribution in human cancer cells similar to what has been described in mouse embryonic stem cells (Hernandez et al. 2018; Klein et al. 2018; Gretarsson and Hackett 2020), suggesting that DPPA2/4 may co-opt stem cell-related functions in cancer cells. In NSCLC cells, we revealed a key role for DPPA2/4 in facilitating H2AK119ub at thousands of active H3K4me3 promoters and enhancers. H2AK119ub is catalyzed by Polycomb repressive complex 1 (PRC1) and is conventionally a repressive chromatin mark. It is well documented that H2AK119ub can recruit PRC2 to mediate H3K27me3 deposition, which in turn can recruit canonical PRC1 (Blackledge et al. 2014, 2020; Kalb et al. 2014; Cooper et al. 2016; Fursova et al. 2019; Healy et al. 2019; Tamburri et al. 2020; Kasinath et al. 2021; Sugishita et al. 2021; Ohtomo et al. 2023). Consequently, H2AK119ub and H3K27me3 typically co-occur to mediate repression; however, H2AK119ub and PRC1 have been shown in several other contexts to promote transcription (Frangini et al. 2013; Lee et al. 2015; Morey et al. 2015; Kloet et al. 2016; Loubiere et al. 2016, 2020; Pherson et al. 2017; Chan et al. 2018; Cohen et al. 2018; Zhao et al. 2024; Parmar et al. 2025). We revealed that this coupling between H2AK119ub and H3K27me3 is disrupted in NCI-H661 cancer cells, with H2AK119ub also co-occurring with active-associated H3K4me3 and H3K27ac modifications. This highlights the importance of studying H2AK119ub landscapes (which have not been profiled as extensively as H3K27me3) and not assuming coexistence between H2AK119ub and H3K27me3.

Excitingly, our findings point to an uncoupling between the recruitment of PRC2 to chromatin and its catalytic product. Many of the H2AK119ub regions that were dependent on DPPA2/4 were bound by the PRC2.1 member MTF2 but lacked detectable H3K27me3. This suggests that while PRC2 may be recruited to these H2AK119ub–H3K4me3 domains, its catalytic activity is blocked and/or the H3K27me3 modification is actively removed. Similar separation of function for p300/CBP has been described recently, whereby p300/CBP is physically present but lacks catalytic activity at repressed promoters (Hunt et al. 2022). This highlights the importance of assessing both recruitment and catalytic functions of chromatin regulators, as the two properties can be uncoupled and may be contributing to altered pathologies such as cancer.

Bivalent chromatin was first described almost two decades ago in mESCs (Azuara et al. 2006; Bernstein et al. 2006) and has since been reported in multiple contexts and organisms, including human cancers (Macrae et al. 2023; Glancy et al. 2024). It is traditionally defined as the co-occurrence of H3K27me3 and H3K4me3 on the same nucleosome or chromatin fragment; however, many other chromatin marks, including H2AK119ub, will also co-occur at these domains (Macrae et al. 2023). Our work highlights the importance of considering other combinations of seemingly opposing histone modifications as poised chromatin states. Cancer can serve as a useful sandbox for the discovery of many of these states, which may be harder to detect in other cellular contexts because they may be rare or transient in normal cells. Consequently, it will be important to understand how widespread these alternative poised states are in other cellular contexts.

## Materials and methods

See the Supplemental Material for detailed experimental and computational methods.

### Cell culture

Non-small cell lung cancer cell lines NCI-H661 and NCI-H1299 were cultured in RPMI-1640 (Gibco), A427 and HuTu80 with EMEM (Gibco), DMS114 with Waymouth (Gibco), and Cov318 with DMEM (Gibco) growth medium, all supplemented with 10% heat-inactivated fetal bovine serum (FBS) and 1× penicillin/streptomycin at 37°C and 5% CO_2_. E14 mouse embryonic stem cells were cultured on feeder-free gelatinized plates at 37°C and 5% CO_2_ using standard serum/LIF conditions: high-glucose DMEM supplemented with 15% FBS, 1× GlutaMAX, 1× penicillin/streptomycin, 0.1 mM nonessential amino acids, 50 mM β-mercaptoethanol, and LIF (made in-house in HEK293 cells and titrated for optimal ESC growth). Cells were routinely passaged to maintain exponential growth using Trypsin-Low EDTA (LE) Express (Gibco) to detach cells each time. Cells were regularly tested for mycoplasma contamination using the mycoplasma PCR detection kit (Abcam ab289834). NSCLC cell lines were also STR-profiled to ensure authenticity. NCI-H661 (XY), NCI-H1299 (XY), A427 (XY), HuTu80 (XY), DMS114 (XY), and Cov318 (XX) were purchased from ATCC, and E14 mouse embryonic stem cells (XY) were a gift from Wolf Reik's laboratory.

### Subcellular fractionation

After 7 days of induction 2 × 10^6^ freshly harvested NCI-H661 shRNA cells were washed once with ice-cold PBS. Cells were pelleted at 400*g* and washed three times in 200 µL of cytoplasmic extraction buffer (50 mM HEPES, 140 mM NaCl, 1 mM EDTA, 10% glycerol, 0.1% Igepal CA-630, 0.25% Triton X, 1 mM DTT in H_2_O) on ice, with the first wash being kept as the cytoplasmic fraction after 10 min of incubation. Nuclei were then washed three times in 80 µL of nuclear extraction buffer (10 mM Tris-HCl, 200 mM NaCl, 1 mM EDTA, 0.5 mM EGTA in H_2_O) on ice to release soluble nuclear proteins, the first wash of which was kept as the nuclear (soluble) fraction after 10 min of incubation. Insoluble chromatin was then resuspended in 200 µL of chromatin extraction buffer (50 mM Tris-HCl, 20 mM NaCl, 1 mM MgCl_2_, 0.1% SDS, 1:1000 benzonase [Merck E1014]) on ice and rotated for 20 min at room temperature, followed by a spin at 16,000*g* at 4°C to pellet debris, of which the supernatant was kept as the chromatin (insoluble) fraction. All extraction buffers were freshly supplemented with 1× protease inhibitor tablet (Roche). All intermediate spins between the first and last incubation were at 11,000*g* at 4°C. Successful cytoplasm–chromatin fractionation was confirmed by performing gel electrophoresis, transferring to nitrocellulose membranes, staining with Ponceau S, and observing the presence/absence of histones (H3, H2A/B, and H4) between 10 and 25 kDa in each fraction.

### Chromatin immunoprecipitation (ChIP)

For DPPA2, DPPA4, KDM2A, and SUZ12 ChIPs, 4 × 10^6^ NCI-H661 cells were utilized per ChIP and underwent double-cross-linking prior to chromatin isolation. All other ChIPs against histone modifications (H3K4me3, H3K4me1, H3K27ac, H3K27me3, H2AK119ub, H3K9me3, H3K36me3, and H3K36me2) utilized 5 × 10^5^ NCI-H661 or 1 × 10^6^ NCI-H1299 cells per ChIP and underwent single-cross-linking. Cells undergoing only single-cross-linking were resuspended in 9.375 mL of PBS at this point. For double-cross-linking, cells were harvested, washed once with PBS, and resuspended in 10 mL of PBS with 5 mM EDTA on ice. A fresh stock of 0.24 m disuccinimidyl glutarate (Thermo Fisher 20593) was reconstituted in DMSO and added to the suspension of cells to reach a final concentration of 2 mM, which was then incubated while rocking for 45 min at room temperature to cross-link cells. Cells were pelleted at 3200*g* for 5 min and resuspended in 9.375 mL of PBS with 5 mM EDTA. For both single- and double-cross-linked cells, 625 µL of fresh 16% formaldehyde (methanol-free; Thermo Fisher 28908) was added to each cell suspension to reach a final concentration of 1% and then incubated on a rocker for 12.5 min at room temperature to cross-link cells. Cross-linkers were then quenched by supplementing cell suspensions with 1 m ice-cold glycine to reach a final concentration of 125 mM and rocked for a further 5 min at room temperature. Cross-linked cells were then pelleted at 3200*g* for 5 min at 4°C, washed with 1 mL of ice-cold PBS with 5 mM EDTA and protease inhibitors, and then immediately snap-frozen with dry ice and stored at −80°C until ChIPs were performed. For treatment-based ChIP experiments (following vehicle controls or DOX or A-485 treatment), 50,000 (10%) single-cross-linked E14 mouse ESCs were additionally spiked in prior to each ChIP to act as exogenous normalization references in case global changes to histone modifications were observed.

Cross-linked cell pellets were washed twice with 1 mL of ice-cold NP buffer (10 mM Tris-HCl, 1 m sorbitol, 50 mM NaCl, 5 mM MgCl_2_, 1 mM CaCl_2_, 0.1% Igepal CA-630, 0.1% Tween-20 in H_2_O freshly supplemented with 0.385 µM β-mercaptoethanol, 1.82 mM spermidine, 1× protease inhibitor tablet) to isolate nuclei, and nuclei were spun at 2000*g* for 5 min at 4°C each time for pelleting. Nuclei were resuspended in 900 µL of ChIP buffer (20 mM Tris-HCl, 2 mM EDTA, 150 mM NaCl, 0.6% SDS, 1% Triton X in H_2_O freshly supplemented with 0.1 µM PMSF, 1× protease inhibitor tablet), transferred to a milliTUBE AFA Snap Fibre sonication vial (Covaris 520135), and left to incubate for 1 h on ice. Chromatin was then sheared to ∼200 bp fragments using the ME220 sonicator (Covaris) at 4°C, 75% power, and 15% duty factor for 1000 cycles for either 30 min (double-cross-linked) or 20 min (single-cross-linked). Debris were pelleted at 12,000*g* for 15 min at 4°C, and the supernatant containing sheared chromatin was transferred to a new tube. Sheared chromatin was regularly checked through gel electrophoresis to ensure predominantly mononucleosomal fragments. Protein G Dynabeads were used for double-cross-linked ChIPs, and protein A Dynabeads were used for single-cross-linked ChIPs. Fifty microliters of beads was washed twice in 1 mL of low-salt wash buffer (20 mM Tris-HCl, 2 mM EDTA, 150 mM NaCl, 0.1% SDS, 1% Triton X in H_2_O) and resuspended in 100 µL of low-salt buffer. To preclear chromatin, 20 µL of washed beads was added to 180 µL of sheared chromatin and then incubated on a rotator for 5 h at 4°C, after which the supernatant was transferred to a new tube. To conjugate antibodies to beads, 0.5–4 µg of antibodies was diluted to 320 µL with ChIP dilution buffer (100 mM NaCl, 0.02% sodium azide, 100 mM Tris-HCl, 5 mM EDTA, 5% Triton X in H_2_O supplemented with 1× protease inhibitor tablet) and left to conjugate with 80 µL of washed beads while rotating for 5 h at 4°Cusing 0.5–4 µg of mouse IgG (Invitrogen 31903) and 0.5–4 µg of rabbit IgG (Invitrogen 012-6102) as IgG controls or the following antibodies: 4 µg of DPPA2 (Sigma MAB4356), 4 µg of DPPA4 (Abcepta AP1438a, Abcepta AP21202c, and Abcam ab154642), 4 µg of KDM2A (Invitrogen PA5-40421), 1 µg of SUZ12 (Cell Signaling Technology 3737), 0.5 µg of H3K4me3 (Cell Signaling Technology 9751), 0.5 µg of H3K27me3 (Cell Signaling Technology 9733), 0.5 µg of H2AK119ub (Cell Signaling Technology 8240), 0.5 µg of H3K4me1 (Cell Signaling Technology 5326T), 1 µg of H3K27ac (Abcam ab4729), 1 µg of H3K9me3 (Abcam ab8898), 0.5 µg of H3K36me2 (Abcam ab9049), and 0.5 µg of H3K36me3 (Cell Signaling Technology 4909). Antibody–bead conjugates were washed with 1 mL of ChIP dilution buffer, subsequently resuspended with 180 µL of precleared chromatin, and left to incubate while rotating overnight at 4°C, and 9 µL of the remaining precleared chromatin was set aside as an input control. Beads were then washed three times with 200 µL of low-salt wash buffer, three times with 200 µL of high-salt wash buffer (20 mM Tris-HCl, 2 mM EDTA, 500 mM NaCl, 0.1% SDS, 1% Triton X in H_2_O), twice with 200 µL of LiCl wash buffer (250 mM LiCl, 1% sodium deoxycholate, 10 mM Tris-HCl, 1 mM EDTA, 1% Igepal CA-630), and once with 200 µL of 10 mM Tris-HCl (pH 7.5). Beads as well as input controls were then resuspended/diluted in 50 µL of ChIP elution buffer (1% SDS, 0.1 m NaHCO_3_ in H_2_O). Samples were subjected to digestion with 5 µL of 20 mg/mL RNase A (NEB) for 1 h at 37°C and then digestion with 5 µL of 800 U/mL proteinase K (NEB) for 1 h at 37°C, and cross-links were reversed by incubation on a thermomixer at 1200 rpm overnight at 65°C. DNA was then enriched from eluates using a 1.8× bead cleanup (SergiLabs) and eluted in 30 µL of H_2_O for downstream qPCR analyses and DNA library preparation.

### Sequential chromatin immunoprecipitation (reChIP)

For each reChIP, 1 × 10^6^ single-cross-linked NCI-H661 cells were used. Pellets of single-cross-linked NCI-H661 cells were washed twice with 1 mL of NP buffer (10 mM Tris-HCl, 1 m sorbitol, 50 mM NaCl, 5 mM MgCl_2_, 1 mM CaCl_2_, 0.1% Igepal CA-630, 0.1% Tween-20 in H_2_O freshly supplemented 1× protease inhibitor tablet) on ice. Nuclei were pooled and resuspended in 800 µL of ChIP buffer and then left to incubate for at least 5 min on ice. Chromatin was sheared (as above for single-cross-linked cells), and debris were pelleted at 12,000*g* for 15 min at 4°C. The supernatant containing chromatin was precleared by adding 20 µL of washed protein A Dynabeads (as above, resuspended in ChIP dilution buffer [67 mM Tris-HCl, 100 mM NaCl, 5 mM EDTA, 0.33% SDS, 1.67% Triton X]) and incubated with rotation for at least 3 h at 4°C. Ten microliters of the precleared chromatin was set aside as 5% input. For each reChIP, 200 µL of precleared chromatin (1 × 10^6^ cells) was made up to 500 µL in a dilution buffer to get a final concentration of 67 mM Tris-HCl, 100 mM NaCl, 5 mM EDTA, 0.33% SDS, 1.67% Triton X, 0.1 µM PMSF, and 1× protease inhibitors (reChIP buffer). Twenty microliters of protein A Dynabeads was preincubated (as above) with either 0.5 µg of H3K4me3 (Cell Signaling Technology 9751) or H2AK119ub (Cell Signaling Technology 8240) antibodies or rabbit IgG (Invitrogen 02-6102) and then resuspended in the 500 µL of diluted chromatin. The first immunoprecipitation was performed overnight at 4°C with rotation, and antibody–chromatin complexes were then washed three times in 500 µL of low-salt buffer (as above), three times in 500 µL of high-salt buffer (as above), twice in 200 µL of LiCl buffer (as above), and twice in 500 µL of 10 mM Tris-HCl (pH 7.5) on ice. Complexes were eluted in 100 µL of ChIP elution buffer (as above) supplemented with 0.1 µM PMSF and 1× protease inhibitors on a thermomixer at 300 rpm for 30 min at 37°C, and 10 µL was put aside as a 10% single-ChIP control. To dilute the SDS, the eluate volume was increased to 300 µL with reChIP buffer and purified using Amicon Ultra 0.5 mL 3 kDa filter columns (Millipore UFC5003) according to the manufacturer's instructions, with chromatin being washed twice more with 400 µL of ChIP dilution buffer. Purified chromatin was made up to 500 µL in reChIP buffer for the second immunoprecipitation, which was performed using the alternate antibody or IgG control bead conjugates overnight at 4°C with rotation. Complexes were washed (as above) and resuspended in 100 µL of ChIP elution buffer. Samples were treated with 2 µL of RNase A and 2 µL of proteinase K and then reverse cross-linked (as above). DNA was purified using the Monarch PCR and DNA cleanup kit (NEB) and eluted in 25 µL of H_2_O for qPCR and library preparation for sequencing.

### Cleavage under targets and release using nuclease (CUT&RUN)

NCI-H661 or NCI-H1299 cells were harvested and counted after staining cells with 0.4% Trypan blue, where 500,000 live cells were used per CUT&RUN. Cells were washed with ice-cold cell wash buffer (150 mM NaCl, 20 mM HEPES in H_2_O) and kept on ice. Eleven microliters of Concanavalin A beads (EpiCypher) was washed twice with 100 µL of cold bead activation buffer (20 mM HEPES, 50 µL of KCl, 1 mM CaCl_2_, 1 mM MnCl_2_ in H_2_O) and then resuspended in 11 µL of the same buffer on ice. Cells were washed twice with NE buffer (20 mM HEPES, 10 mM KCl, 0.1% Triton X, 20% glycerol in H_2_O) to release nuclei, with gentle pipetting and 300*g* spins for 5 min at 4°C in between each wash. Nuclei were resuspended in 100 µL of cold cell wash buffer and then incubated for 10 min at room temperature with 10 µL of washed beads to allow for nucleus bead adsorption. Beads were then resuspended in 50 µL of cold antibody buffer (20 mM HEPES, 150 mM NaCl, 0.5 mM spermidine, 0.05% digitonin, 2 mM EDTA in H_2_O) followed by addition of 1 µg of IgG controls (mouse IgG [Invitrogen 31903] and rabbit IgG [Invitrogen 012-6102]) or antibodies (DPPA2 [SigmaMAB4356], DPPA4 [Abcepta AP1438a, Abcepta AP21202c, and Abcam ab154642], V5 tag [Invitrogen 46-0705], FLAG tag [Merck F1084 for monoclonal and Invitrogen 740001 for superclonal], RING1B [Cell Signaling Technology 5694], PCGF1 [Santa Cruz Biotechnology sc515371], MTF2 [ProteinTech 16208-1-AP], EP300 [Aviva Systems Biology OAAF01891]) or 0.5 µg for the H3K4me3 (Cell Signaling Technology 9751) positive control and incubated overnight at 4°C on a nutator. Bead complexes were then washed twice with 200 µL of cold antibody buffer (same as above but without EDTA) and resuspended in 50 µL of the same buffer. Protein-A/G-MNase (1.5 µL; Cell Signaling Technology) was added to each bead suspension, mixed in a pipette, and incubated for 10 min at room temperature. Beads were washed twice with 250 µL of cold antibody buffer and resuspended in 50 µL of the same buffer. One microliter of 100 mM CaCl_2_ was added to each bead suspension to activate the bound MNase, followed by incubation on a thermal cycler for 1 h at 4°C to facilitate chromatin digestion. Thirty-eight microliters of stop buffer (340 mM NaCl, 20 mM EDTA, 4 mM EGTA, 50 µg/mL RNase A, 50 µg/mL glycogen in H_2_O) containing 250 pg of *Escherichia coli* normalization spike-in DNA (Cell Signaling Technology) was added to each suspension to inhibit MNase, degrade RNA, and release digested chromatin into the supernatant by incubation in a thermal cycler for 10 min at 37°C. The supernatant containing digested chromatin was transferred to a new tube and treated with 1 µL of 20 mg/mL proteinase K for 1 h at 37°C. DNA was purified using a 1.8× bead cleanup (SergiLabs) and eluted in 30 µL of H_2_O for DNA library preparation.

### In vivo animal studies

NCI-H1299 cells containing doxycycline-inducible overexpression pCLIIPi-FLAG-DPPA2-PGK-mVenus and pCLIIPi-V5-DPPA4-PGK-tagBFP vectors stably integrated into the genome were induced with H_2_O or 2 µg/mL doxycycline for 72 h in culture. Cells were harvested and counted following 0.4% Trypan blue staining, wherein 5 × 10^6^ cells were prepared in 50 µL of RPMI-1640 with 50 µL of Matrigel (Corning) per mouse (10 mice per condition) without serum. One-hundred microliters of cells was subcutaneously injected into the right flank of 5–7 week old male BALB/c nude mice (ARC). Mice were fed normal chow or chow containing 600 mg/kg doxycycline 2 days after to maintain induction (10 mice per condition).

NCI-H661 cells containing doxycycline-inducible shRNA pCLIIPi (shREN-713, shDPPA2-760, shDPPA4-1993, or shDPPA2+4) vectors (Supplemental Table S14) stably integrated into the genome were harvested and counted following 0.4% Trypan blue staining, wherein 1 × 10^6^ cells were prepared in 50 µL of RPMI-1640 with 50 µL of Matrigel (Corning) per mouse (20 mice per shRNA line) without serum. One-hundred microliters of cells was subcutaneously injected into the right flank of 5–6 week old male NOD SCIDγ (NSG) mice (ABR). Tumors were allowed to form for the next 97 days while mice were fed normal chow. At day 97, mice from each line were randomized into two groups of 10 and fed normal chow or chow containing 600 mg/kg doxycycline to induce shRNA expression. Tumor volume was monitored until end points were reached (day 147 [50 days following randomization] or tumor volume of 1200 mm^3^).

Tumor volume was determined by electronic calipers, and body weight was determined by electronic scales at least weekly until tumors reached 1200 mm^3^ volume or lost >20% of their body weight, at which point mice were humanely euthanized, and organs as well as the tumor were harvested and weighed according to ethics application #2023-06, approved by the Peter MacCallum Cancer Centre (PMCC) Animal Ethics Committee. This work was conducted with the Translational Research Centre (TRC) at PMCC.

### Resource availability

#### Resources and reagents

Sequences for shRNA, siRNA, and primers are shown in Supplemental Tables S14–S16. Further information and requests for resources and reagents should be directed to and will be fulfilled by the lead contact, M.A.E.-M. (melanie.eckersley-maslin@petermac.org).

#### Materials availability

All plasmids and cell lines generated in this study are available upon request.

#### Data and code availability

Sequencing data are available through GEO under accession number GSE310173. The mass spectrometry proteomics data have been deposited to the ProteomeXchange Consortium via the PRIDE partner repository with the data set identifiers PXD070581 and PXD070627. Imaging, EMSA, Western blot, and other source data are available on Mendeley Data. No original code was developed as part of this project.

## Supplemental Material

Supplement 1

Supplement 2

Supplement 3

Supplement 4

Supplement 5

Supplement 6

Supplement 7

Supplement 8

Supplement 9

Supplement 10

Supplement 11

Supplement 12

Supplement 13

Supplement 14
