# Supplementary material for: Embryonic stem cell factors DPPA2/4 amplify active H3K4me3–H2AK119ub chromatin domains in non-small cell lung cancer
Source: Genes Dev. 2026 May 1;40(9-10):756–76. doi: 10.1101/gad.353102.125 (PMC13138344; doi:10.1101/gad.353102.125)
Supplement: Supplement 4 [file Supplemental_Information.pdf]

Supplemental Information: Seneviratne et al. Embryonic stem cell factors DPPA2/4 amplify active H3K4me3-H2AK119ub chromatin domains in non-small cell lung cancer

Supplemental Figure 1, related to Figure 1

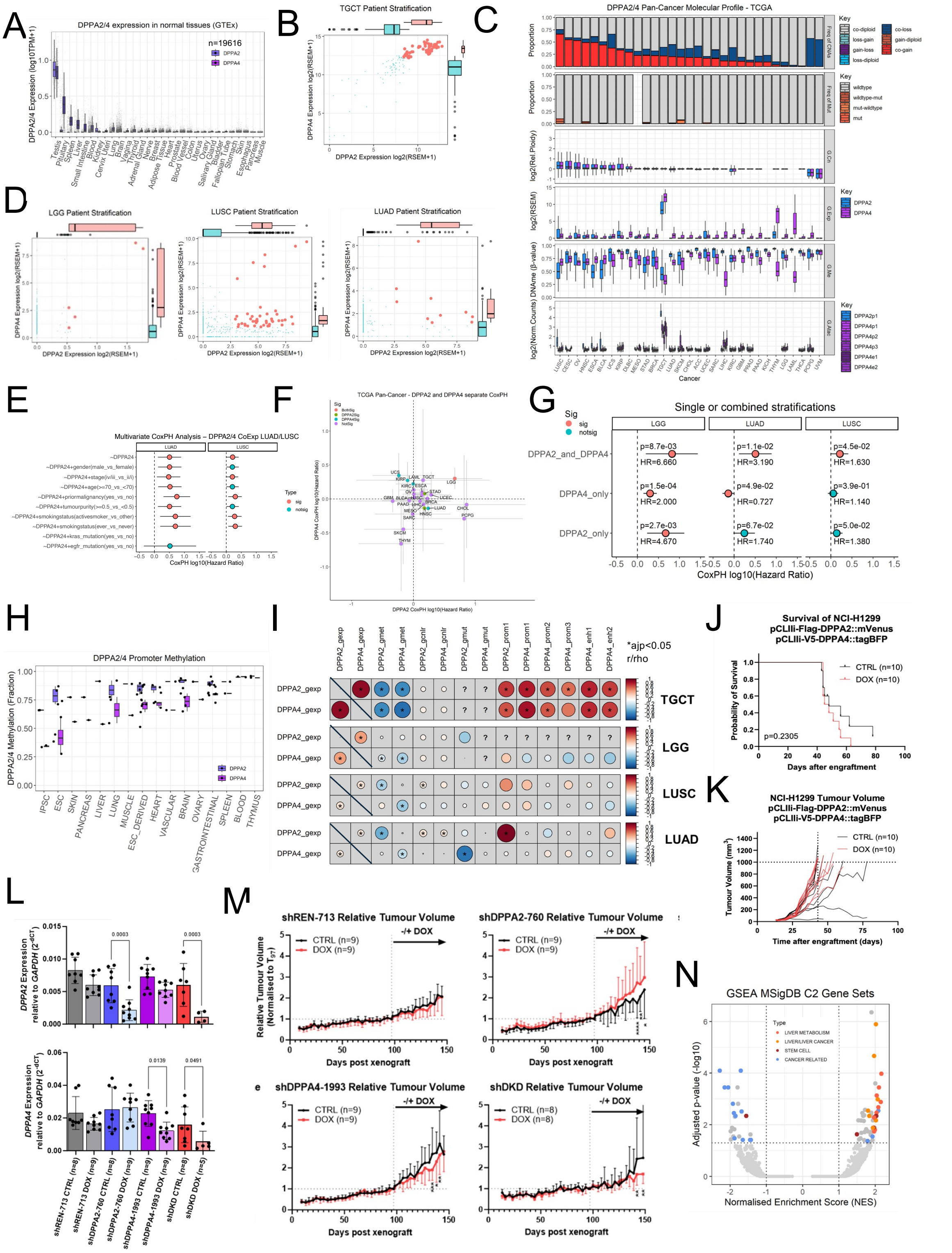

Supplemental Figure 1, related to Figure 1
a) GTEx normal adult tissue DPPA2/4 expression in boxplots (n=19616, units are in log10TPM+1).
b) DPPA2/4 expression in the TCGA TGCT cohort stratified by expression group (co-expressing and not co-expressing) (units are in log2RSEM+1 counts). Boxplots shown adjacent to the axes represent the expression of DPPA2 and DPPA4 in either group (median, whiskers represent the lower and upper 95% confidence intervals).
c) Pan-cancer molecular profile of DPPA2/4 across 31 cancer types from TCGA. The first panel summarises the proportion of patients with a copy number aberrations on the DPPA2/4 gene body (WES; copy number ≥ 3 gain, copy number ≤ 1 loss, copy number = 2 diploid). The second panel summarises the proportion of patients with somatic mutations on the DPPA2/4 gene body (WES). The third panel indicates the relative ploidy (copy number) of DPPA2/4 as boxplots for either DPPA2/4 (WES; log2RelativePloidy). The fourth panel indicates the gene expression of DPPA2/4 as boxplots for either DPPA2/4 (RNA-seq; log2RSEM). The fifth panel indicates the methylation level of DPPA2/4 gene promoters (TSS+/-1.5kb) as boxplots for either DPPA2/4 (methylation array; β-value). The sixth panel indicates the chromatin accessibility of DPPA2/4 gene promoters (DPPA2p1, DPPA4p1, DPPA4p2, DPPA4p3) or putative enhancers (DPPA4e1, DPPA4e2) as boxplots for either DPPA2/4 (ATAC-seq; log2Normalised Counts).
d) DPPA2/4 expression in the TCGA LGG, LUSC and LUAD cohorts stratified by expression group (co-expressing and not co-expressing) (units are in log2RSEM+1 counts). Boxplots shown adjacent to the axes represent the expression of DPPA2 and DPPA4 in either group (median, whiskers represent the lower and upper 95% confidence intervals).
e) CoxPH models are shown for TCGA LUSC and LUAD cohorts when patients were subdivided by DPPA2+4 co-expression and then subject to multivariate regression analyses against several clinical covariates (gender (male vs female), disease stage (stages 3&4 vs 1&2), age (greater than 70 years vs less than 70 years of age), whether the patient had a malignancy prior to diagnosis (yes vs no), tumour purity (>0.5 vs < 0.5 tumour fraction), smoking statuses (either active vs non-active smoker or ever vs never smoker) as well as EGFR and KRAS mutational status (these were only present in LUAD and could not be tested in LUSC)) to determine prognostic independence. The x-axis indicates the hazard ratio (HR) of CoxPH models (log10 transformed) with p-values of the model reported beside and each point highlighted by significance (p-value < 0.05).
f) Scatter plot of TCGA cancer cohort Cox proportional hazard modelling (CoxPH) of patient outcomes, where patients in each cohort was subdivided by the expression of either DPPA2 or DPPA4 as determined by RNA-seq (expression cut-off defined as the median of all DPPA2 or DPPA4 expressors (RSEM>0)). The x-axis and y-axis indicates the hazard ratio (HR) of CoxPH models (log10 transformed) for DPPA2 and DPPA4 respectively. Dots are coloured to indicate significant models (absolute HR > 1 and p-value < 0.05) for either/both DPPA2 and DPPA4 models, whiskers represent the lower and upper 95% confidence intervals of HRs for each model.
g) Select CoxPH models are shown for TCGA LGG, LUSC and LUAD cohorts when pateints were subdivided by DPPA2, DPPA4 or DPPA2+4 expression. The x-axis indicates the hazard ratio (HR) of CoxPH models (log10 transformed) with p-values of the model reported beside and each point highlighted by significance (p-value < 0.05).
h) Boxplots of DPPA2/4 promoter (TSS+/-1500bp) DNA methylation in a range of human cell line models spanning human development from the epigenetic roadmap consortium (ERM). DNA methylation is presented as fraction methylated, with groups of cell lines ordered by decreasing average DPPA2/4 promoter DNA methylation.
i) Dot plot visualising the correlation coefficient (biserial correlation for mutations and pearson correlation for all others) of DPPA2/4 gene expression (RNA-seq) correlated with their gene expression (RNA-seq), promoter DNA methylation (methylation arrays), gene copy number

(WES), gene somatic mutation (WES) and chromatin accessibility at promoters or distal enhancers (ATAC-seq) in TCGA TGCT, LGG, LUSC and LUAD cancer cohorts. Significant correlations (BH-adjusted p-value < 0.05) are marked with an asterisk*. Those correlations for which there was no accessibility data or lack of mutations are marked with a question mark (?).
j) Kaplan-Meier survival curves of Balb/c nude mice xenografted with NCI-H1299 DPPA2/4 double overexpression lines (iFlag-DPPA2 and iV5-DPPA4). Lines were pre-treated with a vehicle control (H_20) or doxycycline 2µg/mL doxycycline (DOX) for 72h prior to engraftment and then xenografted mice were respectively kept on control or doxycycline feeds to maintain overexpression. Endpoint is tumour volume reaching 1000mm^3. P-value is computed from a log-rank test.
k) Tumour volumes (mm^3) of xenografted mice in **j**).
l) NCI-H661 cells harbouring stable inducible shRNA constructs (shREN-713, shDPPA2-760, shDPPA4-1993 or shDPPA2+4) were xenografted into the flanks of NSG mice at $1x10^6$ cells per mouse and allowed to engraft for 97 days before randomisation into two feed groups (control and doxycycline feed) for another 50 days. The data represent RT-qPCR of DPPA2 (upper panel) and DPPA4 (lower panel) gene expression (relative to GAPDH) in the xenografted tumours at the study endpoint (day 147 post implantation). Significant comparisons (p<0.05) are annotated (one-way ANOVA), error bars represent the standard error of the mean for 5-9 mice per group.
m) Tumour growth curves (relative to the volume at the day of randomisation (day 97)) where error bars represent the standard deviation of the mean for 8-9 mice per group, where significant comparisons (p<0.05) are annotated (two-way ANOVA; *p<0.05, **p<0.01, ***p<0.001, ****p<0.0001). The randomisation/treatment period is indicated in the plot.
n) Volcano plot illustrating MSigDB C2 gene sets after a GSEA using all genes ranked by log2FC comparing DPPA2/4 co-expressing vs other NSCLC (LUAD+LUSC) tumours. Depleted gene sets are determined as those with NES < -1 and adjusted p-value < 0.05 and enriched gene sets are determined as NES > 1 and adjusted p-value < 0.05. Enriched gene sets falling into broad categories are annotated.

Supplemental Figure 2, relating to Figure 2

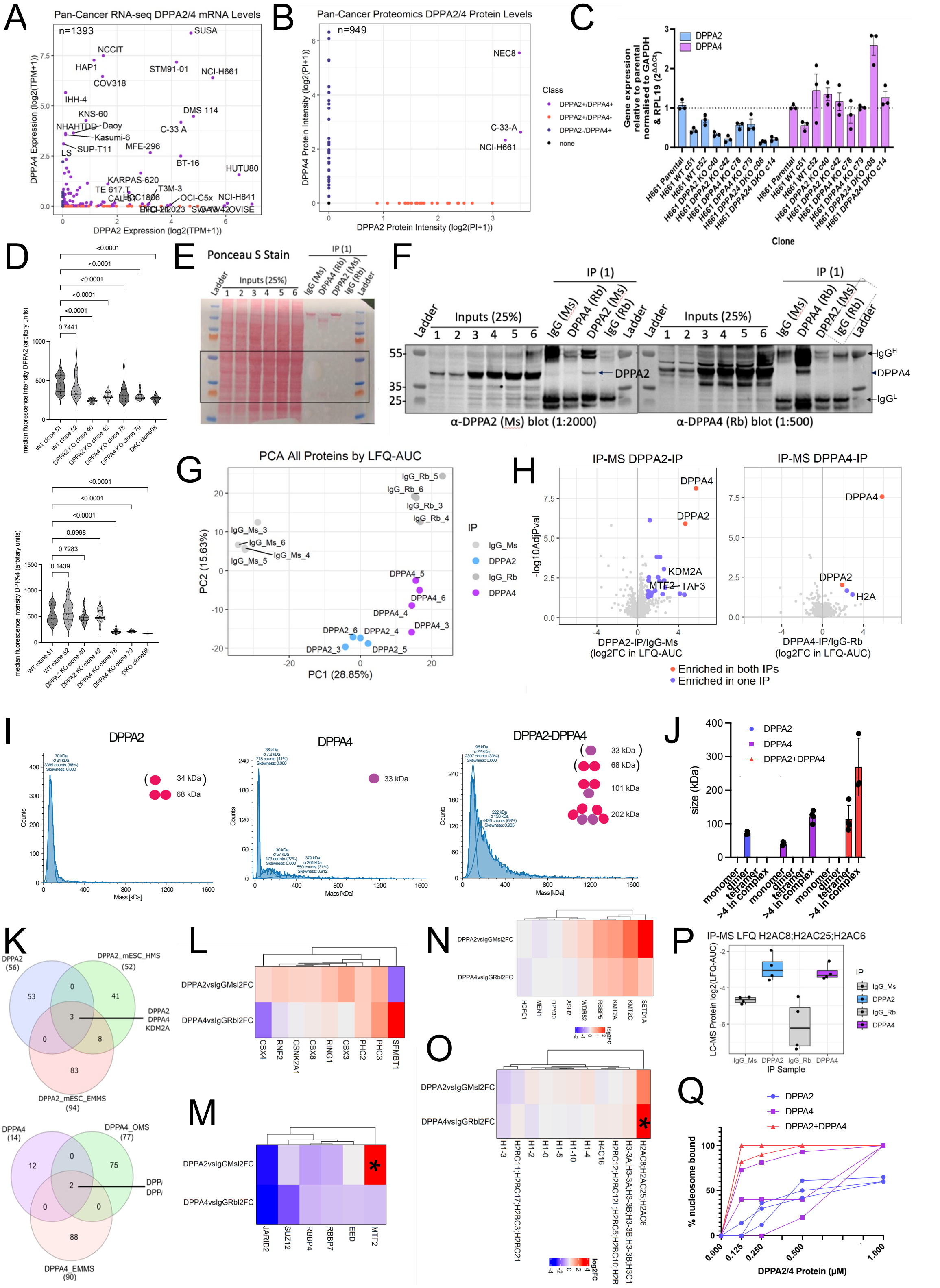

Supplemental Figure 2, relating to Figure 2.

a) Scatter plot of DPPA2 (x-axis) and DPPA4 (y-axis) gene expression across cancer cell lines (n=1393) in the CCLE (DepMap; RNA-seq) (units are log2TPM+1). Cell lines are coloured by whether DPPA2 and/or DPPA4 are expressed, where co-expressing cell lines are labelled.

b) Scatter plot of DPPA2 (x-axis) and DPPA4 (y-axis) protein levels across cancer cell lines (n=949) from Goncalves et al 2022 (Mass Spectrometry) (units are log2 Protein Intensity +1). Cell lines are coloured by whether DPPA2 and/or DPPA4 are detected, where co-detected cell lines are labelled.
c) Column graphs of relative DPPA2 and DPPA4 gene expression (relative to parental line and normalised to GAPDH & RPL19 housekeeping controls) in parental and isogenic NCI-H661 clones following CRISPR-Cas9 knockout clones and RT-qPCR (n=3 independent passages).
d) Violin plots of quantified median fluorescence intensity from DPPA2 and DPPA4 immunoflourescence for each isogenic cell line (2 clones per genotype, X images, N fields per image, X magnification).
e) Brightfield image of western blot membrane following reversible staining with the Ponceau S protein stain. The molecular ladders are present in the first and last lane. Six lanes indicate the 25% of the whole cell lysate inputs (Replicate 1-6) used for the immunoprecipitations. The next four lanes indicate a representative immunoprecipitation (Replicate 1 only eluted and blotted for, Replicate 2-6 were submitted for mass spectrometry following on-bead tryptic digests) with IgG (mouse), DPPA4 (rabbit), DPPA2 (mouse) and IgG (rabbit) antibodies respectively.
f) Western blots of above membrane following incubation with anti-DPPA2 or anti-DPPA4 antibodies. The expected sizes of DPPA2 and DPPA4 are annotated, as well as light (25 kDa) and heavy (55 kDa) chain IgG antibody fragments following elution.
g) Principal component analysis (PCA) of mass spectrometry data using LFQ-AUC values for all proteins (n=4 biological replicates/independent lysates per condition). Samples are coloured by IP type, with PC1 and PC2 representing the most variable components (28.85% and 15.63% respectively).
h) Scatter plots of detected nuclear proteins in the IP-MS data (subset of all proteins with an annotated nuclear localisation within the human protein atlas). Significantly enriched proteins (relative to IgG controls) are annotated (log2FC ≥ 1, BH-adjusted p-value < 0.05).
i) Representative histograms of mass photometry data of recombinant DPPA2-Myc/Flag and/or His-DPPA4 proteins, where the x-axis represents mass (kDa) and y-axis represents photometry counts. Called photometry peaks are annotated with median size (kDa), standard error (σ) and skewness. Schematic of DPPA2 (red) and DPPA4 (purple) oligomeric complexes are shown. The experiments were repeated three times independently.
j) Bar plots summarising mass photometry peak sizes of recombinant DPPA2-Myc/Flag and/or His-DPPA4 proteins (n=three to four experimental replicates) at different complex stoichiometries
k) Venn diagrams summarising the overlap of our IP-MS findings with other studies; For DPPA2-IP: 1) Eckersley-Maslin et al. 2020 NSMB, overexpression of DPPA2/4-GFP, IP using GFP-trap followed by RIME-MS in mESC's, 2) Hernandez et al. 2018 Cell Stem Cell, overexpression of 3xFlag-DPPA2, following Flag-IP and MS in mESCs. For DPPA4-IP: 1) Eckersley-Maslin et al. 2020 NSMB as above, 2) Oliviera et al. 2014, endogenous DPPA4-IP followed by MS in NT2 human cells.
l,m,n,o) Summary of enrichment (log2FC IP vs IgG) in IP-MS for detected proteins belonging to PRC1, PRC2, COMPASS or histones. Significant enrichments are denoted by an asterisk (* BH adjusted p-value < 0.05 and log2FC ≥ 1).
p) Enrichment (log2FC IP vs IgG) of H2A peptides in IP-MS
q) Line curves summarising the binding of DPPA2-Myc/Flag and/or His-DPPA4 proteins to nucleosomes in EMSA experiments (n=3 experimental replicates), represented as the percentage of total nucleosomes bound.

Supplemental Figure 3, related to Figure 3

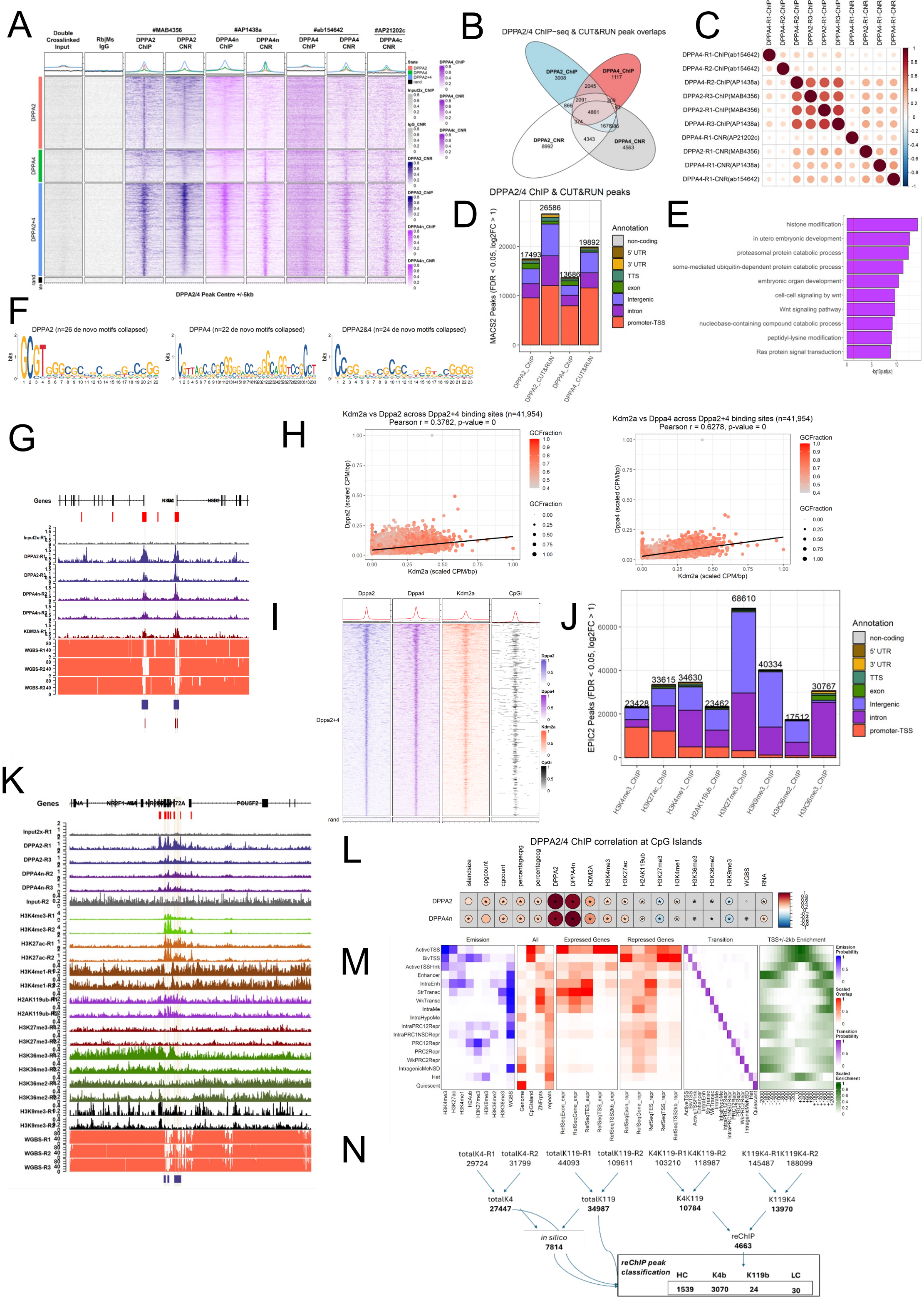

Supplemental Figure 3, relating to Figure 3

a) Heatmaps of genomic regions centred on DPPA2/4 peaks as determined by ChIP or CUT&Run (resized to +/-5kb from the centre of each peak, split into 100 equal windows) as well as a set of background regions not bound by DPPA2+4 for comparison (DPPA2 (n=12866),

DPPA4 (n=5773), DPPA2+4 (n=16620) and background (n=1000). Columns represent the double crosslinked input ChIP control (n=1), averaged mouse/rabbit IgG CUT&Run control (n=1 each), average DPPA2 (n=2) ChIP (MAB4356), DPPA2 (n=1) CUT&Run (MAB4356), average DPPA4 (n=2) ChIP (AP1438a), DPPA4 (n=1) CUT&Run (AP1438a), average DPPA4 (n=2) ChIP (ab154642) , DPPA4 (n=1) CUT&Run (ab154642), DPPA4 (n=1) CUT&Run (AP21202c). ChIP and CUT&Run CPM/bp values were scaled per bin across all regions for visualisation.

b) Venn diagram of ChIP (overlapping peaks in 2 replicates, determined as those peaks overlapping by atleast 25%) and CUT&Run (1 replicate) peak overlaps for DPPA2 (MAB4356) and DPPA4 (AP1438a) using MACS2 (FDR < 0.05 and log2FC > 1 compared to matched Input or IgG controls).

c) Genome-wide pairwise Pearson correlations of ChIP-seq and CUT&Run (DPPA2 (n=2) ChIP (MAB4356), DPPA2 (n=1) CUT&Run (MAB4356), DPPA4 (n=2) ChIP (AP1438a), DPPA4 (n=1) CUT&Run (AP1438a), DPPA4 (n=2) ChIP (ab154642), DPPA4 (n=1) CUT&Run (ab154642), DPPA4 (n=1) CUT&Run (AP21202c) fold enrichment over the matched input or IgG control) partitioned into 200bp bins genome-wide. Dots are coloured and sized by Pearson correlation coefficients (r) and are ordered by hierarchical clustering with red squares denoting major clusters.

d) HOMER genomic annotations of DPPA2 and DPPA4 ChIP/CUT&Run peaks (determined by MACS2, FDR < 0.05, log2FC > 1). Promoter-TSS is defined as TSS+/-1500bp. The numbers atop each column indicates the total number of DPPA2/4 peaks (for ChIPs these are the number of overlapping peaks (>25%) between two replicates, for CUT&Run these are from one replicate each).

e) Gene ontology (GO-Biological Processes) enrichment analysis of DPPA2+4 bound gene promoters as determined by clusterProfileR. The red line indicates the significance threshold at adjusted p-value 0.05.

f) Collapsed HOMER *de novo* motifs from DPPA2-only (n=12866), DPPA4-only (n=5773) and DPPA2+4 ChIP/CUT&Run peaks (n=16620) relative to a set of background genomic regions (n=16620) size and gc-content matched to the DPPA2/4-peak set. All regions were resized to 1000bp prior to analyses.

g) Genomic track plot of the NSD2 (hg38 chr4: 1815954-1905116) locus, where a gene annotation track is provided at the top, followed by CpG island annotations, the double crosslinked input ChIP control (n=1), DPPA2 (n=2), DPPA4 (n=2) and KDM2A (n=1) ChIP and DNA methylation (WGBS) (n=3), followed by consensus DPPA2+4 peak and KDM2A peak regions annotated at the bottom. Scales are in CPM/bp for ChIP and %DNA methylation for WGBS.

h) Dot plots of Kdm2a compared to Dppa2 (left) or Dppa4 (right) ChIP signals (CPM/bp) across Dppa2/4 bound sites (n=41954) in E14 mESCs. Each site (dot) is coloured and sized by their GC fraction. Linear regression curves are also fit for each comparison with Pearson correlation statistics above each plot. Dppa2/4 ChIP data are reanalysed from Hernandez et al, 2018 Cell Stem Cell and Kdm2a ChIP data were obtained from Blackledge et al, 2010 Molecular Cell.

i) Heatmaps of genomic regions centred on Dppa2/4 peaks in E14 mESCs as determined by ChIP (resized to +/-5kb from the centre of each peak, split into 100 equal windows) as well as a set of background regions not bound by Dppa2+4 for comparison (DPPA2+4 (n=41954) and background (n=1000). Columns represent Dppa2, Dppa4 and Kdm2a ChIP, as well as a CpGi density track (mm10). CPM/bp values were scaled per bin across all regions for visualisation.

j) HOMER genomic annotations of H3K4me3, H3K27ac, H3K4me1, H2AK119ub, H3K27me3, H3K9me3, H3K36me2 and H3K36me3 ChIP peaks (determined by SICER2, FDR < 0.05, log2FC > 1). Promoter-TSS is defined as TSS+/-1500bp. The numbers atop each column indicates the total number of peaks (these are the number of overlapping peaks (>25%) between two replicates).

k) Genomic track plot of the chr 5:93429332-93810092 (hg38) locus, where a gene annotation track is provided at the top, followed by CpG island annotations, the double crosslinked input

ChIP control (n=1), DPPA2 (n=2), DPPA4 (n=2) ChIP, single crosslinked input ChIP control (n=1), H3K4me3 (n=2), H3K27ac (n=2), H3K4me1 (n=2), H2AK119ub (n=2), H3K27me3 (n=2), H3K36me3 (n=2), H3K36me2 (n=2), H3K9me3 (n=2) ChIP and DNA methylation (WGBS) (n=3), followed by consensus DPPA2+4 peak regions annotated at the bottom. Scales are in CPM/bp for ChIPs and percentage methylated DNA for WGBS (0-100%).

l) Dot plot visualising the correlation coefficient (Pearson correlation) of DPPA2/4 gene ChIP-seq signal across annotated CpG islands (n=32038) correlated with CpGi characteristics (island size in bp, CpG count, CG dinucleotide count, CpG percentage, CG dinucleotide percentage) and other ChIP data (KDM2A, histone modifications), WGBS and the gene expression of the nearest gene (RNA-seq). Significant correlations (BH-adjusted p-value < 0.05) are marked with an asterisk*.

m) 17-state chromatin model as determined by ChromHMM modelling. The first panel presents emission probabilities that summarise the association of each mark with each chromatin state. The second to fourth panel indicates the scaled overlap of each chromatin state with annotated features in the genome, note that RefSeq annotations are split according to expressed and repressed genes in NCI-H661, as determined by RNA-seq. The fifth panel indicates transition probabilities that indicate the relatedness between states. The sixth panel represents the scaled enrichment of each signal flanking the TSS+/-2kb.

n) H3K4me3-H2AK119ub reChIP peak calling and classification workflow. Here we overlapped peaks from 2 replicates (>25% overlap), and then for reChIP's overlapped consensus H3K4me3-H2AK119ub and H2AK119ub-H3K4me3 peaks to arrive at a final set of n=4663 reChIP peaks. These peaks were further subclassified using single H3K4me3 or H2AK119ub peak sets to classify peaks as either high confidence (HC, enriched in all conditions), H3K4me3-biased (K4b, enriched in H3K4me3, but not H2AK119ub), H2AK119ub-biased (K119b, enriched in H2AK119ub, but not H3K4me3) and low confidence (LC, not enriched in single ChIPs).

Supplemental Figure 4, relating to Figure 4

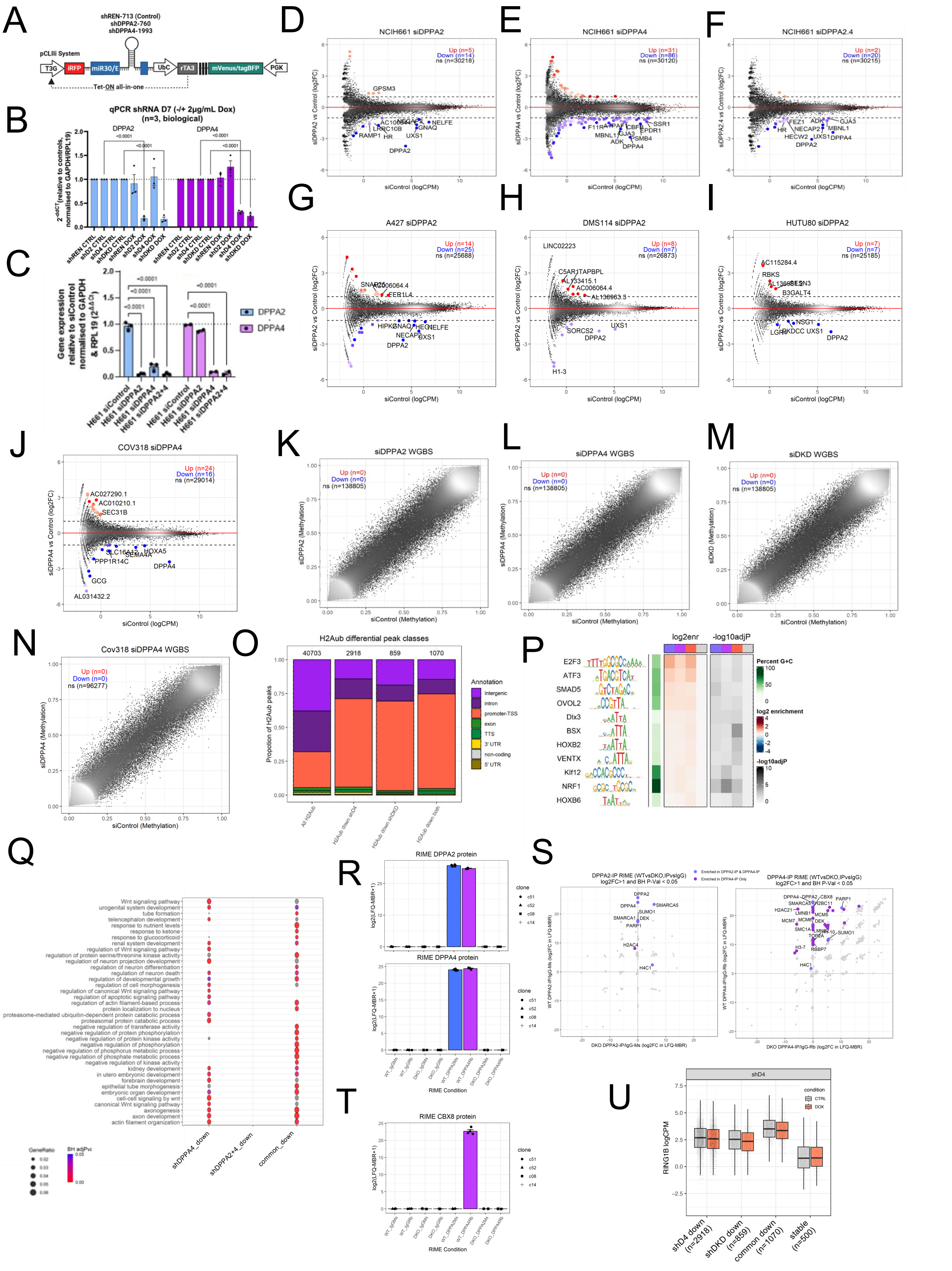

Supplemental Figure 4, related to Figure 4

a) Schematic of doxycycline inducible shRNA pCLIIi system.

b)RT-qPCR analyses of DPPA2 and DPPA4 (relative to RPL19 and GAPDH, and then to treatment controls) in stable NCI-H661 shRNA cell lines following induction with a vehicle control (H_2O) or

2µg/mL doxycycline (DOX) for 7 days. The error bars represent the standard error of the mean of n=3 biological replicates.
c) RT-qPCR analyses of NCI-H661 cells following siRNA mediated DPPA2 or/and DPPA4 knockdown for 4 days (relative to RPL19 and GAPDH, and then to treatment controls). The error bars represent the standard error of the mean of n=3 biological replicates.
d-f) Scatter plots of differential RNA-seq (n=3 for all conditions) in NCI-H661 following 4 days of siRNA mediated DPPA2 or/and DPPA4 knockdown. Differentially expressed genes are those with an FDR < 0.05 and absolute log2FC of ≥ 1 as determined by edgeR-TMM analyses between DPPA2 and/or DPPA4 siRNA vs control siRNA. The y-axes indicate the log2FC for siDPPA2/4 vs siControl, and the x-axes are the counts per million (logCPM) in the siControl for all genes.
g-j) Scatter plots as above for RNA-seq of A427, DMS114, HuTu80 and Cov318 DPPA2/4 knockdowns (n=3 for all conditions).
k-m) Scatter plots of differential whole genome bisulfite-seq (WGBS, n=3 for all conditions) in NCI-H661 following 4 days of siRNA mediated DPPA2 or/and DPPA4 knockdown. Differentially methylated CpGs are those with an FDR < 0.05 as determined by edgeR analyses between DPPA2 and/or DPPA4 siRNA vs control siRNA. The y and x-axes indicate the fraction of methylated reads for a given CpG in siDPPA2/4 vs siControl respectively. Note that only variable CpG's with coverage (i.e. those CpG's that weren't solely hypo(0)/hypermethylated(1) across all samples and those CpG's with atleast 1 read across all samples) were tested.
n) Scatter plots of WGBS as above for DPPA4 knockdown in Cov318 cells (n=3 for each condition).**o)** Proportional bar plot of HOMER genomic annotations in all H2AK119ub regions or in subsets of these regions that were depleted in shDPPA4/shDKD. The total number of peaks per category are annotated atop each bar.
p) Heatmap of known motifs enriched in shDPPA4/shDKD H2AK119ub depleted regions as determined by monaLisa analyses. Depleted regions were first resized to 4000bp, corresponding to the approximate median length of all depleted domains. Size and gc-matched regions were then sampled from the genome as background regions for the analysis (bck, n=859). The heatmap presents all motifs that were enriched over background (log2FC > 0.25 and BH-adjusted p-value < 0.05) in any depleted category. Motifs as well as their gc content are provided to the left of the plot.
q) Gene ontology (GO-Biological Processes) enrichment analysis of gene promoters overlapping H2AK119ub depleted regions (TSS+/-1500bp) as determined by clusterProfileR. Coloured dots represent terms passing the significance threshold at adjusted p-value 0.05 and their size reflects the proportion of genes represented by each depleted category and the total number of genes in the geneset.
r) Barplots of RIME DPPA2/4 proteins levels across NCI-H661 clones (WT: c51&c52, DKO: c08&c14) and conditions (IgG, DPPA2-IP, DPPA4-IP). Units are log2 transformed label free quantitation normalised using match between runs (LFQ-MBR).
s) Scatter plots of RIME enriched proteins in DPPA2-IP (left) and DPPA4-IP (right), axes represent enrichment (log2 fold change of LFQ-MBR values) between DPPA2/4-IP and respective IgG controls in WT clones (y-axis) and DKO clones (x-axis). Coloured dots are those proteins enriched in WT (IP vs IgG) comparisons alone (log2FC >1 & adjusted p-value <0.05) and not in DKO comparisons.
t) Barplot of RIME CBX8 protein levels across NCI-H661 clones (WT: c51&c52, DKO: c08&c14) and conditions (IgG, DPPA2-IP, DPPA4-IP). Units are log2 transformed label free quantitation normalised using match between runs (LFQ-MBR).
u) Boxplots of RING1B levels (log2 transformed CPM/bp) across H2Aub differential and stable regions in the H661 shDPPA4 inducible model (7 days of either control or doxycycline treatment).

Supplemental Figure 5, relating to Figure 5

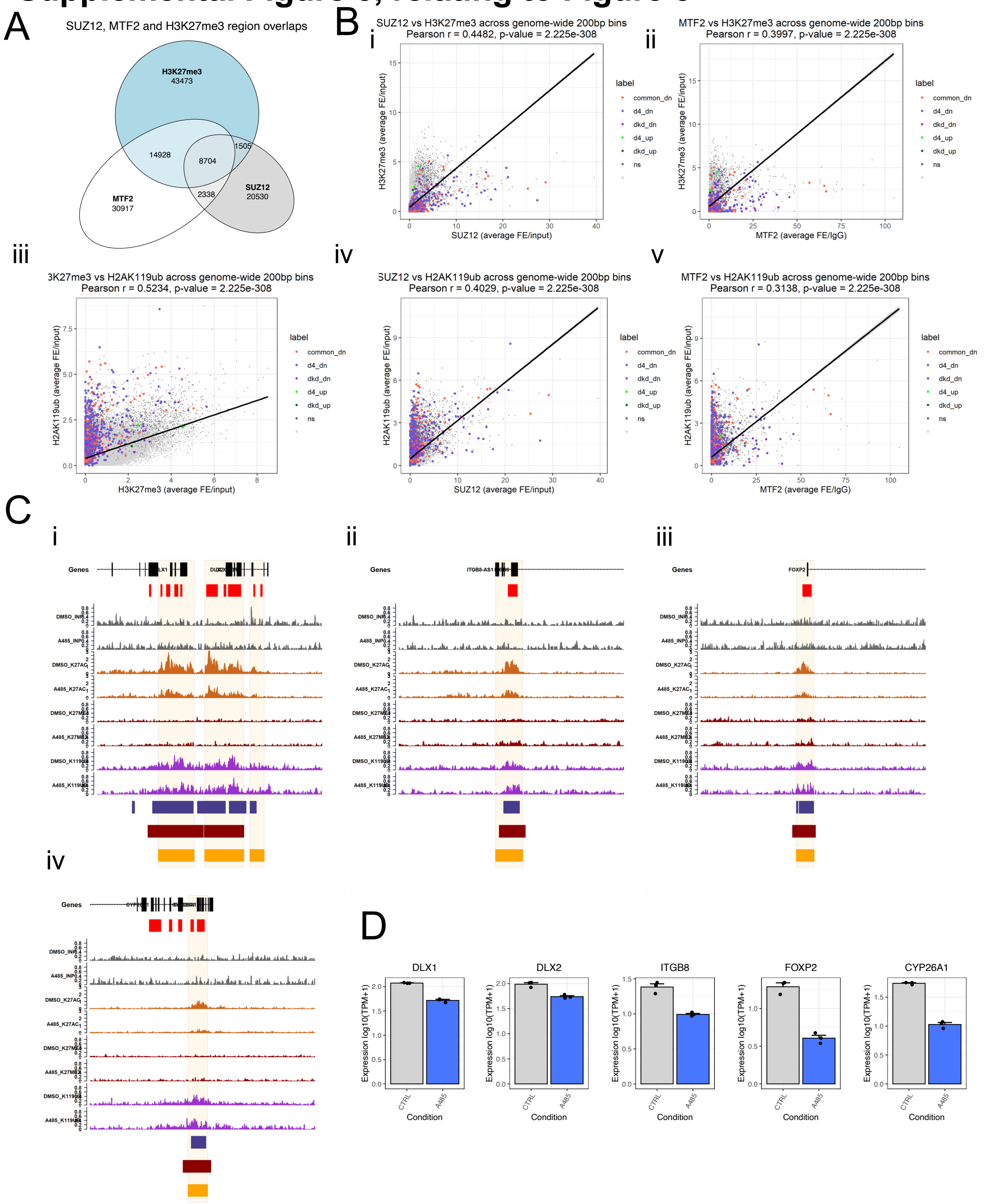

Supplemental Figure 5, related to Figure 5

a) Venn diagram of MTF2 (n=2) CUT&RUN, H3K27me3 (n=2) and SUZ12 (n=1) ChIP peak overlaps (overlapping peaks in 2 replicates for MTF2 and H3K27me3, determined as those peaks overlapping by at least 25%).

b) Genome-wide pearson correlations (200bp bins) between i) SUZ12 and H3K27me3; ii) MTF2 and H3K27me3; iii) H3K27me3 and H2AK119ub; iv) SUZ12 and H2AK119ub; and v) MTF2 and H2AK119ub. Regions overlapping H2AK119ub peaks depleted in both (red, common), shDPPA4-only (blue), shDPPA2+4-only (purple); upregulated in shDPPA4-only (light green) or shDPPA2+4-only (dark green) are highlighted.
c) Genome browser views (left) and expression (right) for DPPA2/4-dependent H2AK119Ub that are depleted of H3K27ac upon A-485 treatment for 24h. The trackplots contain averaged CPM/bp for Input (n=1), H3K27ac (n=2), H3K27me3 (n=3) and H2AK119ub (n=2) ChIPs in either control (DMSO) or A-485 10uM treated NCI-H661 cells after 24h. CpGi's are annotated above each plot (red), bollow each plot are DPPA2/4 peaks (blue), H2Aub differential region (maroon) and H3K27ac differential region (gold). The regions are; i) DLX1-DLX2 locus (chr2:172067177-172123476), ii) ITGB8 locus (chr7:20312387-20349427), iii) FOXP2 locus (chr7:114051253-114125334) and iv) CYP26A1 locus (chr10:93043900-93106103) respectively.
d) Bar plots of DLX1, DLX2, ITGB8, FOXP2 and CYP26A1 gene expression (RNA-seq) in either control (DMSO) or A-485 10uM treated NCI-H661 cells after 24h (these are all differentially down-regulated, log2FC < -1, FDR<0.05). Values are log10-transformed TPM values, error bars represent the SEM of three biological replicates.

Supplemental Figure 6, relating to Figure 6

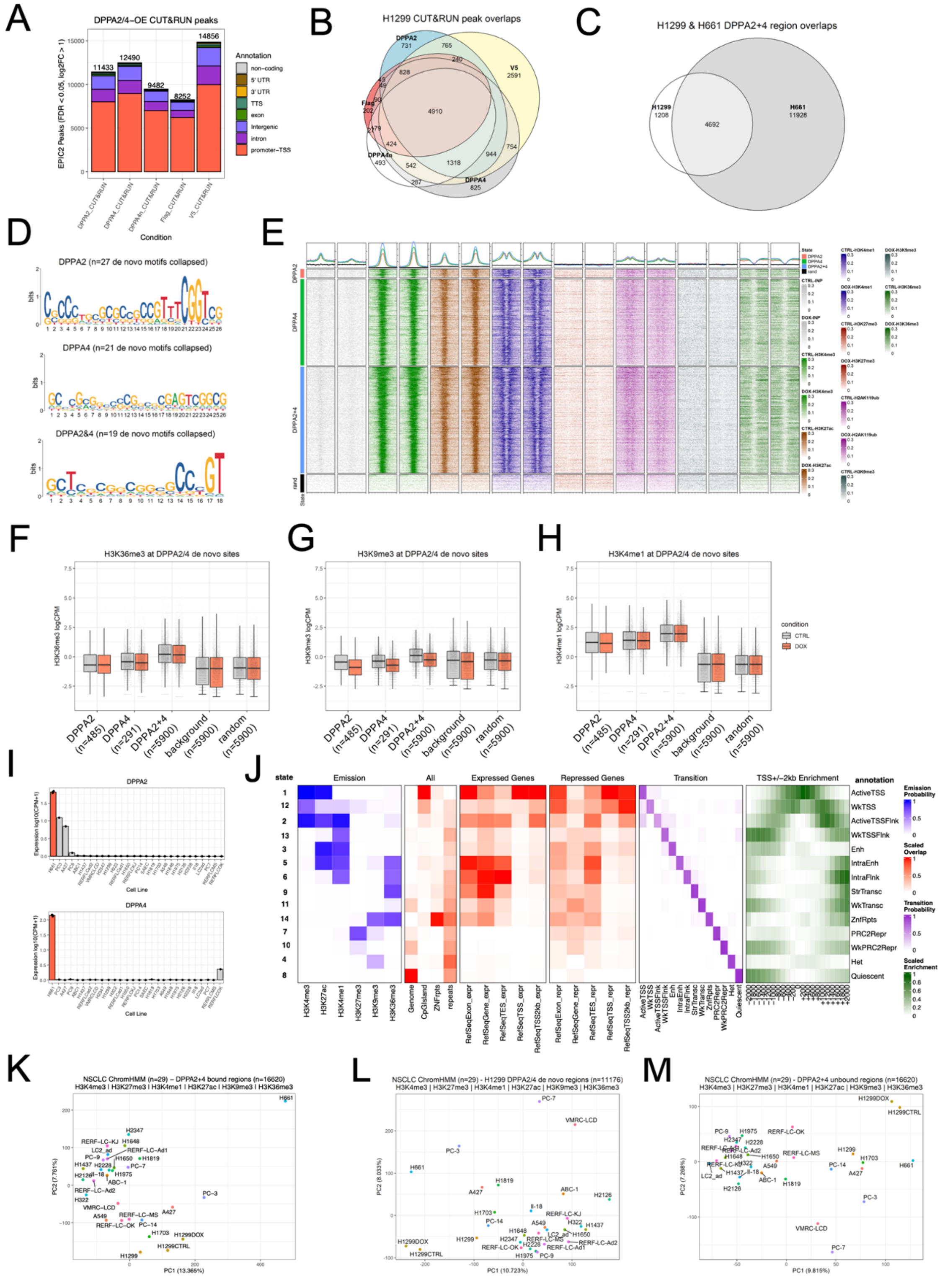

Supplemental Figure 6, related to Figure 6

a) HOMER genomic annotations of DPPA2, DPPA4, Flag-tag (DPPA2) and V5-tag (DPPA4) CUT&Run peaks in NCI-H1299 following 7 days of doxycycline treatment (determined by EPIC2, FDR < 0.05, log2FC > 1). Promoter-TSS is defined as TSS+/-1500bp. The numbers atop each

column indicates the total number of DPPA2/4 peaks (the number of overlapping peaks (>25%) between two replicates).
b) Venn diagram of overlaps for the aforementioned CUT&Run peaks.
c) Venn diagram of overlap between DPPA2/4 consensus peaks in H661 and H1299.
d) Collapsed HOMER *de novo* motifs from DPPA2-only (n=485), DPPA4-only (n=4791) and DPPA2+4 (n=5900) ChIP/CUT&Run peaks relative to a set of background genomic regions (n=5900) size and gc-content matched to the DPPA2/4-peak sets. All regions were resized to 1000bp prior to analyses.
e) Heatmaps of genomic regions centred on DPPA2/4 de novo peaks (resized to +/-5kb from the centre of each peak, split into 100 equal windows) grouped into: DPPA2-only (n=485), DPPA4-only (n=4791), DPPA2+4 (n=5900) and background genomic regions (n=1000). Columns represent Input (n=1), H3K4me3 (n=2), H3K27ac (n=2), H3K4me1 (n=2), H3K27me3 (n=2) , H2AK119ub (n=2), H3K9me3 (n=2), H3K36me3 (n=2) ChIPs in either control or A-485 treated conditions. ChIP CPM/bp values were scaled per bin across all regions for visualisation.
f-h) Boxplots of H3K6me3, H3K9me3 and H3K4me1 signal at DPPA2/4 de novo and background regions in H1299 DPPA2+4 inducible cells following 7 days of induction with control or doxycycline (averaged values between 2 replicates).
i) Bar plots showing expression of DPPA2 (top) and DPPA4 (bottom) across DBTSS cell lines used in ChromHMM modelling. NCI-H661 cells are included as a comparison
j) 14-state chromatin model as determined by ChromHMM modelling across all NSCLC lines. The first panel presents emission probabilities that summarise the association of each mark with each chromatin state. The second to fourth panel indicates the scaled overlap of each chromatin state with annotated features in the genome, note that RefSeq annotations are split according to expressed and repressed genes in NCI-H661, as determined by RNA-seq. The fifth panel indicates transition probabilities that indicate the relatedness between states. The sixth panel represents the scaled enrichment of each signal flanking the TSS+/-2kb.
k-m) PCA analysis of factorised chromatin states (ChromHMM) at **k**) H661 DPPA2/4bound regions (n=16620) **l** H1299 de novo DPPA2/4 bound regions (n=11176), and **m)** an equally sized set of DPPA2/4 unbound regions in H661 (n=16620).

Supplemental Materials & Methods

Inducible shRNA and cDNA construct generation and stable cell line generation

For inducible knockdown experiments, miR30 based shRNAs were expressed using a tetracycline-inducible *PiggyBac* transposon system (pCLIIPi-iRFP-shRNA-pGK-mVenus), as previously described(Eckersley-Maslin et al. 2020) (Eckersley-Maslin *et al*. 2020). Established shRNA sequences(Fellmann et al. 2013) against either; Renilla luciferase (non-targeting control) (#713), human DPPA2 (#760) or DPPA4 (#1993) were cloned by first annealing complementary oligo's and ligating them into a digested pMSCV-miR30 backbone(Narita et al. 2006) , followed by PCR, digestion and ligation into the pCLIIPi backbone. Stable cell lines were generated by transfecting NCI-H661 cells with the shRNA vectors along with *PiggyBac* transposase (pRM1024)(Moudgil et al. 2020) , waiting for 2 weeks, then sorting mVenus positive cells twice with expansion in between, collecting the top 50% of mVenus positive cells each time as a bulk population for further experiments. The mVenus DPPA4 shRNA construct underwent additional cloning to replace mVenus with tagBFP to allow for selection of cells containing both DPPA2 and DPPA4 inducible shRNA. To generate the double inducible knockdown line, stable shDPPA2 (mVenus+) cells were transfected with the shDPPA4 (tagBFP) vector and selected as above for both mVenus+/tagBFP+ cells. For inducible overexpression experiments the above vector backbones were cloned to replace the (iRFP-shRNA) fragment downstream of the tet-response element (TRE) with cDNA encoding human DPPA2 (ORF:NM_138815) or DPPA4 (ORF:NM_018189) with additional N-terminal Flag or V5 tags respectively (gene fragments synthesised by IDT). Stable cell lines were generated in the same way as above for shRNA lines for the NCI-H1299 cell line (which does not express DPPA2/4). All cell sorting was undertaken on FACSAria Fusion flow cytometry instruments (BD). To induce either shRNA or cDNA expression, cells were treated with 2μg/ml doxycycline (Dox) for 3-7 days and knockdown or overexpression were confirmed by RT-qPCR or western blot analysis. All vectors were sequence verified by either Sanger (shRNA constructs) or Plasmidsaurus (cDNA constructs) sequencing. shRNA sequences are in Supplemental Table 14 below.

Generation of CRISPR-Cas9 knockout cell lines

NCI-H661 cells were electroporated using the Neon™ Transfection System (ThermoFisher) with Cas9 RNP (Horizon, CAS12205) loaded with a single guide RNAs targeted to DPPA2 (Exon 6: 5'-GCGATGTTCGAGGAAACGCA-3') or DPPA4 (Exon 3: 5'-CGGTGAATCAGATTAACAGG-3') respectively. Post electroporation, cells were seeded at low density (1 cell/well in a 384-well plate) to isolate monoclonal populations. After 3 weeks in single-cell dilution, expanded populations were screened by PCR following gDNA extraction and edits identified by Sanger sequencing. Clones with an indel knockout on all alleles were expanded. To generate DPPA2/4 double knockout clones DPPA2 single KO clone c40 was subject to DPPA4 CRISPR-Cas9 KO as above, and then subcloned and genotyped as above. The generation and validation of these isogenic cell lines were undertaken by Horizon (CLPP1690). Knockouts were further validated using western blot analyses.

siRNA transfection

siRNA knockdowns for DPPA2, DPPA4, both DPPA2+4 were performed using 10uL Lipofectamine 2000 (ThermoFisher, #11668027) and 200pmol of non-targeted and DPPA2/4 targeted siRNA twice over 96 hours (first dose at day 0, second dose at day 2, collection on day 4) using SMARTpool siRNA (containing 4 siRNAs per pool) (Horizon/Dharmacon, Non-targeting; #D-001810-10-05, DPPA2; #L-018977-01-0005, DPPA4; #L-020766-01-0005). Lipofectamine 2000 and siRNA complexes were first formed with reduced-serum OptiMEM medium (Gibco) for

20 mins at room temperature, prior to addition to cells grown in complete medium. Knockdown efficiency was validated by RT-qPCR following RNA extraction. siRNA sequences are in Supplementla Table 15 below.

gDNA extraction and Whole Genome Bisulfite Library preparation

Following treatment or passaging, NCI-H661 cells were harvested, washed once with PBS, stored in RLT+ lysis buffer (Qiagen) and snap frozen. gDNA was extracted from cells using an AllPrep RNA/DNA extraction kit (Qiagen #80204) according to the manufacturer's instructions. DNA libraries were generated using the NEBNext End Prep kit (NEB E7370, E7535) and bisulfite converted using the EZ DNA Methylation Gold Kit (Zymo Research #D5005, D5006) according to the manufacturer's instructions. Libraries were amplified using KAPA HiFi Uracil+ (KK2801/2) using NEBNext Universal and Index primers, cleaned up using 0.8x Ampure beads and pooled for sequencing.

RNA extraction, cDNA synthesis and RNA-seq library preparation

Following treatment or passaging, cells were enzymatically detached, washed once with PBS and snap frozen as a pellet or stored in RLT+ lysis buffer. RNA extraction was performed on cell pellets (NEB) or lysates (Qiagen), using either the Monarch Total RNA miniprep kit (NEB #T2010S) or AllPrep RNA/DNA extraction kit (Qiagen #80204) with DNAse I treatment according to the manufacturer's instructions. 0.5-1µg of RNA was used for cDNA synthesis via reverse transcription using the LunaScript RT kit (NEB #E3010). RNA was quantified using the Qubit™ RNA High Sensitivity (HS) Assay Kit (Invitrogen) on a Qubit 3.0 fluorometer. Following RNA quantitation, 05-1µg of RNA was used as input for PolyA+ directional RNA-seq library preparation using the NEBNext Ultra II Directional RNA-seq Kit (#E7765, NEB) with the PolyA mRNA magnetic isolation module (#E7490, NEB) according to manufacturer instructions.

Whole cell protein extraction, quantification and immunoprecipitation

Snap frozen NCI-H661 cell pellets ($5x10^6$ cells) were lysed in ice-cold RIPA lysis buffer (150mM NaCl, 50mM Tris-HCl, 1% IGEPAL CA-630 (Sigma), 0.5% sodium deoxycholate, 0.1% SDS in H_2O) for 30 minutes on ice with or without sonication for 30mins. Following centrifugation at 11,000xg to pellet debris, protein from whole cell lysates (WCL) were quantified using the BioRad protein quantitation assay (Bio-Rad, #5000006) and measured on a Cytation 3 (BioTek) plate reader using known BSA serial dilutions as a standard curve at 595nm (colorimetric). 430µg of protein WCL were used for each set of immunoprecipitations and were diluted to 165uL in PBST (PBS + 0.01% Tween-20). 100µL of Protein-G Dynabeads (Thermo) were washed with Citrate Phosphate Buffer (25mM citric acid, 50mM dibasic sodium phosphate dihydrate in H_2O calibrated to pH5.0) twice followed by resuspension in 260uL of PBST. Diluted WCL were then pre-cleared to remove proteins that non-specifically bind to Protein-G Dynabeads by adding 55uL of washed beads to the diluted WCL and incubating them at 4C for 1hr on a rotator, followed by transfer of the pre-cleared eluate to a new tube. 2µg of antibodies (DPPA2 (Mouse); Sigma #MAB4356, DPPA4 (Rabbit); abcam #ab154642) or IgG's isotype controls (Mouse; Invitrogen #31903, Rabbit; Invitrogen #02-6102) diluted to 150uL in PBST were conjugated to 50uL of washed beads by incubating them together at 4C for 1hr on a rotator. Antibody-bead conjugates were washed two times with PBST and then resuspended in 55uL PBST. Immunoprecipitations were then performed by incubating 50uL of antibody-bead conjugates with 50uL of pre-cleared WCL overnight at 4C whilst rotating, the rest of the pre-cleared WCL was kept as an input control. Following immunoprecipitation, beads were washed five times with PBS on ice, and beads were snap frozen on dry ice as a pellet for eventual on-bead tryptic

digest for mass spectrometry or eluted in NuPAGE LDS sample buffer (Invitrogen, #NP0007) at 70C for 10 minutes for western blot analysis.

Mass spectrometry and analysis

Enriched proteins were digested off the beads overnight at 37C in 50uL of 2M urea containing 1mM tris(2-carboxyethyl)phosphine and 4mM 2-chloroacetamide with 0.4ug of sequencing grade trypsin (Promega, #V5280) and LysC (Wako, #125-05061). The peptides were purified using SDB-RPS microcolumns, washed with 99% isopropanol containing 1% trifluoracetic acid (TFA) followed by 5% acetonitrile containing 1% TFA and the eluted with 80% acetonitrile containing 1% ammonium hydroxide. Peptides were dried by vacuum centrifugation and resuspended in 2% acetonitrile containing 0.1% TFA. Peptides were separated on a Dionex 3500 nanoUHPLC, coupled to an Orbitrap Lumos mass spectrometer via electrospray ionization in positive mode with 1.9 kV at 275 °C and RF set to 30%. Separation is achieved on a 50 cm × 75 µm column packed with C18AQ (1.9 µm) over 40 min at a flow rate of 300 nL/min. Peptides were eluted over a linear gradient of 3–40% Buffer B (Buffer A: 0.1% v/v formic acid; Buffer B: 80% v/v acetonitrile, 0.1% v/v FA) and the column was maintained at 50°C. The instrument was operated in data-independent acquisition (DIA) mode, with an MS1 spectrum acquired over the mass range 350–1,400 m/z (60,000 resolution, 100% automatic gain control (AGC), and 45 ms maximum injection time) followed by sequential MS/MS spectra across 13.7 m/z isolation windows with 1 m/z overlap covering the full mass range. MS/MS data will be acquired with higher-energy collisional dissociation (HCD) fragmentation (15,000 resolution, 2000% AGC, 55 ms maximum injection time, and normalized collision energy 30%). Data were processed in Spectronaut 1 (v7.6.230428.55965) with default setting against the Homo sapien protein FASTA sequences in the Uniprot database and filtered to 1% FDR at the PSM, peptide and protein level.

Raw label-free quantitation values (LFQ-AUC) values were subject to down-shifted imputation, whereby missing values for a given protein were imputed with a low intensity LFQ-AUC value based on the normalised distribution of the dataset. Following imputation, LFQ-AUC values were centered for each protein by log2 transformation and subtraction of the median across all samples. To determine enrichment statistics, unpaired Student's t-tests were performed between the IP conditions (DPPA2, DPPA4) and matched control IgG (Ms, Rb) respectively, followed by p-value adjustment to account for multiple comparisons using the Benjamini-Hochberg method.

Gel electrophoresis, transfer and western blots

Following protein extraction and protein quantitation (as above), 50ug of protein were prepared with NuPAGE 4xLDS sample buffer (Invitrogen) and subsequently denatured at 85C for 5 mins. Samples were loaded into 10,12 or 17-well NuPAGE 4-12% gradient Bis-Tris gels (Invitrogen) placed within an XCell SureLock electrophoresis cell with either NuPAGE MES or MOPS running buffer (Invitrogen). 5uL of PageRuler plus prestained protein ladder (ThermoFisher) was included in a well for each blot. Gel electrophoresis was performed at 90V for 5 mins, followed by 120V for 40-50 mins at room temperature. Proteins were then transferred from the gel to a nitrocellulose membrane using the XCell II Blot Module (Invitrogen) via wet transfer in NuPAGE transfer buffer (Invitrogen) containing 10% methanol for 1hr 30V at room temperature. To confirm transfer of proteins membranes were stained with Ponceau S (Merck). Membranes were then blocked for 2-4 hr at room temperature in 5% w/v Bovine Serum Albumin (A4737) or 0.1% skim-milk w/v in TBST (20mM Tris-HCl, 150mM NaCl, 0.1% Tween-20 in H2O), followed by a single wash in TBST for 5 mins. Membranes were divided for probing of multiple proteins at distinct molecular weights. Membranes were incubated with primary antibody at varying

dilutions (1/2000 Flag (#F1804, Merck), 1/5000 V5 (#R960-25, Invitrogen), 1/1000 DPPA2 (Merck, #MAB4356), 1/1000 DPPA4 (abcepta, #AP1438a, abcepta, #AP21202c, abcam, #ab154642), 1/10000 H3K4me3 (CST,#9751), 1/10000 H3K27me3 (CST, #9733), 1/10000 H2AK119ub (CST, #8240), 1/10000 H3K27ac (abcam, #AB4729), 1/10000 total H2A (CST, #12349), 1/10000 total H3 (CST#, 9715), 1/5000 Vinculin (CST, #13901) in 0.1% skim-milk w/v in TBST overnight at 4C whilst rotating. Membranes were then washed three times in TBST for 5 mins each time, followed by incubation with HRP conjugated anti-rabbit (abcam #ab205718) or anti-mouse (abcam #ab205719) secondary antibodies at a 1:2000 dilution in 5% w/v Bovine Serum Albumin (A4737) or 0.1% skim-milk w/v in TBST for 2-4 hrs at room temperature whilst rocking. Membranes were washed three times with TBST for 5mins each, then incubated in Clarity chemiluminescent ECL substrate (Bio-Rad) for 2 mins followed by chemiluminescence visualisation on the iBright 1500 instrument (Invitrogen). When required, membranes were stripped of bound antibodies by incubation with Restore western blot stripping buffer (ThermoFisher) for 30 mins at room temperature whilst rocking, followed by TBST washes, re-blocking and probing of antibodies as above.

Immunofluorescence, image acquisition and analysis

NCI-H661 cell lines were seeded on sterilised glass coverslips placed within 6-well plates. When cells were ~70% confluent medium was aspirated, wells were washed twice with PBS, fixed in 4% formaldehyde in PBS for 10-15 mins at room temperature. Cells were washed twice with PBS and then permeabilised with 0.1% Triton-X in PBS for 5-10 minutes at room temperature. Cells were washed twice with PBS and then blocked with 3% BSA in PBS for at least 3 hours at room temperature whilst rocking. Primary antibodies (1/50 DPPA2 (Merck, #MAB4356), 1/50 DPPA4 (abcam, #ab154642)) were diluted in 3% BSA in PBS and coverslips were placed downward between parafilm for uniform staining 60-90 minutes at room temperature in a humidity chamber. Coverslips were washed 3x in PBS followed by incubation in fluorophore conjugated secondary antibodies anti-rabbit Alexa 594 (ThermoFisher, #A11037) or anti-mouse Alexa 488 (ThermoFisher #A32723) at 1/1000 in 3% BSA in PBS for 1hr at room temperature. Following 3 washes in PBS slides were counterstained with DAPI (ThermoFisher, #62248) at 1/10,000 in PBS for 1min at room temperature. Slides were mounted onto SuperFrost microscope slides with SlowFade Gold antifade (Invitrogen) and sealed with nail polish. Slides were imaged on an Olympus FV3000 confocal microscope. Image analysis was performed using ImageJ.

Flow cytometry analysis and cell sorting

Cells were resuspended in FACS buffer (2% FBS, 4mM EDTA in PBS) prior to flow cytometry analysis using the LSR II (BD) flow cytometer or sorted using the FACSAria Fusion flow cytometer (BD).

Rapid immunoprecipitation mass spectrometry (RIME) and analysis

Chromatin immunoprecipitation and sonication were carried out exactly as described for double-crosslinking ChIP experiments above with 3μg of antibodies (DPPA2 (Mouse); Sigma #MAB4356, DPPA4 (Rabbit); Abcepta #AP1438a) or IgG's isotype controls (Mouse; Invitrogen #31903, Rabbit; Invitrogen #02-6102). Following immunoprecipitation with antibody coupled protein A dynabeads, washes were performed as previously described(Mohammed et al. 2016), with the final wash in cold 100 mM Triethylammonium bicarbonate (TEAB). On-bead tryptic digests were then performed by incubating beads with 1ug trypsin (Thermofisher) in 50mM acetic acid overnight at 37C. A second tryptic digest was performed by adding another 0.25ug trypsin for 4 hours at 37C. A final acidic extraction was performed by incubating beads in 10%

formic acid at 37C and pooling all digests together. Peptides were dried by vacuum centrifugation and resuspended in 2% acetonitrile containing 0.05% TFA.

All data were acquired using a Thermo Scientific Vanquish Neo UHPLC system coupled to an Orbitrap Astral mass spectrometer. Peptides were injected onto an Acclaim Pepmap nanotrap enrichment column (C18, 100 Å, 75 µm × 2 cm) at a flow of 2.5 µL/min of 3% (v/v) ACN containing 0.1% (v/v) formic acid. The sampler module was configured for fast loading with a desired loading flow rate of 20 µl/min and a maximum loading pressure of 800 bar. The system supported both weak (0.1% Formic acid) and strong (80% Acetonitrile, ACN) washing solutions, with the sampler performing two wash cycles with automatic trap wash cycle adjustments was utilized. The pump module was set for fast column equilibration with a flow rate of 2.5 µl/min, operating under a combined control mode for solvent delivery. The enrichment column was then switched in line with the High Throughput µPAC™ Neo HPLC Column (C18, 100-300 Å, 75 µm × 5.5 cm) at a flow rate of 0.750 µl/min. The eluents were 100% (v/v) water in 0.1% (v/v) formic acid (solvent A) and 80% (v/v) ACN and 0.1% (v/v) formic acid (solvent B). The flow gradient was (i) 0.0–0.3 min: 3–6% B,ii) 0.3–9.0 min: 6–23.5% B, iii) 9.0–10.7 min: 23.5–40% B, iv) 10.7–11.7 min: 40–50% B, v) 11.7–11.8 min: 50–99% B, vi) 11.8–13.0 min: 99% B, vii) Post 13.0 min, quickly return to starting conditions for column equilibration. Peptides eluting from the column were transferred to the gas-phase by electrospray ionisation operating in the positive ion mode. The spray voltage was 1.9 kV and the ion transfer tube temperature was 290 °C. Mass spectrometric data were acquired in Data-Dependent Acquisition (DDA) mode with a total method duration of 13.7 minutes. Full MS1 scans were acquired in the Orbitrap detector at a resolution of 120,000, over a mass range of 380–980 m/z, in profile mode. The normalized AGC target was set to 300%, with a custom injection time of 50 ms, 1 microscan, and an RF lens setting of 40%. MS/MS spectra were acquired in the Astral analyzer using data-dependent ddMSn scans. The precursor isolation window was 0.7 m/z, and normalized HCD collision energy was set at 27%. Fragment ion spectra were acquired from 150–2000 m/z in centroid mode, with a normalized AGC target of 300%, injection time of 6 ms, and 1 microscan. Dynamic exclusion was enabled to prevent repeated fragmentation of the same precursor for 20 seconds after a single occurrence. Only precursors with charge states 2–6 and a minimum intensity of 5,000 were selected for MS/MS. All scans were performed in positive ion mode, and Advanced Peak Determination (APD) was enabled to enhance precursor selection.

Data were processed using FragPipe(Hsiao et al. 2024) (v23.1) (MSFragger v4.3(Yu et al. 2025; Kong et al. 2017; Teo et al. 2021), IonQuant(Yu et al. 2021) v1.11.11, diaTracer v1.3.3, DIA-NN v1.8.2b8 and Python v3.11) using the default label-free quantitation with match between runs normalisation (LFQ-MBR) workflow (with MS-Fragger in DDA+ mode and MaxLFQ enabled for IonQuant). Output LFQ-MBR values were used to determine enrichment statistics, unpaired Student's t-tests were performed between the IP conditions (DPPA2, DPPA4) across several clones (WT/DKO) and matched isotype controls (Ms, Rb IgG), followed by p-value adjustment to account for multiple comparisons using the Benjamini-Hochberg method. We define enrichment as the protein having a log2FC>1 (adjusted p-value < 0.05) enrichment over both respective matched isotype controls and DKO IPs for both clones.

Assay for transposase accessible chromatin (ATAC-seq) and library preparation

NCI-H661 cells were harvested and counted after staining cells with 0.4% Trypan blue where 500,000 live cells were used for each ATAC assay. Cells were washed with ice cold PBS once and then lysed in 500uL NE buffer (10mM Tris-HCl, 10mM NaCl, 3mM $MgCl_2$, 0.1% IGEPAL CA-630, 0.1% Tween-20) on ice by gentle pipetting to isolate nuclei. 50uL of nuclei (50,000 cells), were then pelleted at 1500xg for 10mins at 4C followed by supernatant aspiration. Nuclei were gently resuspended directly in tagmentation mix (1.75uL Tn5 transposase (Illumina), 17uL TD

buffer (Illumina), 21.25uL H_2O) on ice and then allowed to tagment for 37C for 30 mins in a thermalcycler. Tagmented DNA was then purified using Ampure XP beads at a 1.8X ratio and eluted in 21uL of H_2O for library preparation as previously described using 15 cycles of PCR to incorporate i7/i5 indices followed by a final 1.8X Ampure XP bead cleanup and elution in 22uL of H_2O.

DNA library preparation and sequencing

For ChIP, reChIP and CUT&Run libraries were prepared using the NEBNext UltraII DNA Library Preparation Kit (NEB) according to the manufacturer's instruction. During library preparation the NEBNext Multiplex Oligos for Illumina (Index Primers Set 1-4) were used to enable library multiplexing. Individual and pooled libraries were quantified and quality controlled using the dsDNA high sensitivity (HS) Qubit assay (Invitrogen) on the Qubit 3.0 fluorometer (Invitrogen) and HSD1000 High Sensitivity DNA kit (Agilent) on the TapeStation 4150 (Agilent). Libraries were pooled and sequenced on the Illumina NextSeq500 or NextSeq2000 platform in either single-end (RNA-seq, ChIP-seq) or paired-end (ATAC-seq, CUT&Run) configurations with 75-150bp read lengths targeting 15-30x10^6 75-100bp single-end (ChIP, reChIP, RNA-seq), 10-15x10^6 100bp paired-end (CUT&Run), 50x10^6 100bp paired-end (ATAC-seq) or 100x10^6 150bp paired-end (WGBS) reads.

Nucleosome reconstitution

Nucleosomes were reconstituted as described previously(Tan et al. 2020). DNA used to generate nucleosomes was the 147 bp Widom 601 sequence(Lowary and Widom 1998) prepared by annealing complementary single stranded 147 bp Ultramers™ (Integrated DNA Technologies) with the sequence 5'-
ATCGAGAATCCCGGTGCCGAGGCCGCTCAATTGGTCGTAGACAGCTCTAGCA
CCGCTTAAACGCACGTACGCGCTGTCCCCCGCGTTTTAACCGCCAAGGGGATTACTCCCTAGTCT
CCAGGCACGTGTCAGATATATACATCCGAT-3'. To prepare nucleosomes, 10 μM recombinant human histone octamers (histonesource.com) were buffer exchanged into buffer A (2 M NaCl, 10 mM Tris-HCl pH 7.5, 1 mM EDTA, 5 mM DTT) using a 7 kDa MWCO Zeba desalting column (Thermo Fisher), combined at a 1:1.2 molar ratio with Widom 601 dsDNA in buffer A and incubated for 30 min at 4 °C. The reconstitution reaction was dialyzed in buffer A with decreasing salt concentration stepwise from 2.0 M, 1.0 M, 0.8 M, 0.6 M to 0.2 M NaCl with incubation time of 2–3 hrs with 0.8 M NaCl buffer dialysis being an overnight step using a 7 kDa MWCO Slide-A-Lyzer mini dialysis unit (Life Technologies). The nucleosome reconstitution was confirmed by electromobility shift assay using 6% native acrylamide gel, visualized with ethidium bromide and Coomassie blue. The reconstituted nucleosomes were stored at 4 °C and used within 4 weeks of production.

Electrophoretic mobility shift assay (EMSA)

For nucleosome-binding assays, 50nM of recombinant nucleosomes were incubated in assay buffer (20 mM Tris-HCl pH 7.4, 75 mM NaCl, 6% glycerol 0.005% NP-40, 0.5 mM $MgCl_2$) with 0, 125, 250, 500 or 1000 nM recombinant DPPA2-Myc/Flag (Origene, TP305441), His-DPPA4 (Origene, TP760278) or DPPA2-DPPA4 complex (mixed in equi-molar concentration) in 10 μL reactions. The reactions were performed at room temperature for 30 min and resolved in a 0.5x TBE 6% 37.5:1 acrylamide: bis-acrylamide gel for 120 min at 100 volts at room temperature. A DNA ladder (GeneRuler 50bp; Thermo Fisher) was included in the gels. The gel was stained with SYBR Gold (Thermo Fisher) and imaged on ChemiDoc (BioRad). Each assay was repeated three times. The bands were quantified with ImageJ (ver2.14.0). Percentage of shifted nucleosomes is

calculated by measuring the intensities of bottom bands and fraction of loss of bands against the nucleosomes only band.

Mass photometry

Mass photometry experiments were performed on Refeyn TwoMP mass photometer (Refeyn). Microscope coverslips (Refeyn) and silicon well gaskets (Refeyn) were cleaned with 100% isopropanol and water and dried with compressed air. The gaskets were placed on top of the cleaned coverslips on the sample stage of the mass photometer as per manufacturer's instructions. All measurements were performed at least three times independently, in separate wells, in buffer containing 20 mM HEPES pH 8, 50 mM NaCl, 1 mM TCEP. Firstly, 10 µL buffer was pipetted into a gasket-well followed by focal point acquisition using the autofocus functionality in the Refeyn AcquireMP 2.3.1 software. Secondly, 10 µL of 100 nM of recombinant DPPA2-Myc/Flag (Origene, TP305441), His-DPPA4 (Origene, TP760278) or in complex (mixed in equi-molar concentration and diluted to 100nM just prior to measurement to avoid complex disassembly) was pipetted in the buffer-containing gasket-well and mixed to observe individual landing events below saturation levels. Mass photometry movies of 6000 frames were recorded from a 10.8 × 10.8 µm instrument field of view. Data were processed using the default pipeline on the Refeyn DiscoverMP 2.3.0 software. Individual particle contrasts from each video were converted to mass using a contrast-to-mass (C2M) calibration which was performed in the acquisition buffer. Data were plotted as normalised histograms with a bin width of 4.4 and fitted to a Gaussian peak. For the calibration, 10 µL of a 1:30 pre-diluted Glutamine Synthetase (CLENZ544-2, Bio-Scientific) was added to an acquisition gasket-well; the 57, 231, 460 kDa masses were used for a standard calibration curve in the DiscoverMP software. The experiments were repeated three times independently.

Quantitative PCR

qPCR analysis of purified ChIP DNA and RT cDNA were performed in technical duplicate for each primer pair using 2x SYBR mastermix (Applied Biosystems, #4385612) according to manufacturer's instructions in a 6-12uL reaction on CFX96 or CFX384 (Bio-Rad) instruments. Primer sequences are in Supplemental Table 16 below.

TCGA, ERM and GTEx multiomic analyses

Clinical information as well as matched transcriptome (RNA-seq), DNA methylation (methylation array), copy number and mutation (WES) data were obtained from the static LinkedOmics data repository for 11,518 cancer patients spanning 32 cancer types(Vasaikar et al. 2017) from the cancer genome atlas (TCGA) study. RNA-seq untransformed gene level read counts were also obtained for NSCLC (LUAD and LUSC) patients from the genomic data commons (GDC) database. Processed TCGA pan-cancer chromatin accessibility (ATAC-seq) data(Corces et al. 2018) were also obtained from the GDC database. For mutations (WES), any mutation (SNV, deletion, insertion) on the DPPA2/4 gene body was considered. For copy number (WES) we classified ≥ 3 gene copies as a gain, ≤ 1 copies as a loss, and 2 copies as diploid. For ATAC-seq we utilised pre-compiled pan-cancer peaks and normalised counts at these peak regions for DPPA2/4 gene promoters (DPPA2p1 (TGCT_15780), DPPA4p1 (TGCT_15782), DPPA4p2 (TGCT_15783), DPPA4p3 (TGCT_15784)) or putative enhancers (DPPA4e1 (TGCT_15785), DPPA4e2 (TGCT_15786))[8]. Cox proportional hazard modelling (CoxPH) of patient outcomes (overall survival) were performed using the survival (v3.2-14) R package, where patients in each cohort was subdivided by the co-expression of DPPA2 and/or DPPA4 as determined by RNA-seq (expression cut-off defined as the median of all DPPA2 or DPPA4 expressors (RSEM>0)). Kaplan-Meier overall survival plots of patient groups were done

using the survminer (v0.4.9) R package. Correlations between DPPA2/4 gene expression and copy number, promoter methylation and chromatin accessibility were assessed using Pearson correlation with the stats (v4.1.2) R package whereas correlations with binary mutation calls per patient were done using biserial correlation with the polyserial (v0.8-1) R package, dot plots visualisation these correlations were plotted using the corrplot (v0.92) R package. To discern differentially expressed gene in DPPA2/4 co-expressing NSCLC (LUAD, LUSC) tumours compared with DPPA2/4 non-expressing tumours we used untransformed read counts and performed differential gene expression analysis using the edgeR (v3.36.0) R package using the glmFit/LRT regression workflow whilst accounting for tumour subtypes in the model design (~Subtype+Group), where differentially expressed genes were considered as having an absolute log2FoldChange ≥ 1 and FDR < 0.05. All gene were then ranked by decreasing log2FC and then input to a gene set enrichment analysis (GSEA) using the fgsea (v1.20.0) R package against molecular signatures database (MSigDb) pathways with particular focus on the C2 and C8 pathway subsets. Processed gene expression (RNA-seq) data for n=19616 normal adult tissue samples across 30 tissue types were obtained from the Genotype-Tissue Expression (GTEx) database. Gene expression (RNA-seq) and DNA methylation (WGBS) data for human cell lines established across development were obtained from the Epigenetic Roadmap (ERM) (https://egg2.wustl.edu/roadmap/web_portal/). WGBS ERM data in bigwig format were imported into R using the rtracklayer package (v1.54.0). Since data were originally aligned to the hg19 human genome reference, genomic coordinates in these files were lifted-over using the liftOver R package (v1.18.0) with the hg19toHg38 UCSC liftover chain file (https://hgdownload.cse.ucsc.edu/goldenpath/hg19/liftOver/). Processed data were averaged over the DPPA2/4 promoter (TSS+/-1500bp) using the EnrichedHeatmap (v1.24.0) R package (normalizeToMatrix). All boxplots, barplots and scatter plots were plotted using the ggplot2 (v3.4.4) R package.

NGS data pre-processing

Single-end and paired-end reads in fastqs were trimmed and filtered for quality (phred33 score > 20) and length (>20bp) using TrimGalore (https://github.com/FelixKrueger/TrimGalore) v0.6.6 in single-end or paired-end mode (valid read pairs were only kept). Trimmed and quality filtered reads were then aligned to the hg38 human genome GRCh38.p13 (and for spike-in ChIP were also aligned to the mm10 mouse genome GRCm38.p6 separately) using bwa-mem(Li and Durbin 2009) (bwa v0.7.13) with default parameters for ChIP-seq, CUT&Run, WGBS and ATAC-seq data. For ChIP-seq, CUT&Run and ATAC-seq, alignments were then converted to the bam format and indexed using samtools v1.9(Li et al. 2009) . Duplicate alignments were marked with using MarkDuplicates (picard v2.6.0, https://broadinstitute.github.io/picard/) and re-indexed with samtools. For ATAC-seq data we additionally shifted reads by -4bp on the + strand and +5 bp on the -strand to account for Tn5 insertions using alignmentSieve (DeepTools v3.5.0)(Ramírez et al. 2014) . For spike-in ChIP data, we used the xenofilteR (v1.6)(Kluin et al. 2018) R package to split human and mouse aligned reads. bigWig files containing CPM/bp normalised coverage values for each sample were derived from duplicate marked bam files using bamCoverage (DeepTools v3.5.0) where single-end data were extended to 147bp and paired-end data were not extended (relying instead on the true fragment length determined by read-pairs).

For WGBS data following read trimming as above, Bismark (v0.22.3)(Krueger and Andrews 2011) was used to align reads to a bisulfite converted hg38 (GRCh38.p13) genome using bowtie2 internally, followed by read deduplication and methylated/unmethylated read counting. The resultant methylation bedgraph was converted to the bigwig format using bedGraphToBigWig (UCSC.utils (v1.3.1) R package).

For RNA-seq data following read trimming as above, reads were aligned using the splice-aware aligner STAR (v2.7.5b)(Dobin et al. 2012) and gene counts were made using featureCounts (subread v2.0.6)(Liao et al. 2013) with human GENCODE v36 gene annotations.

Peak calling, annotation, differential testing (ChIP-seq, reChIP-seq, CUT&Run, ATAC-seq)

Peak calling for histone modification ChIPs (H3K4me3, H3K4me1, H3K27ac, H3K27me3, H2AK119ub, H3K9me3, H3K36me3 and H3K36me3) as well as reChIPs were performed separately for independent replicates using EPIC2 (v0.0.52)(Stovner and Sætrom 2019) (--bin-size 100 –gaps-allowed 1 –fragment-size 147 -- false-discovery-rate-cutoff 0.05) using matched genomic inputs as the control. For DPPA2, DPPA4, SUZ12 ChIP we used MACS2(Zhang et al. 2008) (v2.2.7.1) to call narrow peaks, using matched genomic inputs as the control (-B --nomodel --extsize 147 --SPMR -q 0.05). For CUT&Run and ATAC-seq we also used MACS2 to call peaks, except with options more suitable for paired-end data (-f BAMPE -q 0.05) using matched IgG's as the control for CUT&Run and no controls for ATAC-seq. Downstream data analyses were conducted using R (v4.1.2).

Peaks were filtered (log2FC >1 and FDR < 0.05) and regions overlapping blacklisted regions from the hg38 ENCODE blacklist were excluded(Amemiya et al. 2019) . To derive commonly enriched regions between replicates we retained and merged peaks with >25% overlap in replicates using the GenomicRanges R package (findOverlaps, Reduce and union) (v1.46.1). For reChIP analyses H3K4me3-H2AK119ub and H2AK119ub-H3K4me3 peaks were also merged as above to derive reciprocally bivalent regions. All consensus peak sets following merging were annotated using HOMER (annotatePeaks.pl) (v4.11)(Heinz et al. 2010) which by default denotes promoter-TSS regions as 1kb upstream and 100bp downstream of transcription start sites (TSS). Promoter-TSS regions were re-defined as the region spanning 1.5kb upstream and 1.5kb downstream of TSS's.

For differential enrichment analyses a consensus peak set was first derived using the GenomicRanges R package (Reduce and union) where atleast >50% overlap between peaks of replicates had to be observed, form which a final union was taken. Reads were counted at consensus peaks using the csaw R package(Lun and Smyth 2016) (regionCounts) (v1.28.0). Normalisation factors for each sample were pre-computed by binning the genome into 10kb windows, generating counts for each 10kb window and performing trimmed mean of M-values (TMM) normalisation using csaw (normFactors) to adjust for compositional biases. Differential enrichment was then performed using edgeR (estimateDisp>glmFit>glmLRT) (v3.36.0) in which doxycycline conditions were contrasted with their matched vehicle control per cell line. Since experiments were replicated in complete batches we accounted for this batch-effect in design models for differential testing (~Batch+Treatment). Benjamini-Hochberg p-value corrections for multiple testing were applied to these contrasts. Significant differential enrichment was defined as a region that had an absolute log2FC ≥ 0.5 and FDR < 0.05.

CpG island and ENCODE cis-regulatory element overlaps were performed using the GenomicRanges R package (findOverlaps) and were obtained for the hg38 genome using the UCSC table browser. Odds ratio testing to test for representation of sets of regions were performed using the fmsb (v0.7.3) R package, of which resultant p-values were corrected for multiple comparisons using the Benjamini-Hochberg method.

Differential gene expression analysis (RNA-seq)

Differential testing for RNA-seq data were conducted on unstransformed gene counts (filtered for all expressed genes (>1 count in any sample) using the edgeR R package

(estimateDisp>glmFit>glmLRT). For siRNA knockdown and shRNA knockdown comparisons data were generated in complete batches we accounted for this batch-effect in design models for differential testing (~Batch+Treatment) relative to a treatment control. For shRNA knockdowns we noted a prominent doxycycline-induced transcriptional program in the above comparison that was later accounted for using an additional design model (~shRNA+Treatment+shRNA:Treatment) using the shREN (control shRNA) condition to model the global effect of doxycycline on transcription. For principal component analyses we utilised batch-corrected counts from edgeR (removeBatchEffect).For all comparisons differentially expressed genes were considered as having an absolute log2FoldChange ≥ 1 and FDR < 0.05. All boxplots, barplots and scatter plots were plotted using the ggplot2(Valero-Mora 2010) (v3.4.4) R package.

Differential methylation analysis (WGBS)

Bismark coverage files reporting per-CpG methylation counts were imported to R for use in differential methylation analyses with edgeR (readBismark2DGE). We retained those CpGs that had a read count coverage of atleast 10 in all samples and that were not solely hypomethylated or hypermethylated for differential testing. We then performed differential testing between treatments and controls, accounted for batch-effects in the design model (~Batch+Treatment) using a modified design matrix that accounts for both methylated and unmethylated counts (modelMatrixMeth) using edgeR (estimateDisp>glmFit>glmLRT). Differentially methylated CpGs were those with an FDR < 0.05. Scatter plots were plotted using the ggplot2 (v3.4.4) R package

Genomic enrichment heatmaps and trackplots (ChIP-seq, reChIP-seq, CUT&Run, WGBS)

To generate genomic enrichment heatmaps and trackplots bigWigs containing CPM/bp normalised read densities were imported to R using the rtracklayer package (import.bw) (v1.54.0)(Lawrence et al. 2009) . For heatmaps, each peak region was first extended to 5kb upstream and downstream and then split into 100 equally sized bins using the GenomicRanges R package (resize) (v1.46.1). The average CPM/bp was calculated for each bin for each ChIP using the EnrichedHeatmap R package (normalizeToMatrix) (v1.24.0). Bins with values surpassing the 99th percentile of all bins within each ChIP were masked (i.e. assigned the 99th percentile value) to eliminate extreme outliers from affecting colour scales. Each bin was then scaled relative to the highest value (so values range between 0-1 and represent the relative enrichment of signal across all regions), except for ChIPs where treatments were performed (e.g. vehicle control and doxycycline) which were left unscaled and WGBS data which were already between 0-1 (methylation fraction) and did not need scaling. Enriched heatmaps were then plotted using the same package (EnrichedHeatmap), with the average bin value plotted as continuous curves atop each heatmap. Genomic track plots were plotted using the rtracklayer (v1.54.0) and Gviz R (Yu et al. 2012) packages (v1.38.4) with no prior scaling and represent either CPM/bp or methylation fractions. CpG island annotations and ENCODE cis-regulatory elements for the hg38 genome were retrieved from the UCSC table browser.

Gene ontology

The enrichment of gene ontologies across subclasses of differentially expressed or enriched genes were determined using the clusterProfiler R package (v4.2.2). Gene symbols were first converted to entrez id's using the biomaRt(Smedley et al. 2009) R package (v2.50.3) and were input alongside a background list of all expressed genes to clusterProfiler (compareCluster) against the Gene Ontology (GO) Biological Processes (BP) database. Significantly enriched GO terms were those with a Benjamini-Hochberg (BH) corrected p-value < 0.05, had atleast 10

genes present in the pathway and a gene ratio (genes in subclass/genes in pathway) > 0.01. Representative pathways were plotted using the ggplot2 R package (v3.3.5).

CG-content and motif analysis

For all sequence based analyses, randomised sequences were modelled off the width and re-sampled based on the GC-content of peak regions of interest (made with the regioneR R package (createRandomRegions)(Gel et al. 2016) (v1.26.1) and nullranges R package (matchRanges R package(Davis et al. 2023) , providing GC-content as a covariate) (v1.0.1)) and were used as the background for downstream analyses. CG content was determined for different region sets as well as background sequences above using the Biostrings R package (oligonucleotideFrequency) (v2.62.0) by first calculating all oligonucleotide frequencies and then by summing C and G frequencies. All dinucleotide frequencies were calculated using monaLisa(Machlab et al. 2022) (plotBinDiagnostics) and then GC/CG dinucleotide frequencies were summed. These data were plotted using ggplot2, and the significance of comparisons were determined using pairwise t-tests followed by BH-adjustments of p-values to account for multiple comparisons.

De novo motif discovery was undertaken for DPPA2+4 consensus binding regions following resizing of these regions as well as matched background regions to 1kb (median length of regions) using HOMER (v4.11, findMotifsGenome.pl).

Enrichments for known transcription factor binding motifs in differential peak subclasses were calculated using the monaLisa R package (v1.0.0) following resizing of all regions to 4kb (median length of all differential/background regions). Position weight matrices for transcription factor binding sites in vertebrates were retrieved from the JASPAR2020 database. Binned motif enrichment for region sets were then conducted in monaLisa (calcBinnedMotifEnrR) where significant enrichments were those with a BH-adjusted p-value < 0.05 and log2 Fold Change over background sequences > 0.25. Motif heatmaps were also plotted using monaLisa (plotMotifHeatmaps).

Chromatin state discovery (ChromHMM)

H3K4me3, H3K4me1, H3K27ac, H3K27me3, H2AK119ub, H3K9me3, H3K36me2 and H3K36me3 ChIP bam files were first converted to the bed format using bedtools (bamtobed) (v2.27.1). bed files were then partitioned into 200bp bins and then binarized for the determination of bin-specific enrichments (input as the control) using ChromHMM (BinarizeBed) (v1.24)(Ernst and Kellis 2012) . To incorporate WGBS DNA methylation data into the model we computed methylation fractions in same bins using Bismark coverage files imported via the bsseq (v1.30.0) R package, and binarized bins such that enrichment was indicated as >0.5 methylation fraction. Hidden Markov Models were then used to discover chromatin states across these genomic bins using ChromHMM (LearnModel) using a 17-state model. Segment bed files containing chromatin state annotations were then overlapped with DPPA2+4 consensus peaks, where each peak was then re-assigned to the chromatin state with the highest degree of overlap using the GenomicRanges R package (findOverlaps and pintersect) (v1.46.1). Heatmaps containing emission probabilities, transition probabilities, TSS enrichments and annotation overlaps from the ChromHMM model were then plotted using the ComplexHeatmap R package (v2.10.0).

For the integrative ChromHMM model with 27 NSCLC cell lines we performed a joint 14-state ChromHMM model for those marks which were available via DBTSS(Suzuki et al. 2015) (H3K4me3, H3K4me1, H3K27ac, H3K27me3, H3K9me3, and H3K36me3) without DNA

methylation. To summarise chromatin states at sets of regions, we called the most frequent ChromHMM state annotation per region. Transitioning states were those defined as being in atleast 75% of all other NSCLC cell lines (n=26) for a given DPPA2+4 binding site and were different from the NCI-H661 chromatin state at that site. For NCI-H1299 overexpression models we defined transitioning states as those changing from the CTRL to DOX condition.

Software

Plots were generated using R (v4.1.2/RStudio v2022.02.0+443) and edited in Inkscape. Schematic figures were made with BioRender.com with publishing licence agreement number VS290B18Q4.

Supplemental Tables

Supplemental Table 14: shRNA oligo sequences

Name	Forward	Reverse
REN.713	tcgagaaggtatatTGCTGTTGACAGTGAGCGCAGGAATTATAATGCTTATCTATAGTGAAGCCACAGATGTATAGATAAGCATTATAATTCCTATGCCTACTGCCTCGG	aattCCGAGGCAGTAGGCATAGGAATTATAATGCTTATCTATACATCTGTGGCTTCACTATAGATAAGCATTATAATTCCTGCGCTCACTGTCAACAGCAatataccttc
DPPA2.760	tcgagaaggtatatTGCTGTTGACAGTGAGCGAACCAATACAGTTGAAGTGATATAGTGAAGCCACAGATGTATATCACTTCAACTGTATTGGTCTGCCTACTGCCTCGG	aattCCGAGGCAGTAGGCAGACCAATACAGTTGAAGTGATATACATCTGTGGCTTCACTATATCACTTCAACTGTATTGGTTCGCTCACTGTCAACAGCAatataccttc
DPPA4.1993	tcgagaaggtatatTGCTGTTGACAGTGAGCGCTAAGATGTGTATGTAAAATAATAGTGAAGCCACAGATGTATTATTTTACATACACATCTTATTGCCTACTGCCTCGG	aattCCGAGGCAGTAGGCAATAAGATGTGTATGTAAAATAATACATCTGTGGCTTCACTATTATTTTACATACACATCTTAGCGCTCACTGTCAACAGCAatataccttc

Supplemental Table 15: siRNA sequences

SMARTpool	Individual siRNA in pool	Target sequence (5'-3')
ON-TARGETplus Non-targeting Control Pool<br><br>D-001810-10-05	ON-TARGETplus Non-targeting #1<br><br>D-001810-01	UGGUUUACAUGUCGACUAA

	ON-TARGETplus Non-targeting #2<br><br>D-001810-02	UGGUUUACAUGUUGUGUGA
	ON-TARGETplus Non-targeting #3<br><br>D-001810-03	UGGUUUACAUGUUUUCUGA
	ON-TARGETplus Non-targeting #4<br><br>D-001810-04	UGGUUUACAUGUUUUCCUA
ON-TARGETplus Human DPPA2 (151871) siRNA SMARTpool<br><br>L-018977-01-0005	ON-TARGETplus SMARTpool DPPA2<br><br>J-018977-09	CAGUUAAAGAUGACGCAAA
	ON-TARGETplus SMARTpool DPPA2<br><br>J-018977-10	CAAAUGGAACCAAGCGUUU
	ON-TARGETplus SMARTpool DPPA2<br><br>J-018977-11	CGGGACACUUUGCGGGACU
	ON-TARGETplus SMARTpool DPPA2<br><br>J-018977-12	CGACUGUGCUAAGAGGAAU
ON-TARGETplus Human DPPA4 (55211) siRNA SMARTpool<br><br>L-020766-01-0005	ON-TARGETplus SMARTpool DPPA4<br><br>J-020766-09	CCACAGAGAAGUCGAGGGA
	ON-TARGETplus SMARTpool DPPA4<br><br>J-020766-10	GGUGUGUGGUCCAUGGGAA
	ON-TARGETplus SMARTpool DPPA4<br><br>J-020766-11	CCGAUUCUCCAUAAAUAAA

	ON-TARGETplus SMARTpool DPPA4<br><br>J-020766-12	GUAAAGUGCUCUGCCCUAA

Supplemental Table 16: primers sequences for qPCR and genotyping. Note that the DPPA2/4 qPCR primers were also used for KO genotyping.

Target	Forward primer (5’-3’)	Reverse primer (5’-3’)
DPPA2	AGCAATTCTTGCCCATGATGC	CGATGTTCGAGGAAACGCAA
DPPA4	GAGCTCAGCTTCAATTGTTGGC	GCAGAGCACACTGACAACCC
RPL19	CGAATGCCAGAGAAGGTCAC	CCATGAGAATCCGCTTGTTT
GAPDH	TGCACCACCAACTGCTTAGC	GGCATGGACTGTGGTCATGAG

Appendix 1: ORFs:

StrepII-Flag-DPPA2 fragment (NM_138815):

TCGAAAGGATCCTTAATACGACTCACTATAGGGAGACCCAAGCTGGCTAGCCACCATGGATTATAA
AGATGATGATGATAAAGGGTCGGCCGCCAGCTGGAGCCACCCTCAGTTCGAGAAGGGAGGAGGA
AGCGGCGGAGGCAGCGGAGGAGGAAGCTGGAGCCACCCGCAGTTCGAGAAAGGAGCTAGATC
AGAGAACCTGTACTTCCAATCCATGTCAGATGCAAATTTGGATAGCAGCAAGAAGAATTTCTTGGAG
GGGGAAGTAGATGATGAGGAAAGTGTGATTTTGACACTGGTGCCAGTTAAAGATGACGCAAATATG
GAACAAATGGAACCAAGCGTTTCTTCAACTTCTGATGTCAAACTGGAGAAGCCTAAGAAATACAATC
CAGGTCATCTACTTCAAACAAATGAGCAATTTACAGCTCCACAAAAAGCTAGATGCAAAATACCAGC
CCTTCCCTTGCCGACCATTTTGCCTCCCATTAATAAGGTGTGTCGGGACACTTTGCGGGACTGGTG
TCAACAACTCGGTTTGAGTACTAATGGCAAGAAAATCGAAGTTTATCTGAGGCTTCATAGGCATGCTT
ACCCTGAACAACGGCAAGATATGCCTGAAATGTCACAAGAGACCAGATTACAGCGATGTTCGAGG
AAACGCAAGGCAGTGACCAAGAGAGCAAGGCTTCAGAGAAGTTATGAGATGAATGAGAGAGCAGA
AGAGACCAATACAGTTGAAGTGATAACTTCAGCACCGGGAGCCATGTTGGCATCATGGGCAAGAAT
TGCTGCAAGAGCTGTTCAGCCTAAGGCTTTGAATTCATGTTCCATTCCTGTTTCTGTTGAGGCCTTTTT
GATGCAAGCCTCTGGCGTCAGGTGGTGTGTGGTCCATGGCAGACTTCTCTCGGCAGACACAAAGG
GTTGGGTACGCCTGCAGTTTCATGCAGGTCAGGCCTGGGTGCCTACCACTCACAGGAGGATGATT
TCTCTCTTCTTGTTACCTGCCTGCATTTTCCCATCCCCAGGCATAGAAGATAATATGTTATGCCCCGA
CTGTGCTAAGAGGAATAAGAAGATGATGAAAAGATTAATGACAGTAGAGAAGTAAGGCGCGGCCGG
CCAGCCG

StrepII-V5-DPPA4 fragment (NM_018189):

TCGAAAGGATCCTTAATACGACTCACTATAGGGAGACCCAAGCTGGCTAGCCACCATGGGTAAGC
CTATCCCTAACCCTCTCCTCGGTCTCGATTCTACGGGGTCGGCCGCCAGCTGGAGCCACCCTCA
GTTCGAGAAGGGAGGAGGAAGCGGCGGAGGCAGCGGAGGAGGAAGCTGGAGCCACCCGCAGT
TCGAGAAAGGAGCTAGATCAGAGAACCTGTACTTCCAATCCATGTTGCGAGGCTCCGCTTCTTCTA
CAAGTATGGAGAAGGCAAAAGGCAAGGAGTGGACCTCCACAGAGAAGTCGAGGGAAGAGGATCA
GCAGGCTTCTAATCAACCAAATTCAATTGCTTTGCCAGGAACATCAGCAAAGAGAACCAAAGAAAAA
ATGTCTGTCAAAGGCAGTAAAGTGCTCTGCCCTAAGAAAAAGGCAGAGCACACTGACAACCCCAG
ACCTCAGAAGAAGATACCAATCCCTCCATTACCTTCTAAACTGCCACCTGTTAATCTGATTCACCGG
GACATTCTGCGGGCCTGGTGCCAACAATTGAAGCTGAGCTCCAAAGGCCAGAAATTGGATGCATA
TAAGCGCCTGTGTGCCTTTGCCTACCCAAATCAAAAGGATTTTCCTAGCACAGCAAAAGAGGCCAA
AATCCGGAAATCATTGCAAAAAAAATTAAAGGTGGAAAAGGGGGAAACGTCCCTGCAAAGTTCTGA
GACACATCCTCCTGAAGTGGCTCTTCCTCCTGTGGGGGAGCCGCCTGCCCTGGAAAATTCCACTG
CTCTCCTTGAGGGAGTTAATACAGTTGTGGTGACAACTTCTGCCCCAGAGGCTTTGCTGGCCTCCT
GGGCGAGAATTTCAGCCAGGGCGAGGACACCAGAGGCAGTGGAATCTCCACAAGAGGCCTCTG
GTGTCAGGTGGTGTGTGGTCCATGGGAAAAGTCTCCCTGCAGACACAGATGGTTGGGTTCACCTG
CAGTTTCATGCTGGTCAAGCCTGGGTTCCAGAAAAGCAAGAAGGGAGAGTGAGTGCACTCTTCTTG
CTTCCTGCCTCCAATTTTCCACCCCCGCACCTTGAAGACAATATGTTGTGCCCCAAATGTGTTCACA
GGAACAAGGTCTTAATAAAAAGCCTCCAATGGGAATAAGGCGCGGCCGGCCAGCCG

Supplemental Information References

Amemiya HM, Kundaje A, Boyle AP. 2019. The ENCODE Blacklist: Identification of Problematic Regions of the Genome. *Sci Rep* **9**: 9354.

Corces MR, Granja JM, Shams S, Louie BH, Seoane JA, Zhou W, Silva TC, Groeneveld C, Wong CK, Cho SW, et al. 2018. The chromatin accessibility landscape of primary human cancers. *Science* **362**.

Davis ES, Mu W, Lee S, Dozmorov MG, Love MI, Phanstiel DH. 2023. matchRanges: generating null hypothesis genomic ranges via covariate-matched sampling. *Bioinformatics* **39**: btad197.

Dobin A, Davis CA, Schlesinger F, Drenkow J, Zaleski C, Jha S, Batut P, Chaisson M, Gingeras TR. 2012. STAR: ultrafast universal RNA-seq aligner. *Bioinformatics* **29**: 15–21.

Eckersley-Maslin MA, Parry A, Blotenburg M, Krueger C, Ito Y, Franklin VNR, Narita M, D'Santos CS, Reik W. 2020. Epigenetic priming by Dppa2 and 4 in pluripotency facilitates multi-lineage commitment. *Nat Struct Mol Biol* **27**: 696–705. https://www.ncbi.nlm.nih.gov/pubmed/32572255.

Ernst J, Kellis M. 2012. ChromHMM: automating chromatin-state discovery and characterization. *Nat Methods* **9**: 215–216.

Fellmann C, Hoffmann T, Sridhar V, Hopfgartner B, Muhar M, Roth M, Lai DY, Barbosa IAM, Kwon JS, Guan Y, et al. 2013. An Optimized microRNA Backbone for Effective Single-Copy RNAi. *Cell Rep* **5**: 1704–1713.

Gel B, Díez-Villanueva A, Serra E, Buschbeck M, Peinado MA, Malinverni R. 2016. regioneR: an R/Bioconductor package for the association analysis of genomic regions based on permutation tests. *Bioinformatics* **32**: 289–291.

Heinz S, Benner C, Spann N, Bertolino E, Lin YC, Laslo P, Cheng JX, Murre C, Singh H, Glass CK. 2010. Simple Combinations of Lineage-Determining Transcription Factors Prime cis-Regulatory Elements Required for Macrophage and B Cell Identities. *Mol Cell* **38**: 576–589.

Hsiao Y, Zhang H, Li GX, Deng Y, Yu F, Kahrood HV, Steele JR, Schittenhelm RB, Nesvizhskii AI. 2024. Analysis and Visualization of Quantitative Proteomics Data Using FragPipe-Analyst. *J Proteome Res* **23**: 4303–4315.

Kluin RJC, Kemper K, Kuilman T, Ruiter JR de, Iyer V, Forment JV, Cornelissen-Steijger P, Rink I de, Brugge P ter, Song J-Y, et al. 2018. XenofilteR: computational deconvolution of mouse and human reads in tumor xenograft sequence data. *Bmc Bioinformatics* **19**: 366.

Kong AT, Leprevost FV, Avtonomov DM, Mellacheruvu D, Nesvizhskii AI. 2017. MSFragger: ultrafast and comprehensive peptide identification in mass spectrometry–based proteomics. *Nat Methods* **14**: 513–520.

Krueger F, Andrews SR. 2011. Bismark: a flexible aligner and methylation caller for Bisulfite-Seq applications. *Bioinformatics* **27**: 1571–2. https://www.ncbi.nlm.nih.gov/pubmed/21493656.

Lawrence M, Gentleman R, Carey V. 2009. rtracklayer: an R package for interfacing with genome browsers. *Bioinformatics* **25**: 1841–1842.

Li H, Durbin R. 2009. Fast and accurate short read alignment with Burrows–Wheeler transform. *Bioinformatics* **25**: 1754–1760.

Li H, Handsaker B, Wysoker A, Fennell T, Ruan J, Homer N, Marth G, Abecasis G, Durbin R, Subgroup 1000 Genome Project Data Processing. 2009. The Sequence Alignment/Map format and SAMtools. *Bioinformatics* **25**: 2078–2079.

Liao Y, Smyth GK, Shi W. 2013. The Subread aligner: fast, accurate and scalable read mapping by seed-and-vote. *Nucleic Acids Res* **41**: e108–e108.

Lowary PT, Widom J. 1998. New DNA sequence rules for high affinity binding to histone octamer and sequence-directed nucleosome positioning. *J Mol Biol* **276**: 19–42.

Lun ATL, Smyth GK. 2016. csaw: a Bioconductor package for differential binding analysis of ChIP-seq data using sliding windows. *Nucleic Acids Res* **44**: e45–e45.

Machlab D, Burger L, Soneson C, Rijli FM, Schübeler D, Stadler MB. 2022. monaLisa: an R/Bioconductor package for identifying regulatory motifs. *Bioinformatics* **38**: 2624–2625.

Mohammed H, Taylor C, Brown GD, Papachristou EK, Carroll JS, D'Santos CS. 2016. Rapid immunoprecipitation mass spectrometry of endogenous proteins (RIME) for analysis of chromatin complexes. *Nat Protoc* **11**: 316–26. https://www.ncbi.nlm.nih.gov/pubmed/26797456.

Moudgil A, Wilkinson MN, Chen X, He J, Cammack AJ, Vasek MJ, Lagunas T, Qi Z, Lalli MA, Guo C, et al. 2020. Self-Reporting Transposons Enable Simultaneous Readout of Gene Expression and Transcription Factor Binding in Single Cells. *Cell* **182**: 992-1008.e21.

Narita M, Narita M, Krizhanovsky V, Nuñez S, Chicas A, Hearn SA, Myers MP, Lowe SW. 2006. A Novel Role for High-Mobility Group A Proteins in Cellular Senescence and Heterochromatin Formation. *Cell* **126**: 503–514.

Ramírez F, Dündar F, Diehl S, Grüning BA, Manke T. 2014. deepTools: a flexible platform for exploring deep-sequencing data. *Nucleic acids Res* **42**: W187-91.

Smedley D, Haider S, Ballester B, Holland R, London D, Thorisson G, Kasprzyk A. 2009. BioMart – biological queries made easy. *BMC Genom* **10**: 22–22.

Stovner EB, Sætrom P. 2019. epic2 efficiently finds diffuse domains in ChIP-seq data. *Bioinformatics* **35**: btz232.

Suzuki A, Wakaguri H, Yamashita R, Kawano S, Tsuchihara K, Sugano S, Suzuki Y, Nakai K. 2015. DBTSS as an integrative platform for transcriptome, epigenome and genome sequence variation data. *Nucleic Acids Res* **43**: D87–D91.

Tan W, Murphy VJ, Charron A, Twest S van, Sharp M, Constantinou A, Parker MW, Crismani W, Bythell-Douglas R, Deans AJ. 2020. Preparation and purification of mono-ubiquitinated proteins using Avi-tagged ubiquitin. *PLoS ONE* **15**: e0229000.

Teo GC, Polasky DA, Yu F, Nesvizhskii AI. 2021. Fast Deisotoping Algorithm and Its Implementation in the MSFragger Search Engine. *J Proteome Res* **20**: 498–505.

Valero-Mora PM. 2010. ggplot2: Elegant Graphics for Data Analysis. *J Stat Softw* **35**.

Vasaikar SV, Straub P, Wang J, Zhang B. 2017. LinkedOmics: analyzing multi-omics data within and across 32 cancer types. *Nucleic Acids Res* **46**: gkx1090-.

Yu F, Deng Y, Nesvizhskii AI. 2025. MSFragger-DDA+ enhances peptide identification sensitivity with full isolation window search. *Nat Commun* **16**: 3329.

Yu F, Haynes SE, Nesvizhskii AI. 2021. IonQuant Enables Accurate and Sensitive Label-Free Quantification With FDR-Controlled Match-Between-Runs. *Mol Cell Proteom* **20**: 100077.

Yu G, Wang L-G, Han Y, He Q-Y. 2012. clusterProfiler: an R Package for Comparing Biological Themes Among Gene Clusters. *OMICS: A J Integr Biol* **16**: 284–287.

Zhang Y, Liu T, Meyer CA, Eeckhoute J, Johnson DS, Bernstein BE, Nusbaum C, Myers RM, Brown M, Li W, et al. 2008. Model-based analysis of ChIP-Seq (MACS). *Genome Biol* **9**: R137.
